# Supplementary material for: Scalable electrosynthesis of commodity chemicals from biomass by suppressing non-Faradaic transformations
Source: Nat Commun. 2023 Sep 12;14:5621. doi: 10.1038/s41467-023-41497-y (PMC10497620; doi:10.1038/s41467-023-41497-y)
Supplement: Supplementary file 1 — Supplementary Information [file 41467_2023_41497_MOESM1_ESM.pdf]

Supplementary Information

Scalable electrosynthesis of commodity chemicals from biomass by suppressing non-Faradaic transformations

Hua Zhou[1,2,3,7], Yue Ren[1,7], Bingxin Yao[1], Zhenhua Li[1], Ming Xu[1], Lina Ma[4], Xianggui Kong[1], Lirong Zheng[5], Mingfei Shao[1,3], Haohong Duan*[2, 6]

Supplementary Notes

Supplementary Note 1 Effect of non-Faradaic degradation during glucose oxidation reaction (GOR)

To quantitatively assess the influence of non-Faradaic transformations, we studied the kinetics of glucose conversion in 50 mL 1 M KOH solution with 100 mM substrate under electrochemical (1.5 V vs RHE) and chemical (i.e., without applying bias and electrocatalyst) conditions, respectively. During glucose electrolysis, excellent carbon balance (93%−98%) were obtained (Supplementary Fig. 11), indicating that most products from glucose transformation were reliably detected. As shown in Supplementary Fig. 11b, a portfolio of intermediates (arabinose, fructose, gluconate) and products, including targeted formate and other organic acids (i.e., lactate, glycerate, glycolate, gluconate, oxalate, and tartronate) are generated. Notably, the transformation of glucose to fructose or lactate is not oxidation reaction, but base-catalyzed isomerization, retro-aldol, dehydration, and Cannizzaro rearrangement reactions (Supplementary Figs. 12, 13)[1-4]. The result suggests that the electrocatalytic glucose conversion is accompanied by complex non-Faradaic side-reactions of reactant and intermediates occurred in bulk electrolyte. The total reaction velocity (ν_{total}) of glucose at initial stage was estimated to be 5.44×10^{-6} mol L^{-1} s^{-1} (Supplementary Fig. 11a), which is contributed by both electrocatalytic and non-Faradaic fraction.

Furthermore, the reaction velocity of non-Faradaic reaction (ν_{nF}), that is base-catalyzed glucose degradation, was calculated to be 1.64×10^{-6} mol L^{-1} s^{-1}

(Supplementary Fig. 12; in 1 M KOH solution without applying bias nor electrocatalyst). Based on the catalytic results under electrochemical conditions (at 1.5 V vs RHE) and under chemical conditions (without applying bias nor electrocatalyst), we estimate that about 54% of the converted glucose fraction was contributed by non-Faradaic side-reactions during electrolysis (scenario B at 1.5 V vs RHE). These results indicate that the non-Faradaic degradation of glucose overpower the targeted electrocatalytic conversion in high-concentration and large-volume reaction system (i.e., 100 mM glucose in 50 mL electrolyte). In addition, kinetic studies of the electrooxidation of other biomass-derived polyols and aldose also reveal that the carbon loss is mainly contributed by the non-Faradaic degradation of unstable reactant/intermediates to byproducts (Supplementary Fig. 14). These results demonstrate that non-Faradaic degradation is a non-negligible issue in electrocatalytic oxidative C−C bond cleavage of biomass-derivatives to formate under scale-up relevant conditions, even outpace the targeted electrocatalysis.

Supplementary Note 2 Probing unstable intermediates during electrocatalytic GOR

The set-up of combining SPCFR system and HPLC is shown in Supplementary Fig. 33. Notably, the feedstock solutions of electrolyte (2 M KOH) and glucose (200 mM) were separately stored to supress base-catalyzed non-Faradaic degradation. A single-module SPCFR, as shown in Supplementary Fig. 19, was applied for electrocatalytic glucose oxidation to formate using a mixed electrolyte composed of 1 M KOH and 100 mM glucose at a flow rate of 11.4 mL min^{-1}. The experiments were operated at different currents (0−7 A) to observe the variation of possible reaction intermediates. Importantly, the electrolyte at the outlet of SPCFR was collected and immediately quenched by dilute acid (0.5 M H_2SO_4) to stabilize reactive species for subsequent HPLC analysis.

As shown in Supplementary Fig. 34a, the peak of formate (11) grows with the increasing of current. In the meantime, peaks of a series of aldehydes (arabinose (3), erythrose (6), glycolaldehyde (7), and formaldehyde (10)) and glycolic acid (8) are observed and grows with the increasing of current until 4 A, reaching an equilibrium

between the formation of these intermediates and their further conversion to final formate at higher current (>4 A). As the only one obviously detected aldonic acid in SPCFR, glycolic acid can be excluded as the main intermediate of dominant reaction route to formate during GOR in following studies (Supplementary Note 3). Based on these results, we speculate that aldehydes are the real intermediate of C−C cleavage to give formate during electrooxidation of glucose, denoted as an aldose route (Route I).

By contrast, fructose (2), glyceric acid (4), and lactic acid (9) are present in the HPLC chromatograms of the electrolyte from batch reactor as results of non-faradic reactions (Supplementary Fig. 24). Notably, the peak of glyceraldehyde (5) and glyceric acid (4) almost located at the same position. Considering the extremely unstable property of glyceraldehyde and long reaction time in batch reactor, we attributed the detected peak at ~11.57 min in the HPLC chromatogram to glyceric acid (Supplementary Fig. 24). Considering the severe non-Faradaic side-reactions in batch reactor, these detected aldonic acids cannot be used to support the most adopted aldonic acid rout (denoted as Route II).

Supplementary Note 3 Reaction pathway for C−C bond cleavage of glucose to formate

To validate our above hypothesis, we performed isotope experiments using ^{13}C1-labeled glucose (Supplementary Fig. 37) and ^{13}C1-labeled gluconate (Supplementary Fig. 38) as the substrates. As shown in Supplementary Fig. 37, ^{13}C-labeled formate was produced when ^{13}C1-labeled glucose was used as the substrate, indicating that the aldehyde group in glucose was converted to formate. In addition, semiquantitative ^{1}H NMR analysis shows that the ratio of ^{13}C/^{12}C in the generated formate from ^{13}C1-labeled glucose reached 0.3 (higher than the theoretical value of 0.2) at low charge, and it successively decreased at longer reaction time (Supplementary Fig. 39). This trend can be explained by that the C1−C2 bond cleavage in glucose is more favourable, thus the ^{13}C1-labeled aldehyde group was firstly converted to formate. This is in well agreement with our previous theoretical calculation that glucose electrooxidation is initiated at C1−C2 position to give arabinose and formate, owing to the smallest bond

order among the five C−C bonds[5].

In contrast, the ratio of $^{13}C/^{12}C$ in the generated formate from ^{13}C1-labeled gluconate is about 0.01 (equal to the natural abundance of $^{13}C/^{12}C$) in the whole electrolysis process (Supplementary Fig. 39). This result reveals that the acid group of gluconate cannot be converted to formate, thus route II can be excluded. Collectively, these results further demonstrated that the aldehydes, rather than acids, are the real intermediates of C−C bond cleavage to formate in glucose electrooxidation.

To eliminate the possible route II, we performed electrooxidation experiments on the claimed acid precursors (glycolic acid, glyceric acid, and lactic acid) in route II to generate formate (Supplementary Fig. 40). Glycolic acid has been detected during the electrooxidation of EG[6-8], glycerol[9-16], glucose and other biomass-derived polyhydroxy compounds (Fig. 4a, Supplementary Table 4) to formate. Similarly, glyceric acid is also detected in the electrooxidation of glycerol, erythritol, xylitol, xylose, sorbitol and glucose (Supplementary Table 4). Thus, they are usually proposed as the key intermediates in oxidative C−C cleavage to formate in previous reports[9-17], especially in the extensively studied glycerol electrooxidation. In theory, the oxidation number (Supplementary Table 7) of the carbon atom located at C1 position in the aldonic acid is +3, higher than that of generated formate (+2). Thus, the oxidation number analysis indicates that converting the carboxy group in aldonic acid to formate is a reduction reaction, which is unreasonable occurred on anode.

A simple experimental method is carbon balance analysis of the electrooxidation of glycolic acid, glyceric acid, lactic acid, and corresponding polyols to identify if the carboxy group in aldonic acid can stream into formate. As shown in Supplementary Fig. 40, excellent carbon balance (90−100%) are obtained when using the polyols as substrate, much higher than that (50−63%) using the acids as substrate. The most possibility is that the electrooxidation of the acid molecule produces CO_2 originated from the carboxy group. Overall, we can exclude glycolic acid and glyceric acid as the main intermediate in electrooxidation of glucose, glycerol, and other biomass derived polyhydroxy compounds to formate. These results imply that the non-faradaic by-products (i.e., aldonic acids) have been misinterpreted as the intermediate of

electrocatalytic polyhydroxy compounds conversion to formate in many previous reports.

Supplementary Note 4 Preparation of lignocellulosic sugars and crude glycerol

Regarding lignocellulosic saccharification, the main objective is to extract glucose and xylose from holocellulose (cellulose and hemicellulose) in pristine wood (birch wood) via fractionation process (Supplementary Fig. 49), as feedstocks for downstream electrolysis. Specifically, the wood was treated under reflux conditions in aqueous formic acid solution to obtain liquor stream and solid cellulose pulp. Most of the hemicellulose (>99%) and small fraction of cellulose (5.2%) in the wood were hydrolyzed to monosaccharides into the liquor, with additional 92.7% of lignin (Supplementary Figs. 49−51). The liquor was then subject to evaporation, water dissolution, and organic extraction to yield solid lignin, bio-oil, and xylose enriched sugar solution (Supplementary Fig. 49). The solid cellulose pulp was saccharified by commercial enzyme blend (Cellic® CTec2) to produce glucose with yield of 94% (Supplementary Fig. 50b). Then, the obtained lignocellulosic sugars were fed into the flow-cell-stack, eventually producing formate with selectivity of 73.1% (Supplementary Fig. 51).

Regarding biodiesel production, it is known that crude glycerol can be obtained as a by-product during biodiesel production in large amounts[18, 19]. To integrate with downstream electrolysis, a KOH catalyzed process was adopted for transesterification of soybean oil with methanol (Supplementary Fig. 52), generating 86.3 wt.% of isolated biodiesel and 9.79 wt.% yield of glycerol. Then, the obtained low-grade glycerol was subject to electrolysis using the flow-cell-stack, finally giving formate with selectivity of 76.3%.

Supplementary Figures

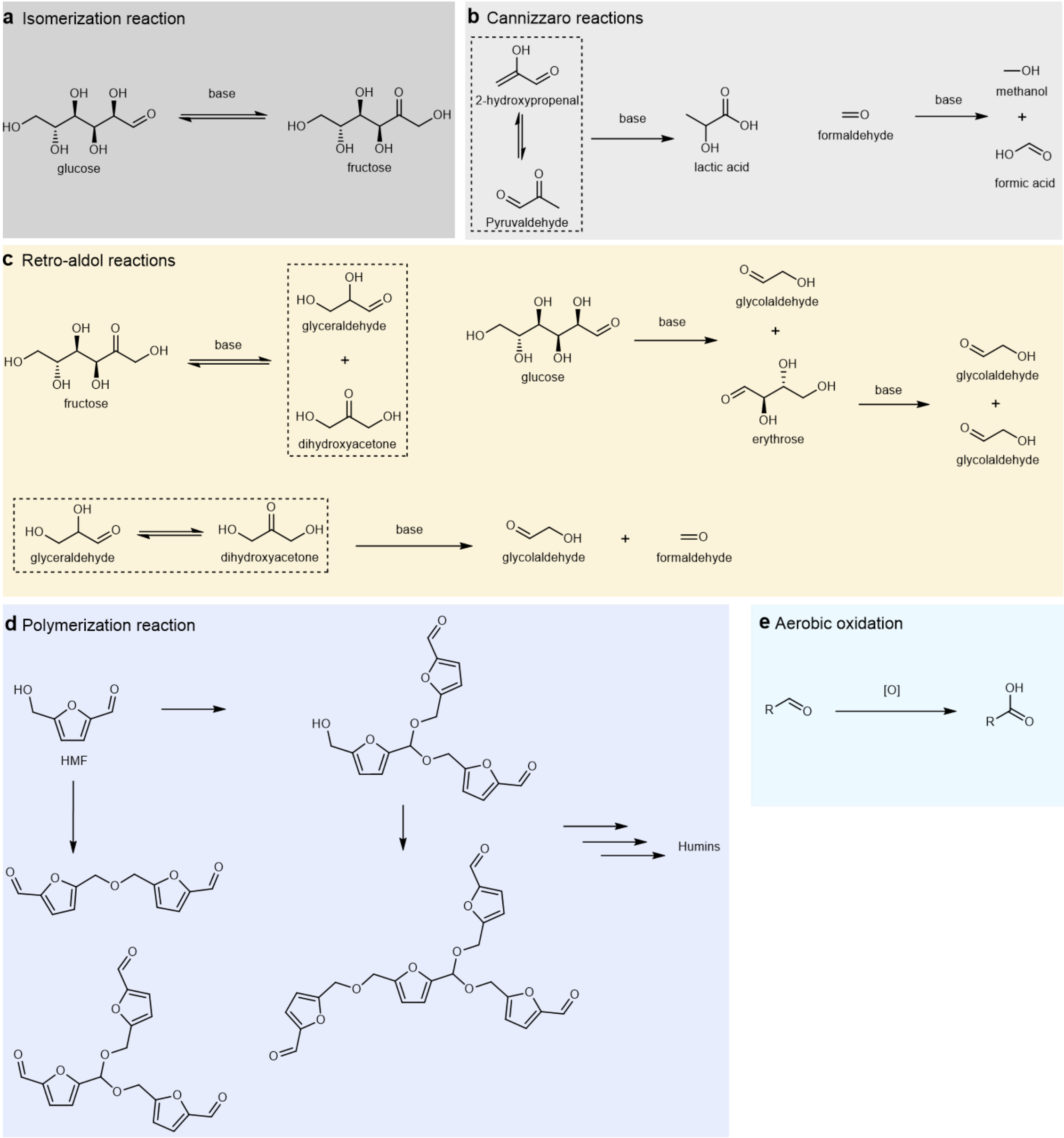

Supplementary Figure 1. Typical non-faradaic reactions of biomass-derived platforms in alkaline electrolyte. a Base catalyzed aldose-ketose isomerization reaction[20]. **b** Base catalyzed intramolecular and intermolecular Cannizzaro reactions[21]. **c** Base catalyzed retro-aldol reactions of aldoses and ketones[20, 22, 23]. **d** HMF degradation via self-polymerization[24, 25]. **e** Aerobic oxidation of aldehydes[22, 26].

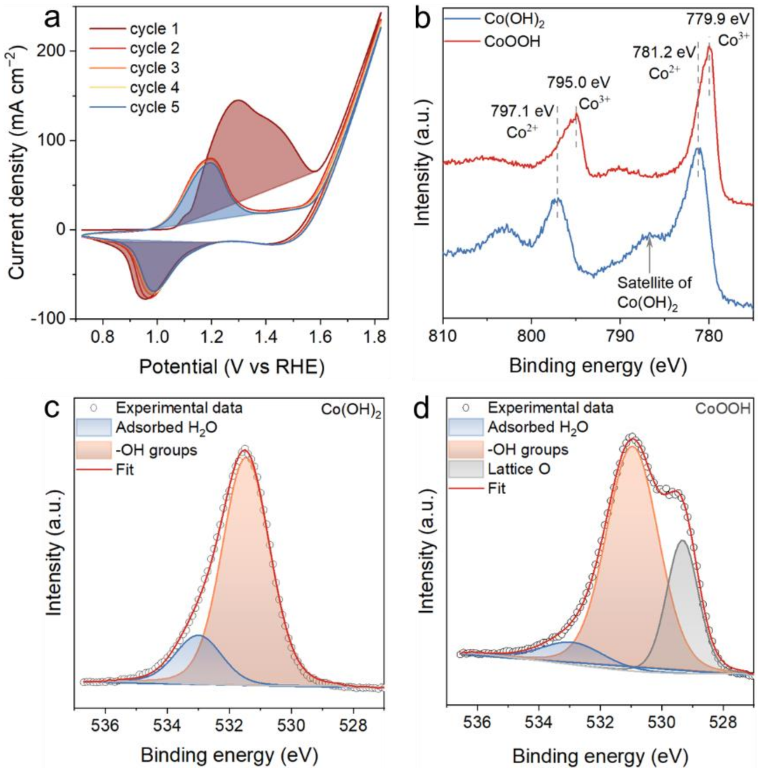

Supplementary Figure 2. Synthesis of CoOOH from $Co(OH)_2$. a CV curves for activation of $Co(OH)_2$ to CoOOH. **b−d** Co 2*p* and O 1*s* XPS spectra of $Co(OH)_2$ and CoOOH. a.u.: arbitrary units.

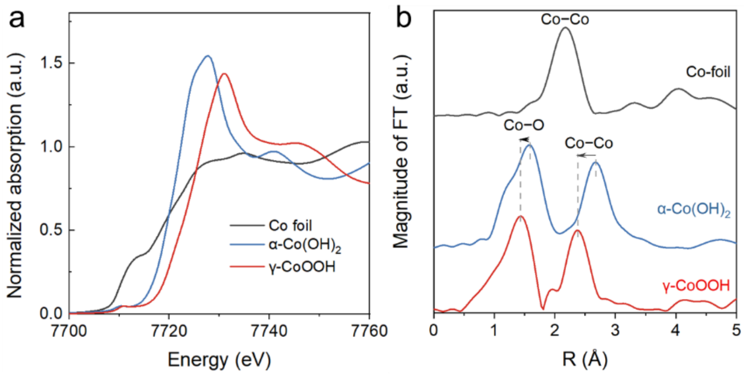

Supplementary Figure 3. Co K-edge XANES and EXAFS results. a Co K-edge XANES **b** EXAFS spectra of $Co(OH)_2$ and CoOOH.

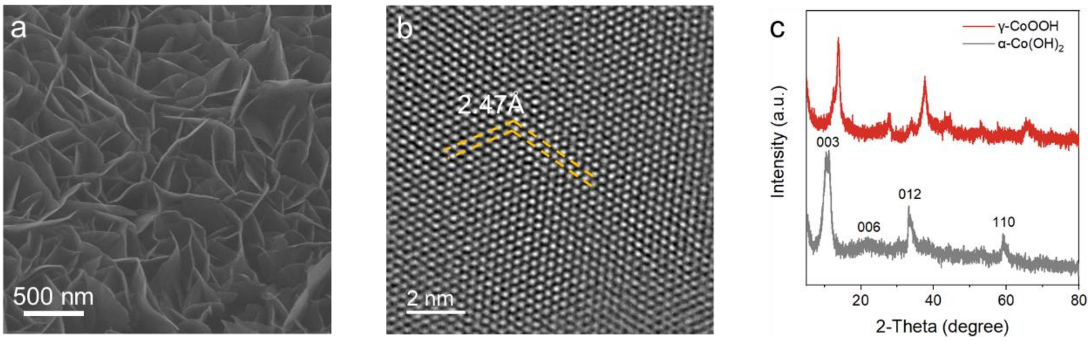

Supplementary Figure 4. Characterization of CoOOH. a SEM image, **b** HR-TEM image, and **c** XRD of CoOOH.

After electrodeposition, the as-prepared $Co(OH)_2$ was oxidized to CoOOH by CV method (Supplementary Fig. 2a). This process was identified by X-ray photoelectron spectroscopy (XPS, Supplementary Fig. 2b−d) and X-ray adsorption fine structure spectroscopy (XAFS, Supplementary Fig. 3)[5]. A self-supported CoOOH nanoarray on nickel foam was observed by scanning electron microscopy (Supplementary Fig. 4a). High-resolution transmission electron microscopy (HR-TEM) image of the sample (Supplementary Fig. 4b) displays an interplanar spacing of 2.47 Å, which corresponds to the (110) plane of hexagonal γ-CoOOH, and it is further verified by the X-ray diffraction (XRD, Supplementary Fig. 4c)[27]. Overall, these complementary techniques confirm the successful synthesis of γ-CoOOH.

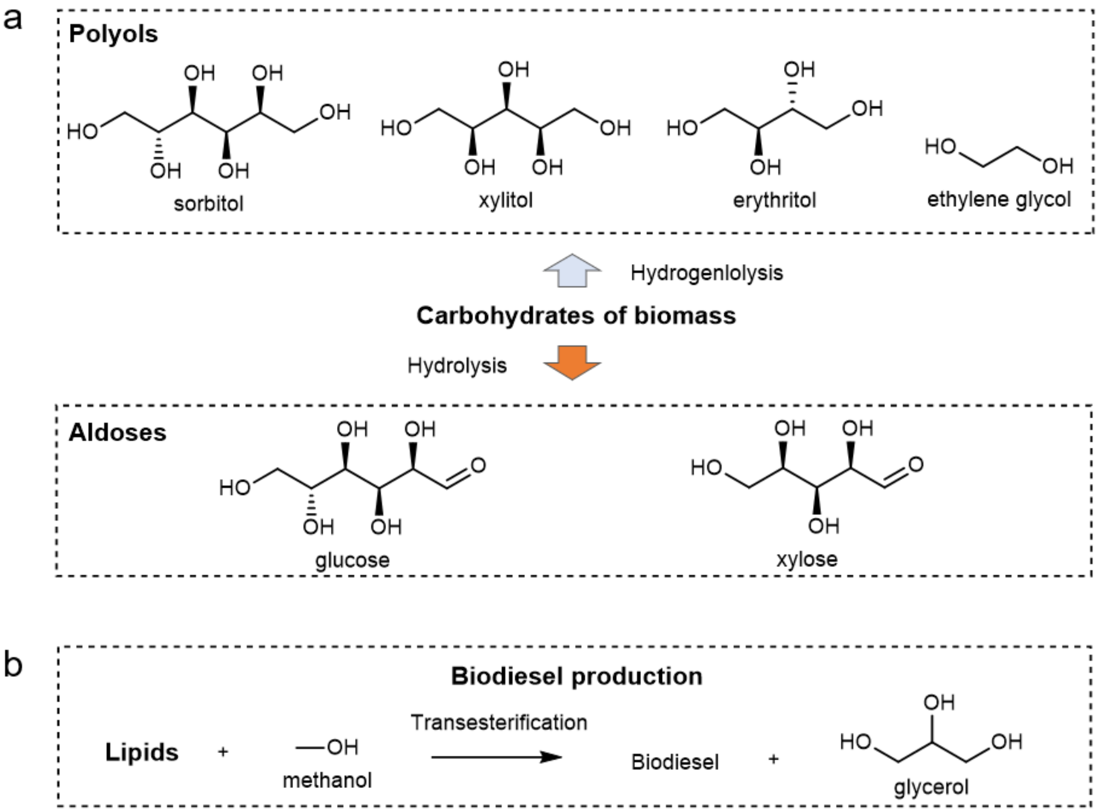

Supplementary Figure 5. Polyhydroxy compounds (polyols and aldoses) preparation from biomass. **a** Polyols and aldoses prepared from carbohydrates in biomass via hydrogenolysis[28-30] and hydrolysis[31, 32], respectively. **b** Glycerol obtained from biodiesel production as a by-product.

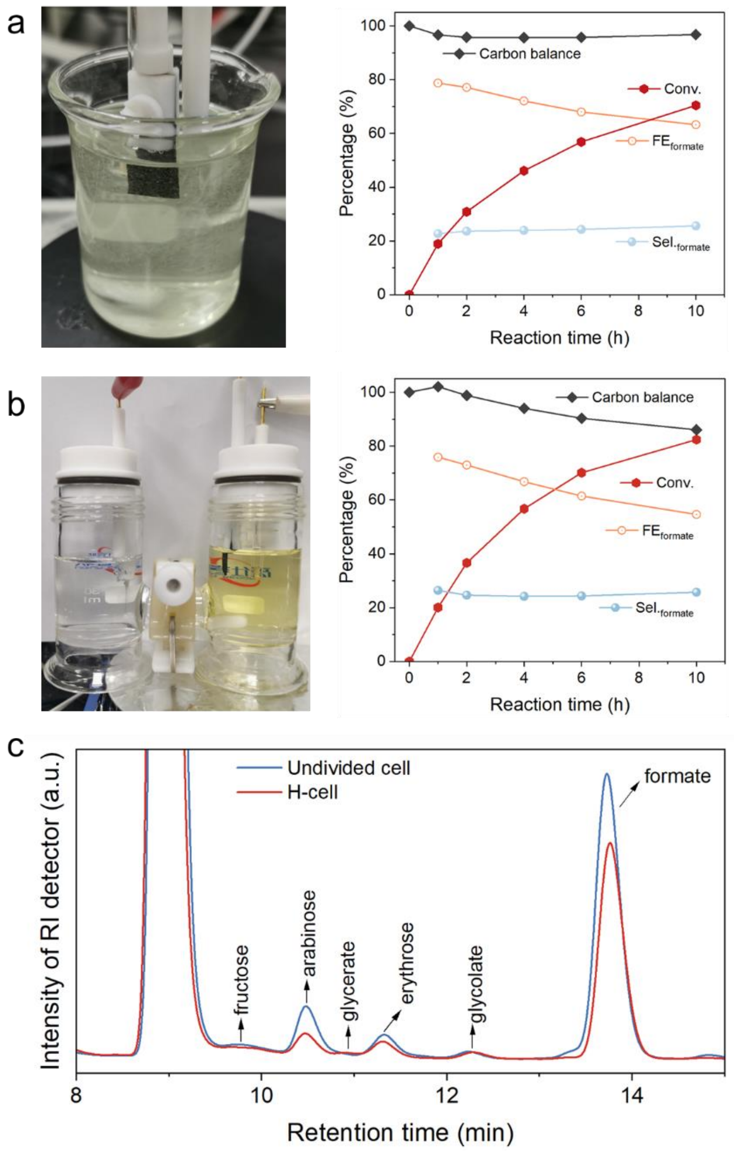

Supplementary Figure 6. Photographs of two batch reactors and corresponding kinetic curves for GOR. **a** Photograph of undivided cell. Electrolyte conditions: 50 mL, 1 M KOH, 100 mM glucose. **b** Photograph of H-cell. Anolyte conditions: 40 mL, 1 M KOH, 100 mM glucose. Catholyte conditions: 40 mL 1 M KOH. The H-cell is separated by an anion exchange membrane (AEM, FAA-3-50). Reaction conditions: anode: 1 cm^2 CoOOH/NF, cathode: Pt foil, reference electrode: saturated Ag/AgCl, applied potential: 1.5 V vs RHE. The electrolyte was stirred by a magnetic stirring apparatus at 800 rpm during reaction. **c** HPLC chromatograms of catalytic products using an undivided cell and a H-cell at four-hours of reaction.

The kinetic curves show that no significant changes of the formate selectivity between H-cell and undivided cell during electrocatalytic GOR. In addition, HPLC

chromatograms show that similar products distribution was obtained by using undivided cell and H-cell, without observing associated reduction products (e.g., polyols) from glucose and intermediates (Supplementary Fig. 6c), suggesting that glucose and associated intermediates and products may not be reduced in the absence of membrane under our reaction conditions. Together with the negligible effects of organic substrates (i.e., HMF, glucose) on hydrogen evolution reaction at cathode according to previous literatures[33, 34] and high-cost and stability issue of membranes (especially for AEM), we adopted an undivided cell in the following studies.

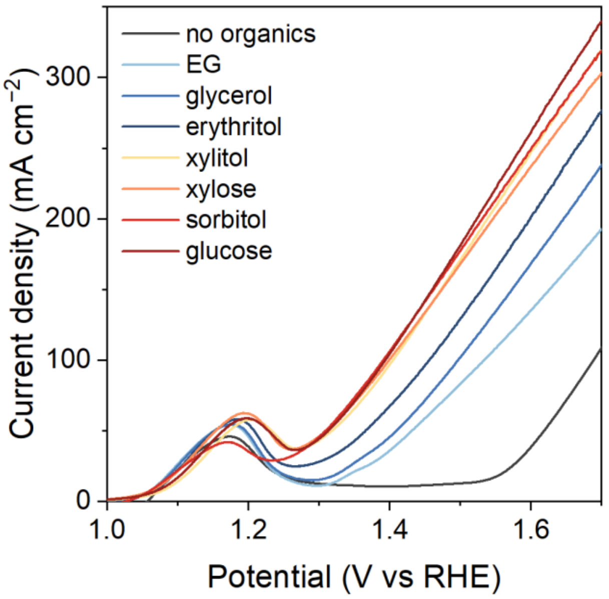

Supplementary Figure 7. Linear sweep voltammetry (LSV) curves. Linear sweep voltammetry (LSV) curves of CoOOH/NF for electrooxidation of biomass-derived polyhydroxy compounds in 1 M KOH electrolyte with 100 mM substrates at a scan rate of 10 mV s^{-1}.

LSV curves suggest that the CoOOH/NF electrocatalyst exhibits oxidation activity for these biomass-derived polyhydroxy compounds at low bias range (1.2−1.4 V vs RHE), prior to OER potential window (>1.55 V vs RHE).

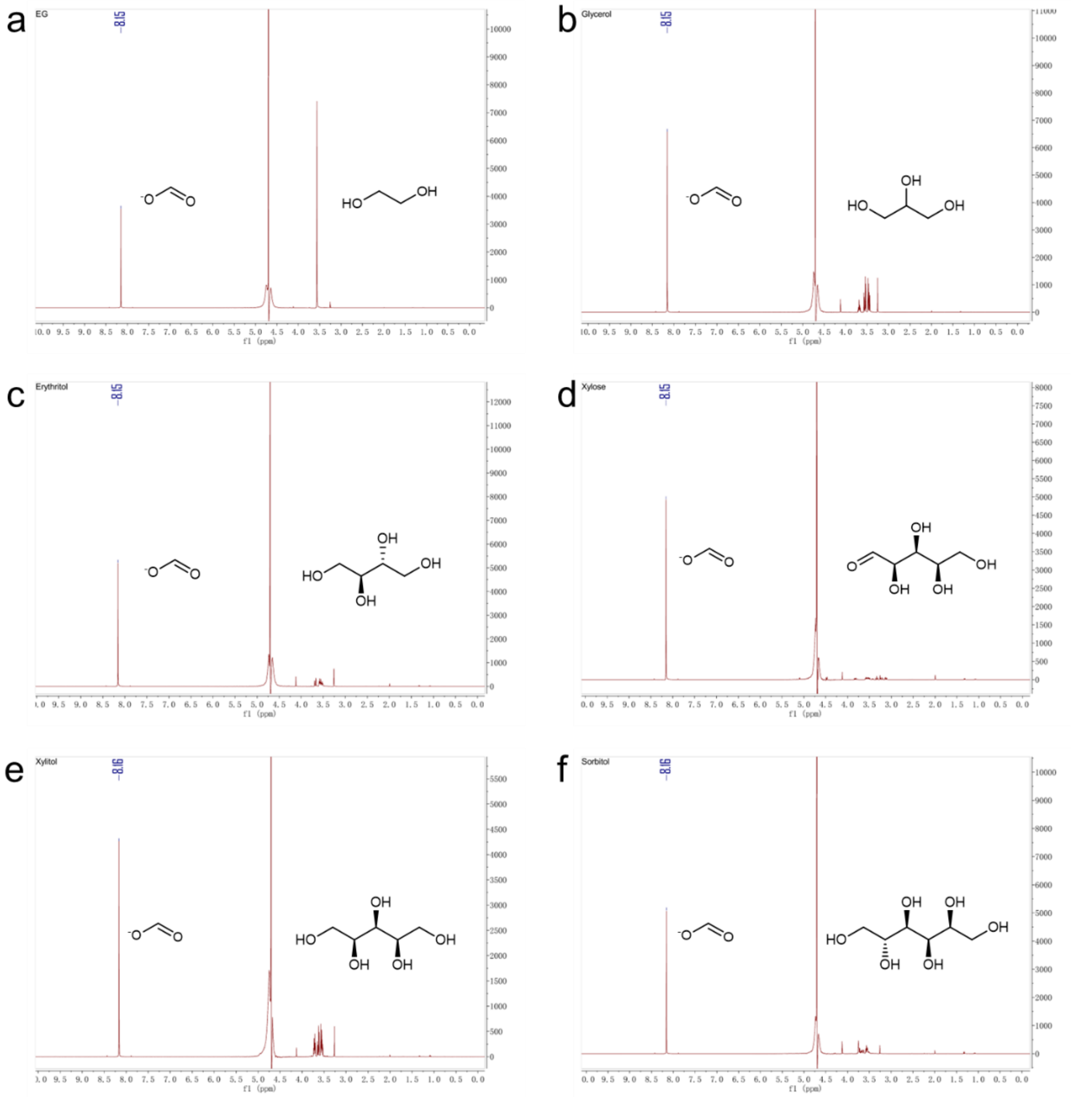

Supplementary Figure 8. ^{1}H nuclear magnetic resonance (^{1}H NMR) spectra. ^{1}H nuclear magnetic resonance (^{1}H NMR) spectra of the electrolyte of various polyhydroxy compounds after electrooxidation at 1.5 V vs RHE for 6 hours, including **a** ethylene glycol, **b** glycerol, **c** erythritol, **d** xylose, **e** xylitol and **f** sorbitol.

^{1}H NMR analysis shows that formate is the main product during electrooxidation of these polyhydroxy compounds, suggesting that these substrates may proceed via similar oxidative C−C bonds rupture process.

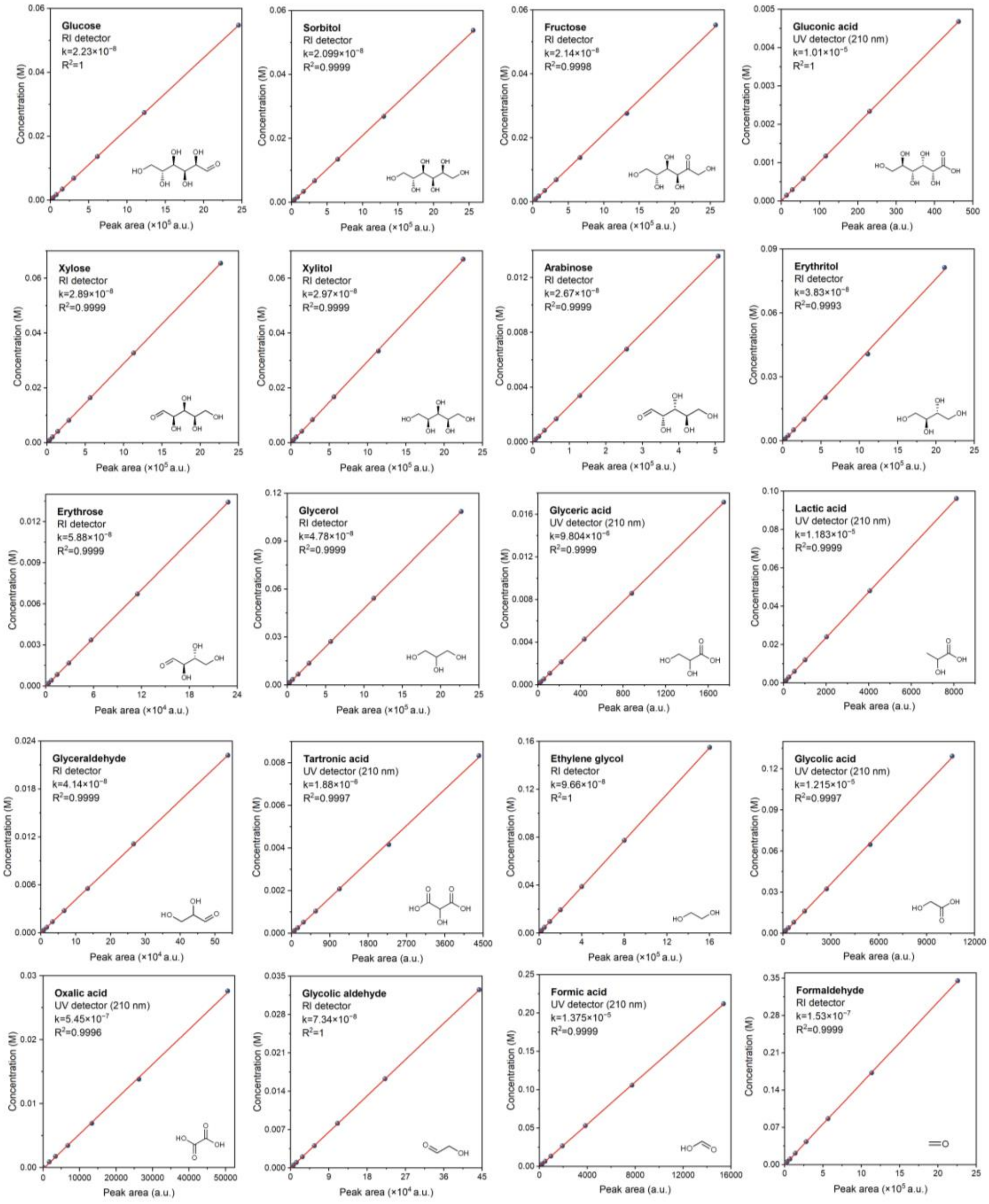

Supplementary Figure 9. Standard curves for HPLC analysis. Standard curves of polyhydroxy compounds and corresponding derivatives for HPLC analysis. Corresponding HPLC conditions are shown in Supplementary Table 3.

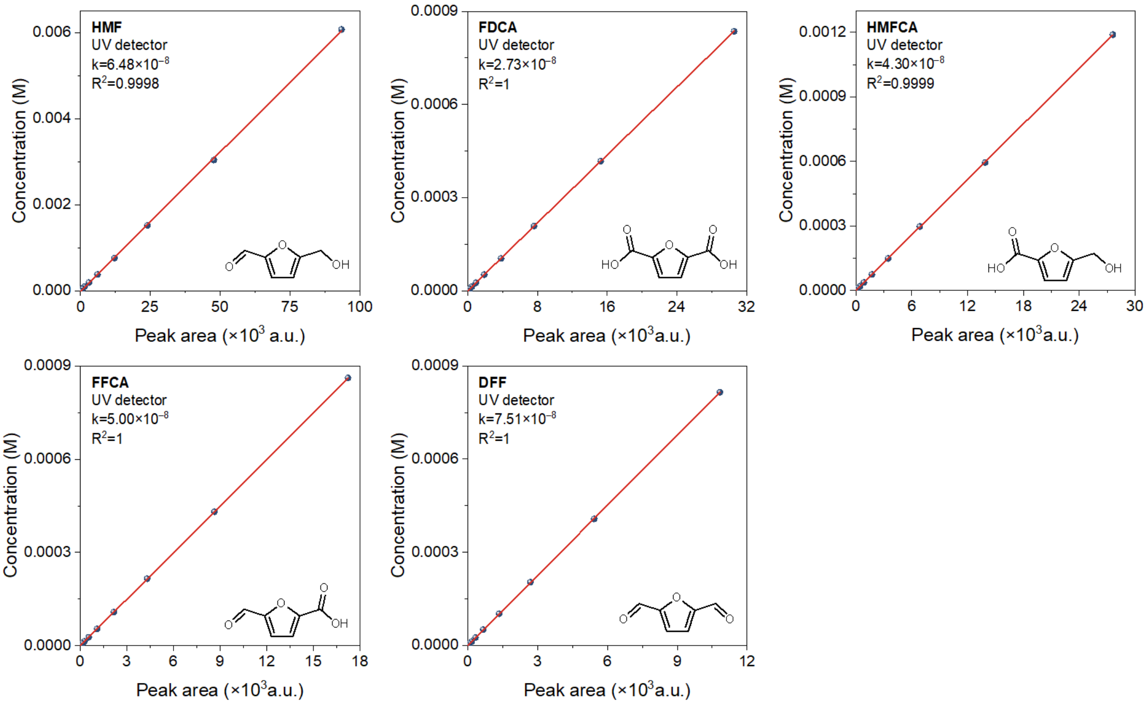

Supplementary Figure 10. Standard curves of HMF and corresponding derivatives for HPLC analysis. Corresponding HPLC conditions are shown in Supplementary Table 3.

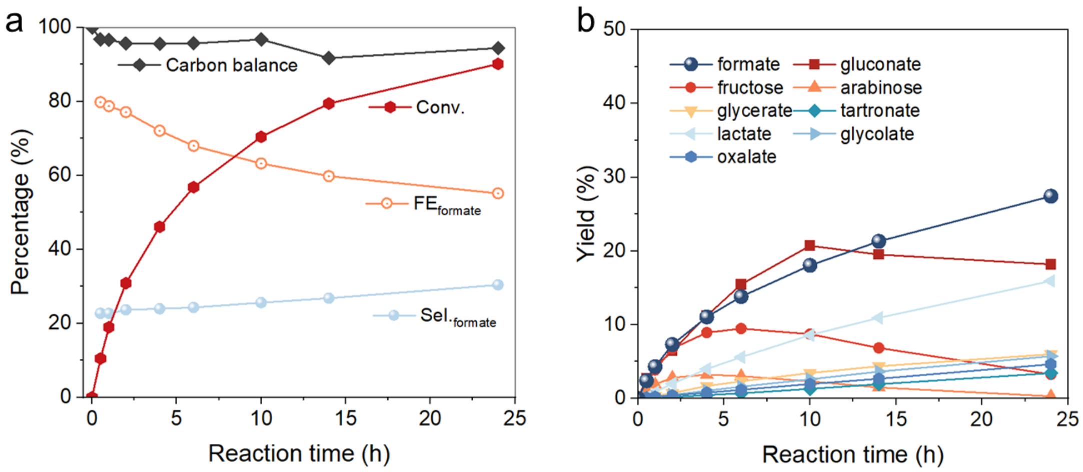

Supplementary Figure 11. Kinetics of glucose conversion in electrochemical conditions. a Conversion, carbon balance, FE and selectivity of formate during electrooxidation of glucose. **b** Yield of various products from glucose. Reaction conditions: 100 mM glucose in 50 mL 1 M KOH electrolyte, CoOOH/NF and Pt as anode and cathode, respectively, at 1.5 V vs RHE.

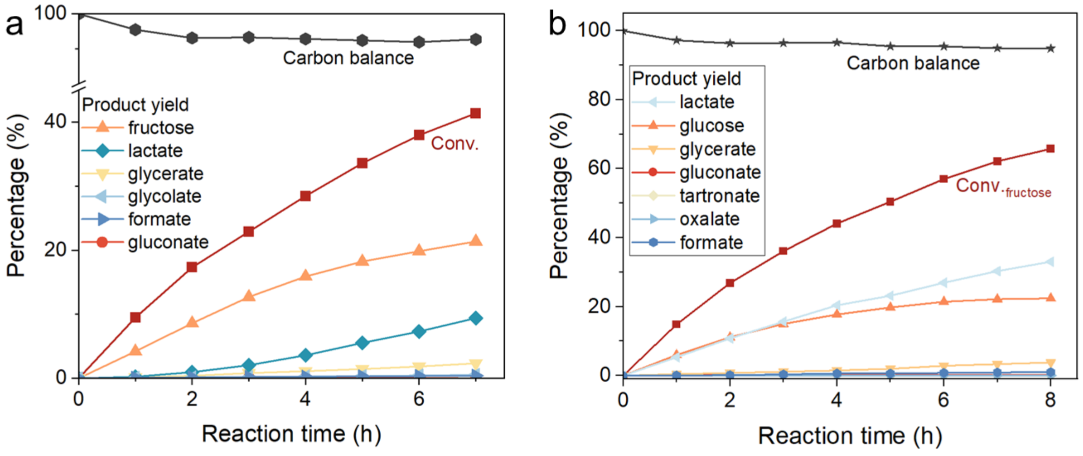

Supplementary Figure 12. Sugar degradation kinetic curves in 1 M KOH solution at room temperature. a Glucose (100 mM) degradation and products formation in 1 M KOH solution with the reaction time. **b** Fructose (100 mM) degradation and products formation in 1 M KOH solution with the reaction time.

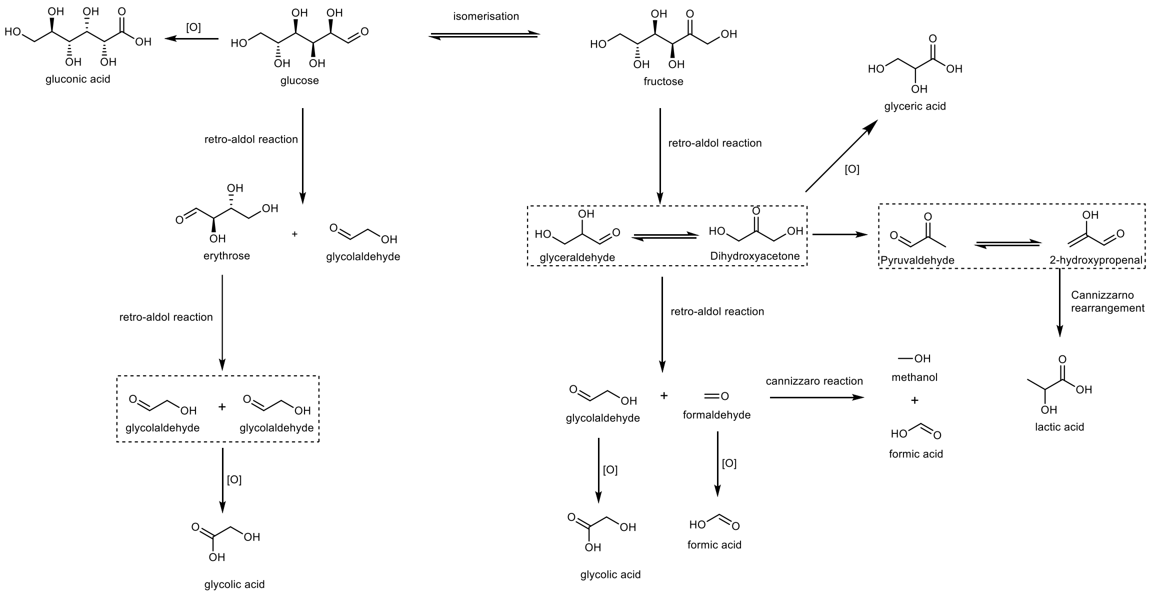

Supplementary Figure 13. Proposed reaction pathways. Proposed reaction pathways of glucose degradation in non-deaerated KOH solution based on experiments and literatures[20, 22, 26].

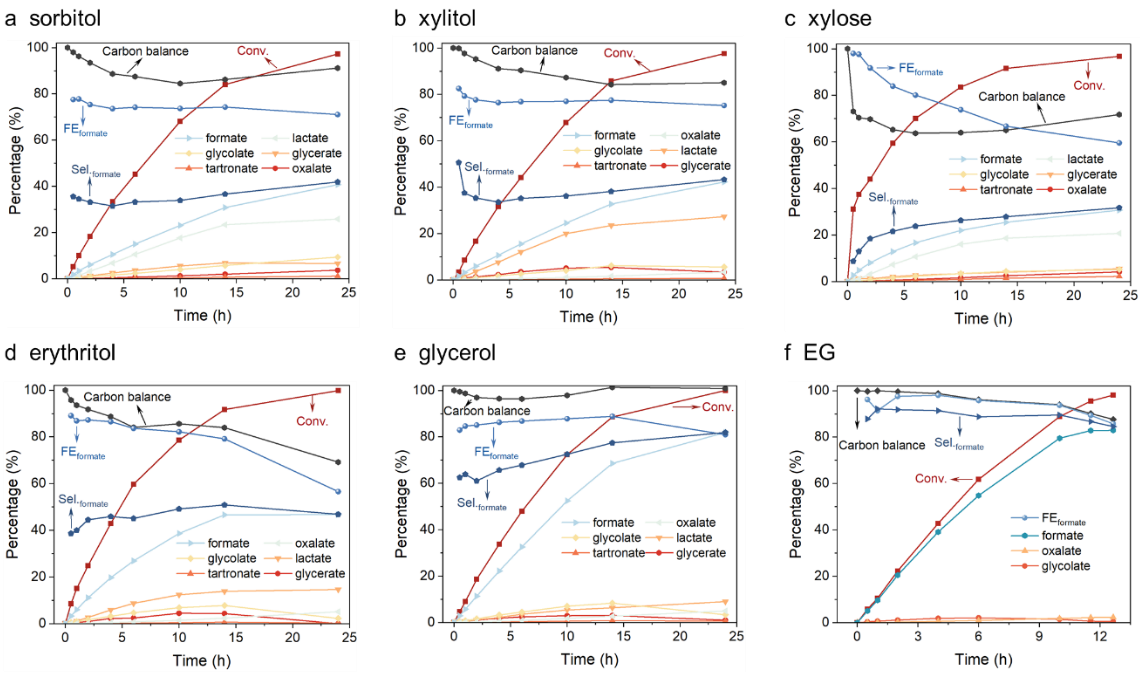

Supplementary Figure 14. Catalytic performances of electrooxidation of various biomass-derived polyhydroxy compounds in batch reactor. The biomass-derived polyhydroxy compounds include **a** sorbitol, **b** xylitol, **c** xylose, **d** erythritol, **e** glycerol and **f** ethylene glycol. Reaction conditions: 100 mM substrate in 50 mL 1 M KOH electrolyte, CoOOH/NF and Pt as anode and cathode, respectively, at 1.5 V vs RHE.

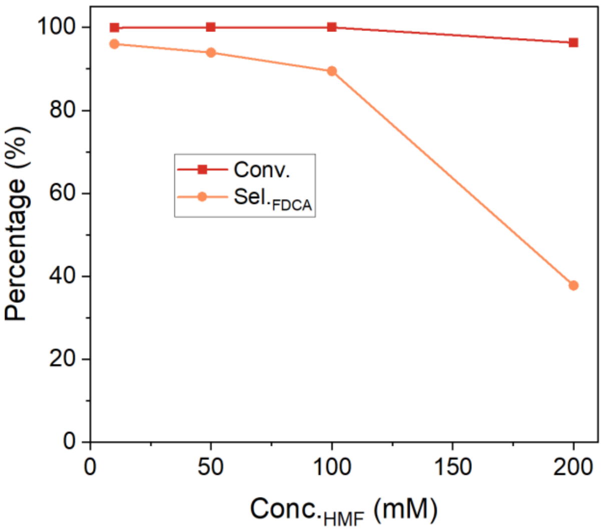

Supplementary Figure 15. Catalytic performances of HMF oxidation at different concentration (10−200 mM) in an H-cell at 1.5 V vs RHE. Reaction conditions: anolyte: 40 mL 1 M KOH electrolyte containing different concentration of HMF; catholyte: 40 mL 1 M KOH; the analyte and catholyte were separated by AEM; anode:

1 cm^2 CoOOH/NF; cathode: Pt foil; reference electrode: saturated Ag/AgCl.

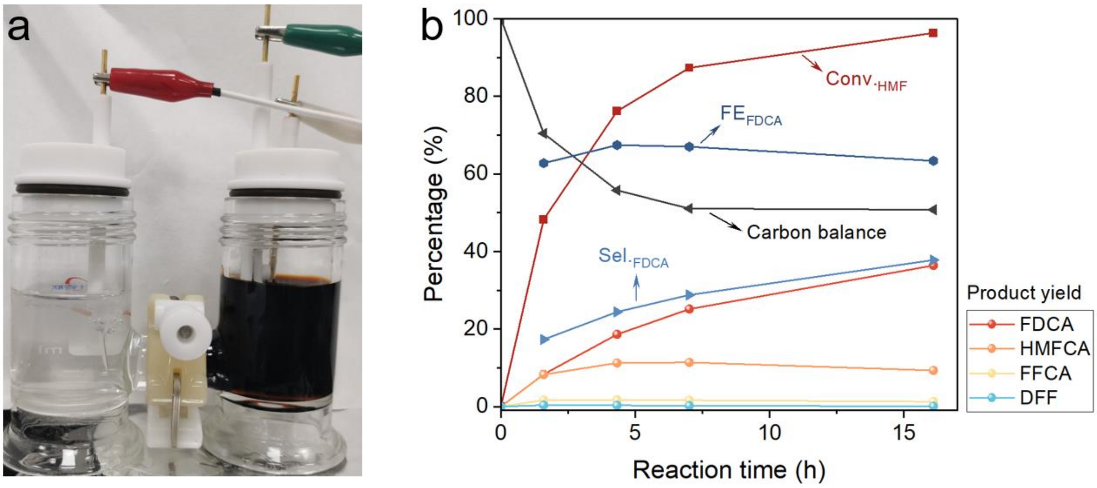

Supplementary Figure 16. Electrocatalytic concentrated HMF oxidation in a H-cell. a Photograph of coloured analyte. **b** HMF conversion and product formation as a function of reaction time at 1.5 V vs RHE. HMF conversion (red rectangular points and line). Product yields: circular points and lines. Carbon balance: black triangular points and line. FDCA selectivity: blue triangular points and line. FDCA faradaic efficiency: circular points and line. Reaction conditions: analyte: 40 mL 1 M KOH electrolyte containing 200 mM HMF; catholyte: 40 mL 1 M KOH; the analyte and catholyte were separated by AEM; anode: 1 cm^2 CoOOH/NF; cathode: Pt foil; reference electrode: saturated Ag/AgCl.

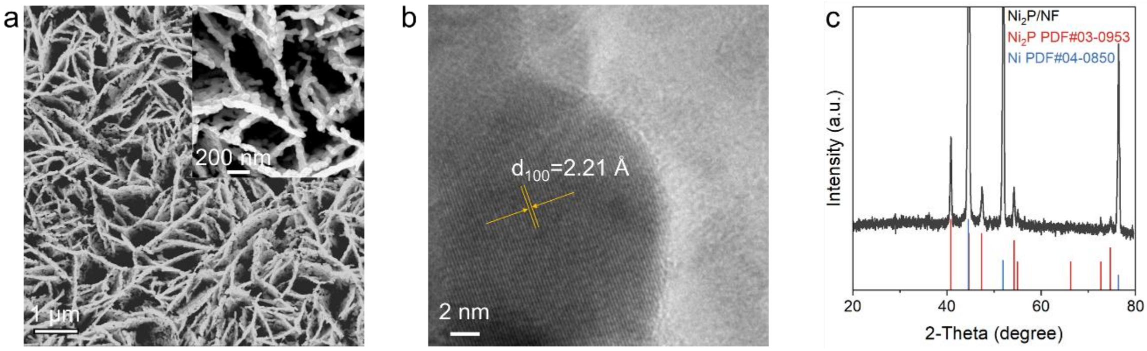

Supplementary Figure 17. Characterization of Ni_2P. a SEM images of Ni_2P nano-array. Inset: enlarged region showing interconnected nano-particles. **b** High-resolution TEM image of Ni_2P nano-particle. **c** XRD pattern of Ni_2P/NF.

As shown in Supplementary Figure 17a, SEM images revealed nano-array structure of Ni_2P, and the nano-array is composing of interconnected nano-particles

(inset). TEM combined with XRD characterizations (Supplementary Fig. 17b, c) further confirmed the Ni_2P structure.

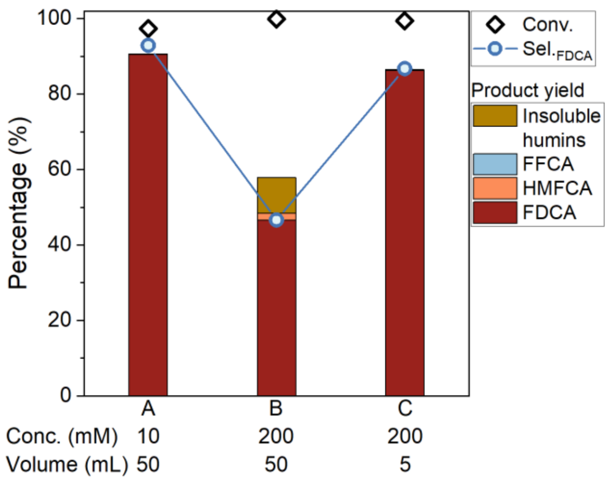

Supplementary Figure 18. Catalytic performances of HMF electrooxidation. Catalytic performances of HMF electrooxidation in different scenarios (different feedstock concentrations and electrolyte volumes) at 1.5 V vs RHE using Ni_2P/NF as the anode.

As shown in Supplementary Fig. 18, FDCA selectivity dramatically decreased when HMF electrolyte with higher concentration (200 mM) and larger volume (50 mL) was used. Specifically, FDCA selectivity decreased from 92.9% (scenario A) and 86.7% (scenario C) to 46.6% (scenario B). Therefore, we can conclude that the similar trend in Fig. 2 was observed when extensively used anodic catalyst was used (Supplementary Fig. 18), indicating that the carbon loss issue in HMF electrooxidation is not mainly related to the selection of catalyst.

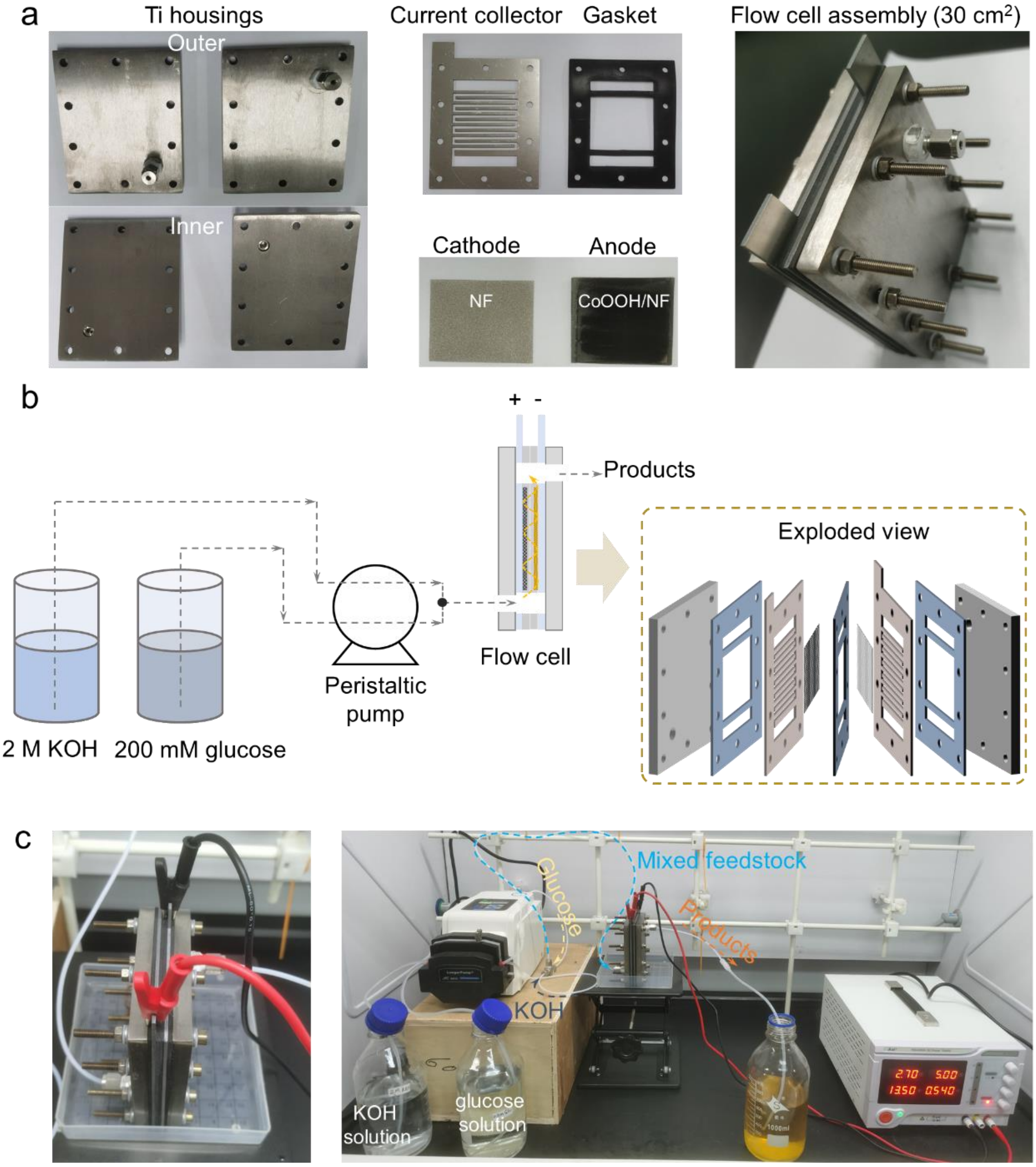

Supplementary Figure 19. Single-module SPCFR system. **a** Photographs of cell components and assembled cell. **b** Illustration of the single-module SPCFR system. **c** Photographs of operated single-module SPCFR system.

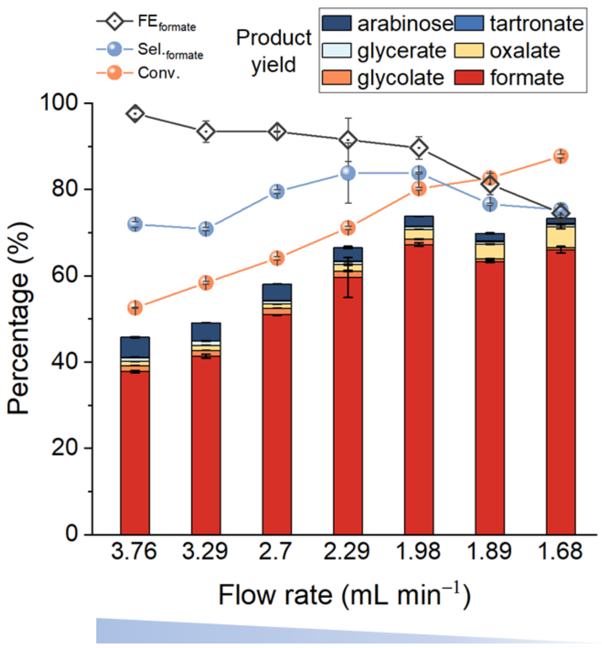

Supplementary Figure 20. Catalytic performances of the single-module SPCFR. Catalytic performances of the single-module SPCFR for GOR as a function of flow rate at current of 3 A. Error bars correspond to the standard deviation of three measurements.

As the flow rate decreases, the duration time of substrate and intermediates in the reactor increases, leading to higher conversion of glucose and intermediates (such as arabinose). After optimizing flow rate, we obtained the highest formate yield (67.2%) and selectivity (83.8%) at 1.98 mL min^{-1}. At lower flow rate, the oxygen evolution and formate overoxidation become more competitive, resulting in the decrease of formate FE and selectivity.

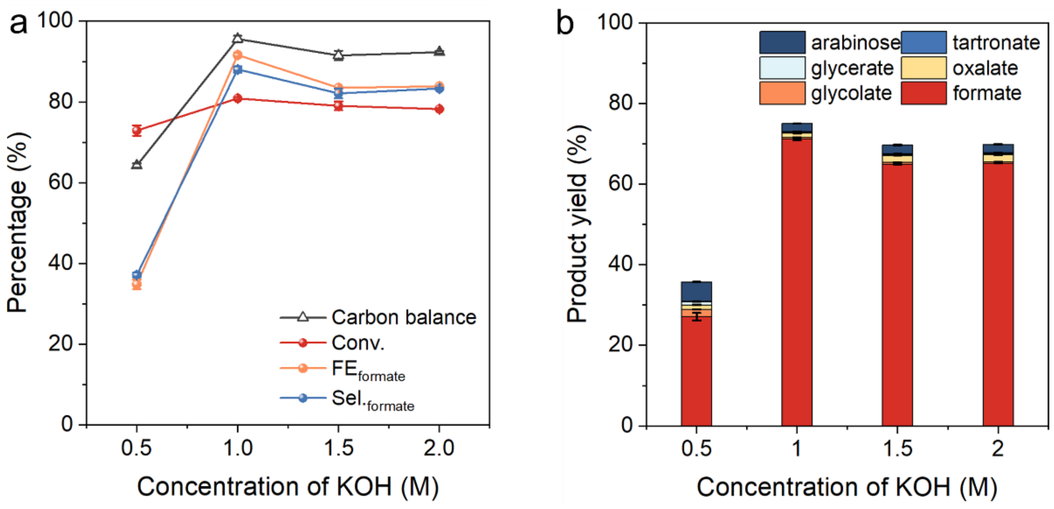

Supplementary Figure 21. Glucose electrooxidation in different concentration of KOH electrolyte. a Catalytic performances of glucose electrooxidation. **b** Yield of various product. Reaction conditions: single-module SPCFR system, current: 3 A, flow rate: 1.98 mL min^{-1}, glucose concentration: 100 mM. The desired composition of electrolyte was controlled by one multichannel peristaltic pump to mix 200 mM glucose solution and KOH solution with different concentrations.

To optimize the ratio of glucose/KOH, we evaluated the catalytic performances of glucose electrooxidation at a fixed concentration (100 mM) but with different concentration of KOH electrolyte (from 0.5 to 2 M). As shown in Supplementary Fig. 21, inferior catalytic performances (e.g., FE and selectivity of formate is <40%) were obtained in 0.5 M KOH electrolyte. This can be explained by the overoxidation of formate to carbonate, as large quantity of bubbles was generated when the electrolyte was acidified for HPLC analysis. In contrast, good catalytic performances can be achieved (the FE and selectivity of formate is >80%) after KOH concentration was increased to 1 M. These results indicate that optimizing the ratio of glucose/KOH is important to obtain high catalytic performance.

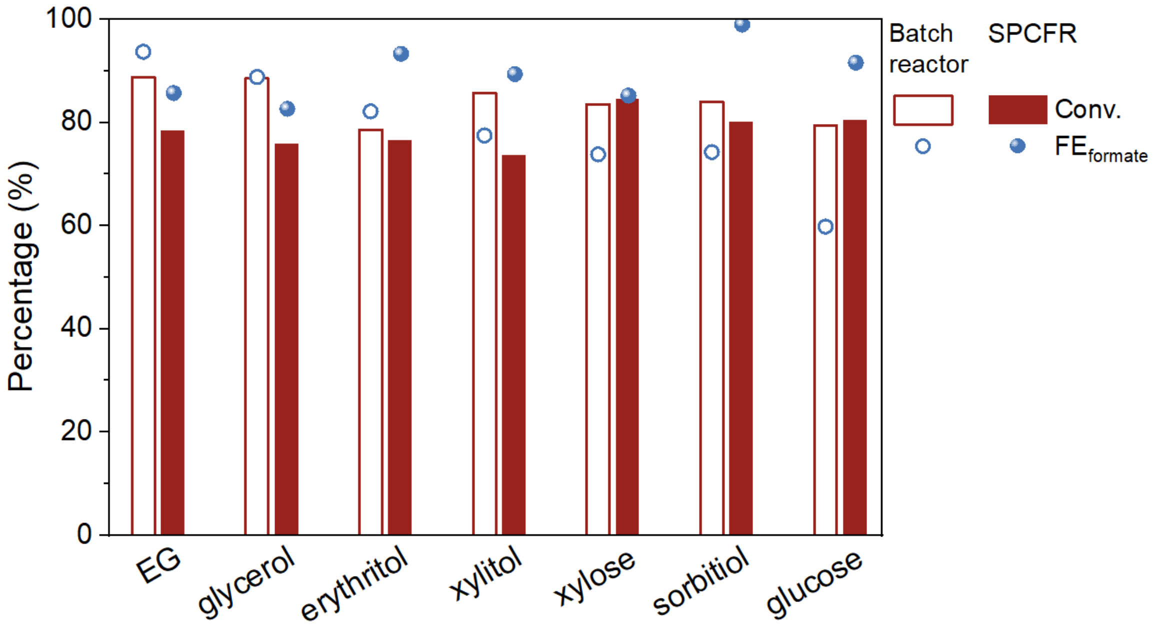

Supplementary Figure 22. Catalytic results using batch reactor and SPCFR. Conversion of biomass-derived sugars and polyols (with increased carbon numbers)

and FE to formate using batch reactor and SPCFR.

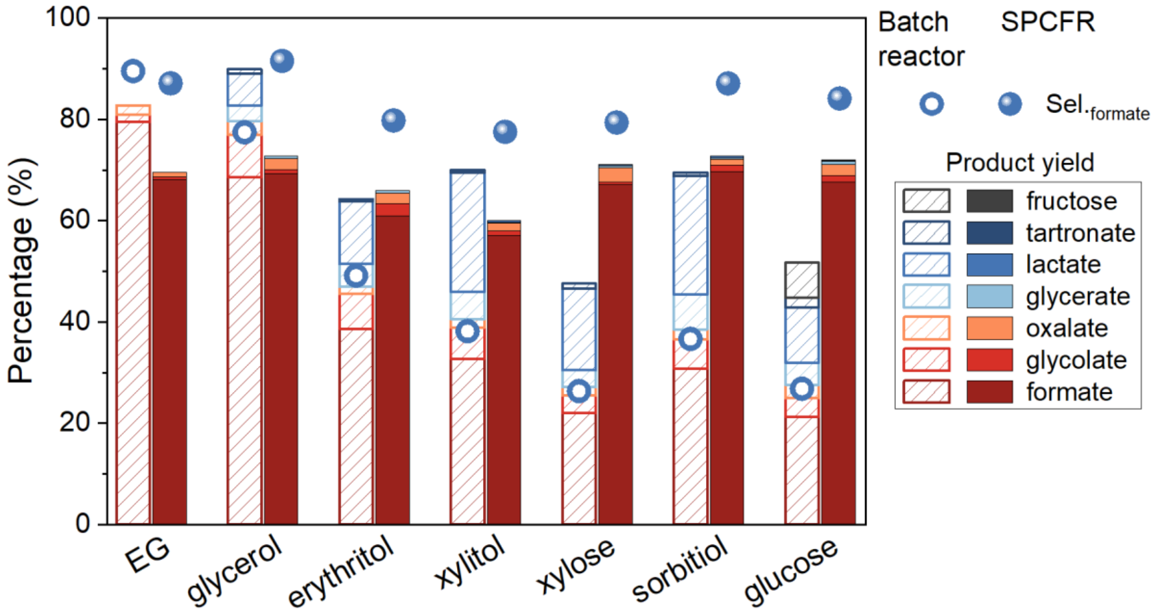

Supplementary Figure 23. Product yield and formate selectivity of SPCFR and batch reactor. Product yield and formate selectivity of SPCFR and batch reactor on the electrooxidation of biomass derived sugars and polyols.

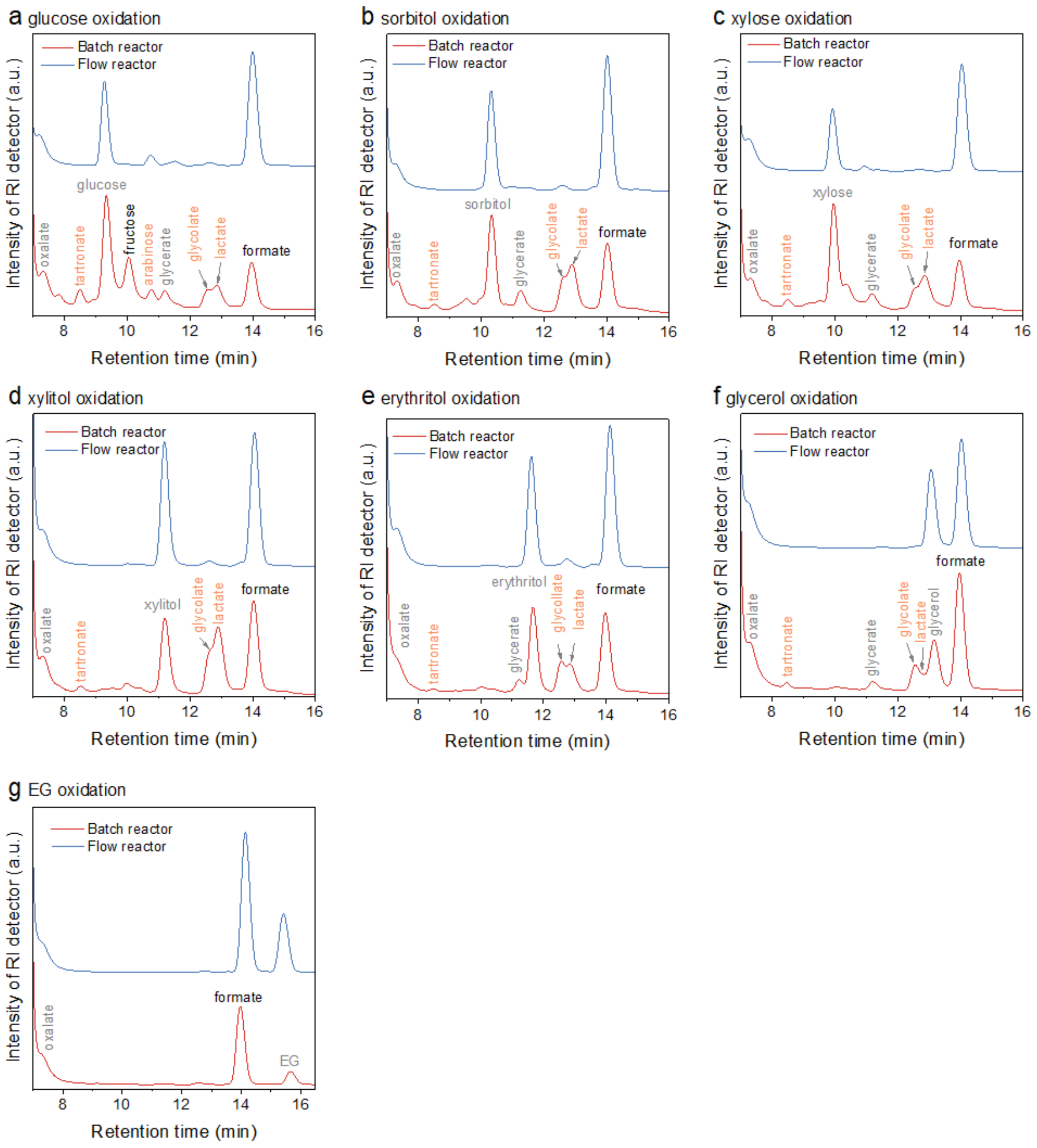

Supplementary Figure 24. HPLC chromatograms results. HPLC chromatograms of the electrolyte from batch reactor and SPCFR system for various biomass-derived polyhydroxy compounds electrooxidations, including **a** glucose, **b** sorbitol, **c** xylose, **d** xylitol, **e** erythritol, **f** glycerol and **g** ethylene glycol.

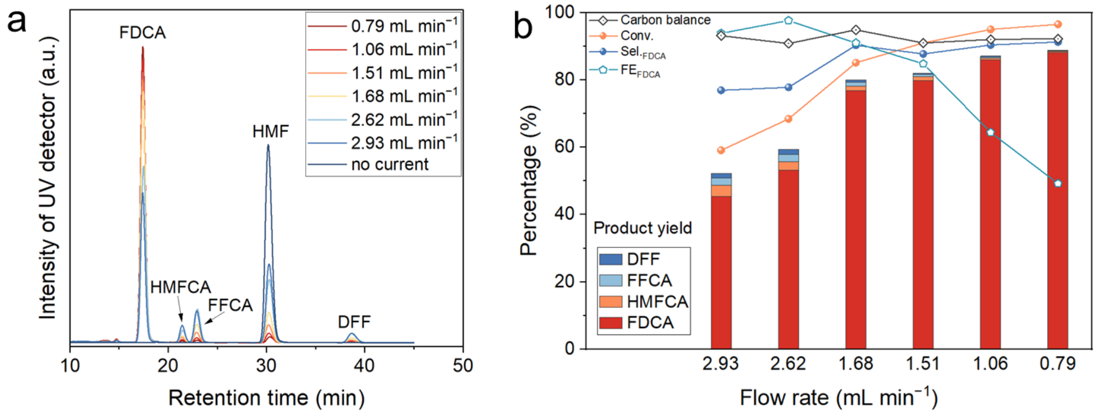

Supplementary Figure 25. Electrocatalytic HMF oxidation in SPCFR at 3 A. a HPLC chromatograms of electrolyte at different flow rate. **b** Catalytic performances of the single-module SPCFR for HMF oxidation as a function of flow rate at current of 3 A.

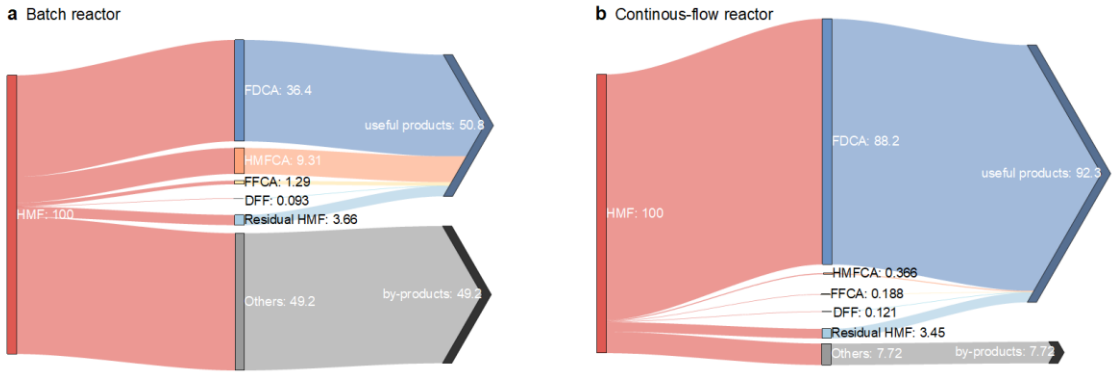

Supplementary Figure 26. Mass balance analysis. Mass balance analysis of electrocatalytic HMF oxidation in **a** batch reactor and **b** SPCFR.

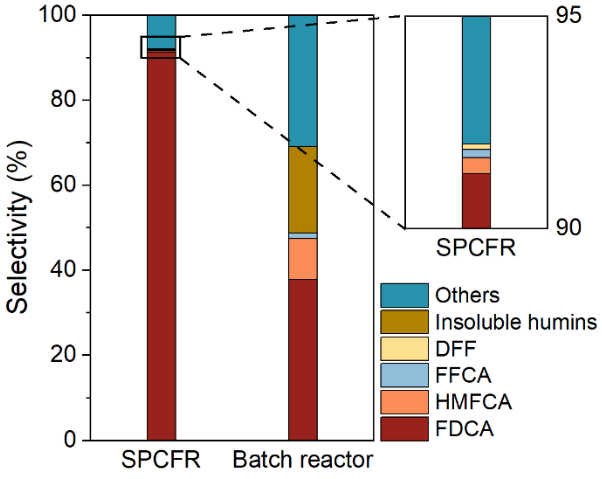

Supplementary Figure 27. Selectivity in batch reactor and SPCFR. Selectivity of

detectable products and other unknown by-products. Inset enlarges the products with very low selectivity.

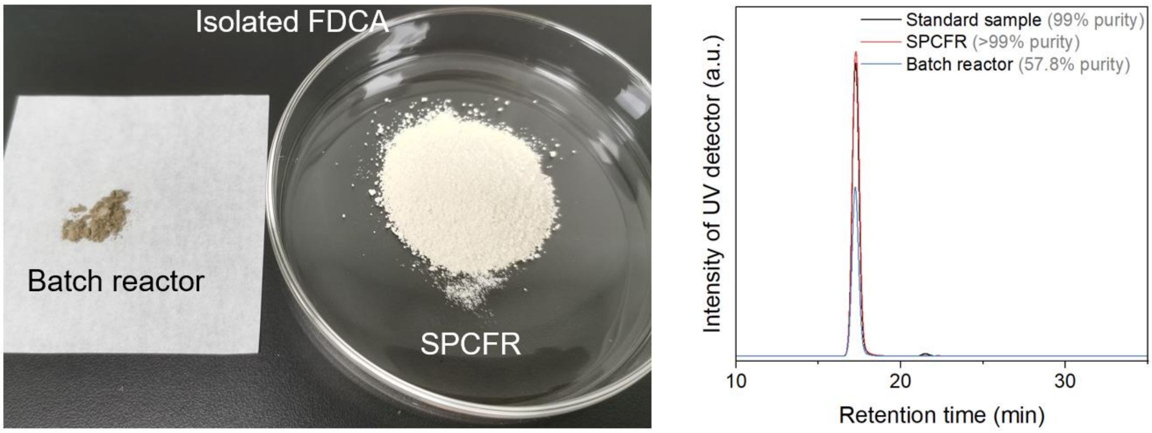

Supplementary Figure 28. Product analysis of batch reactor and SPCFR. Photograph of isolated FDCA for batch reactor and SPCFR (left) and corresponding HPLC chromatograms at 0.44 g L^{-1} (right).

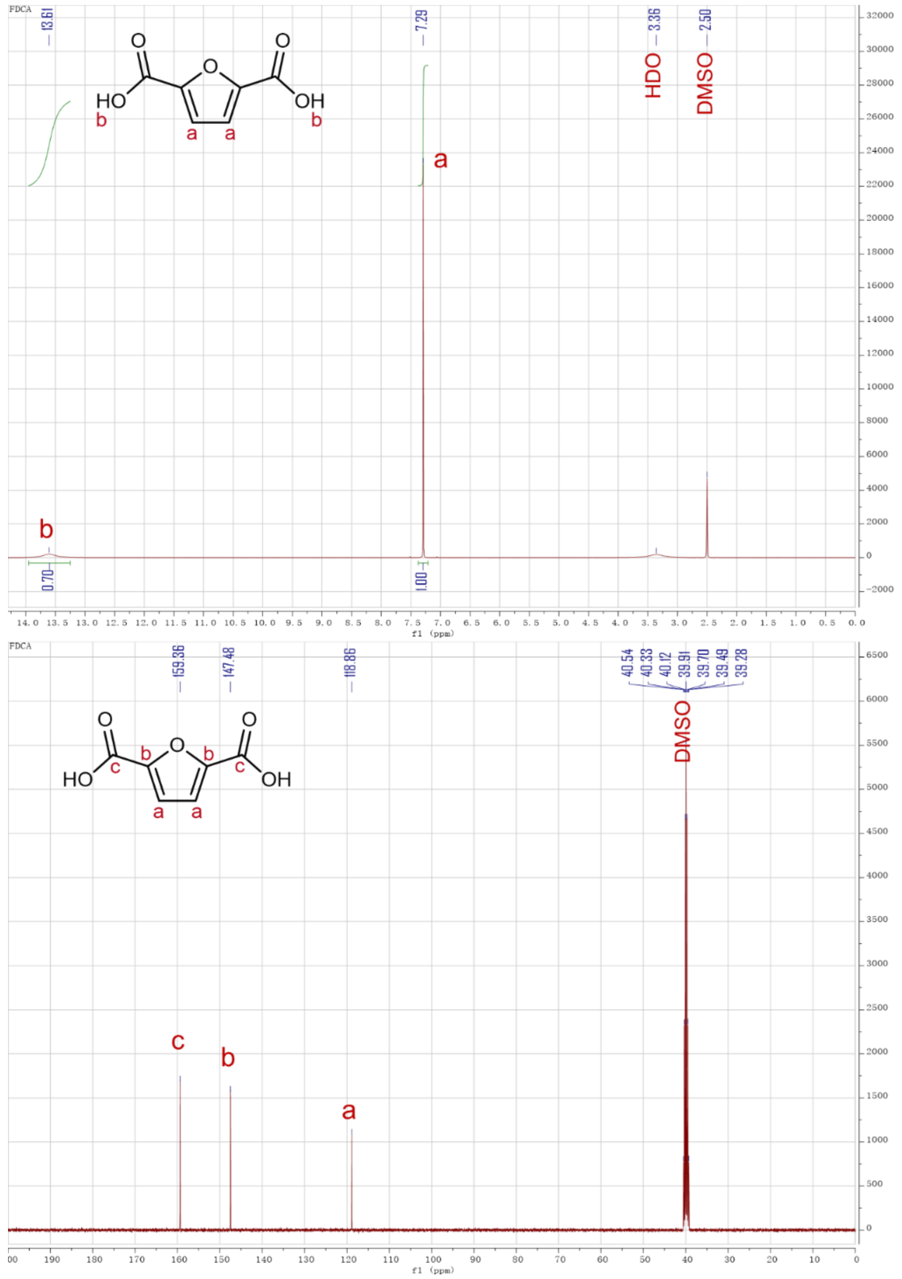

Supplementary Figure 29. NMR analysis. NMR analysis of self-prepared FDCA by SPCFR.

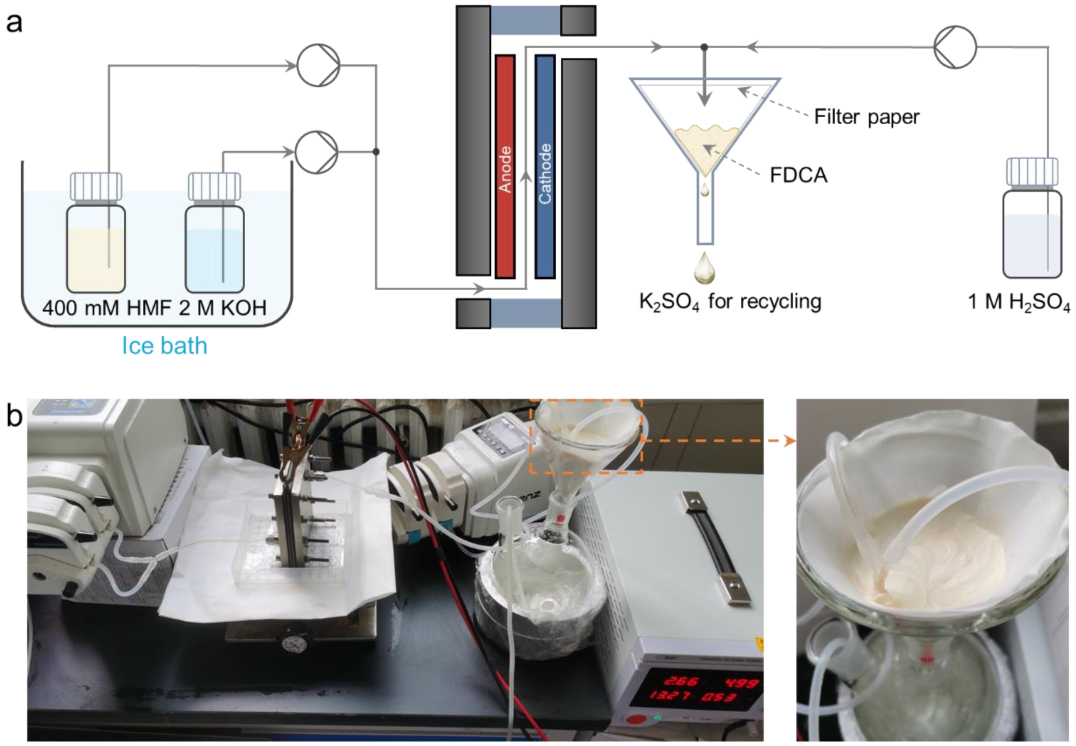

Supplementary Figure 30. Setting-up for continuous HMF oxidation in SPCFR. a Schematic illustration of the electrocatalytic HMF oxidation in SFCFR. **b** Photograph of set-up.

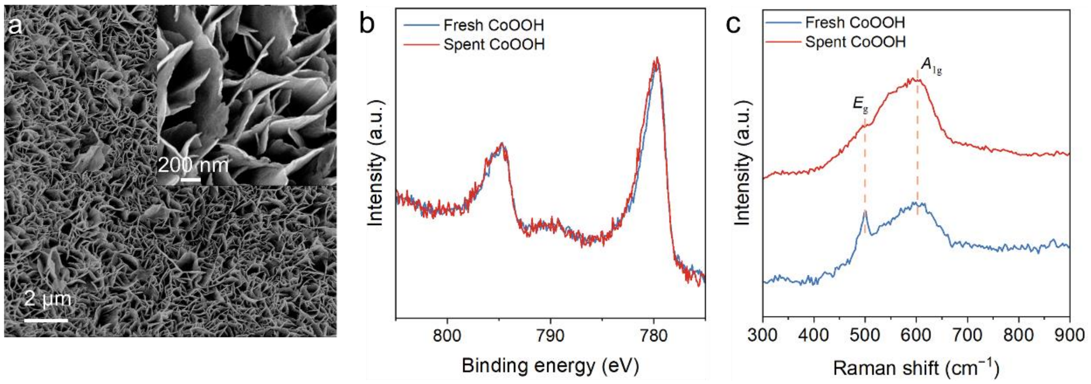

Supplementary Figure 31. Characterizations of spent CoOOH/NF anode. **a** SEM images of spent CoOOH/NF. Inset shows the enlarged region. **b** The Co 2*p* XPS spectra and (**c**) Raman spectra of fresh and spent CoOOH.

After stability test (Fig. 3f), the spent CoOOH/NF anode was characterized by SEM, XPS, and Raman techniques. As shown in Supplementary Fig. 31a, the structure of CoOOH/NF was maintained, showing similar nano-array structure to that of the fresh

catalyst (Supplementary Fig. 4a). In addition, Co 2*p* XPS spectra and Raman spectra of the spent CoOOH were well consistent with that of the fresh one (Supplementary Fig. 31b, c), confirming the stability of CoOOH/NF anode.

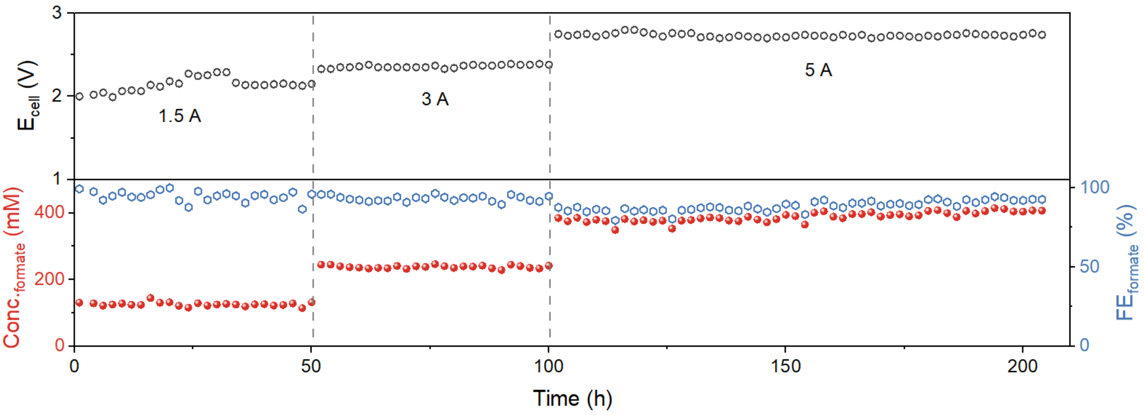

Supplementary Figure 32. Continuous operation of single module SPCFR for GOR at various currents. Reaction conditions: 3.8 mL min^{-1}, 1 M KOH with 150 mM glucose.

We evaluatd the stability of the developed single-module SPCFR (30 cm^2) for continuous GOR in long-term reactions. In long-term (>200 h) test at different currents (1.5, 3, and 5 A), no obvious degradation regarding catalytic performance was observed. Particularly, the cell voltage (~2.73 V), FE (~91%), and concentration of the generated formate (~400 mM) at 5 A (corresponding to 166.7 mA cm^{-2}) remain stable, demonstrating the operational stability of the SPCFR.

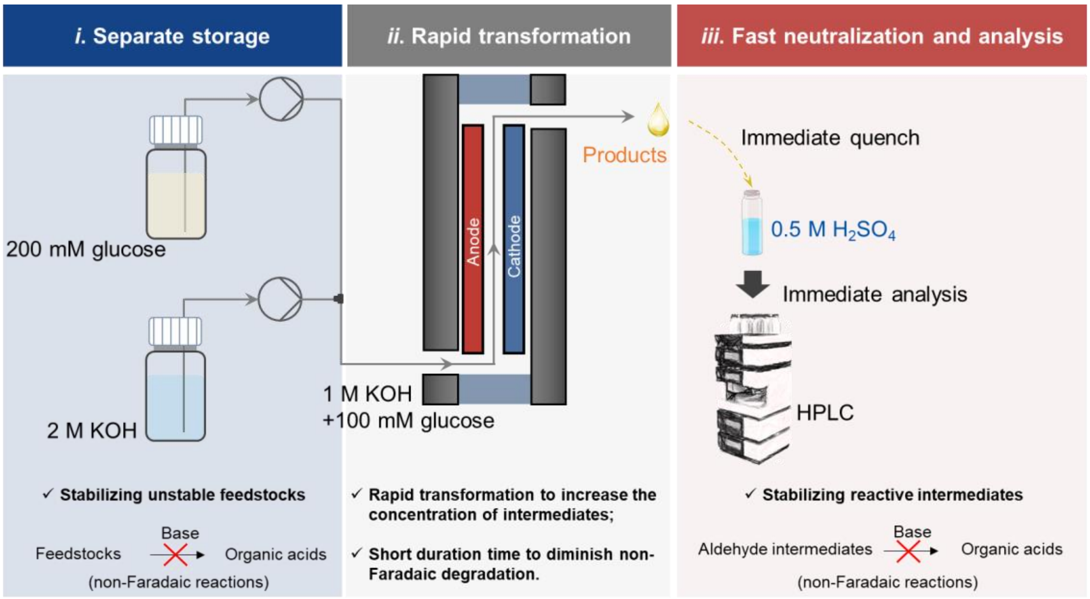

Supplementary Figure 33. Scheme of probing unstable intermediates during electrocatalytic glucose oxidation. The system is divided into three parts (from left to right):

i Separate storage: The feedstock solutions of electrolyte (2 M KOH) and glucose (200 mM) were separately stored to suppress base-catalyzed non-Faradaic degradation to generate organic acids.

ii Rapid transformation: A single-module SPCFR (Supplementary Fig. 19) was employed for electrocatalytic GOR to formate using a mixed electrolyte composing of 1 M KOH and 100 mM glucose at a flow rate of 11.4 mL min^{-1}. The SPCFR system enables rapid transformation of glucose, affording detectable reaction intermediates for subsequent HPLC analysis. The SPCFR system also shortens the duration time of glucose and intermediates in the reactor, hence diminishing non-Faradaic degradation. In addition, the SPCFR can be operated at different currents (0−7 A) to detect the variation of possible reaction intermediates.

iii Fast neutralization and analysis: The electrolyte at the outlet of SPCFR was collected and immediately quenched (that is, neutralized) by a dilute acid (0.5 M H_2SO_4) to stabilize the reactive intermediates. The products were then immediately analyzed by HPLC. By doing this way, the reactive intermediates (such as aldehyde intermediates) can be stabilized without being transformed into organic acids by base-catalyzed degradation.

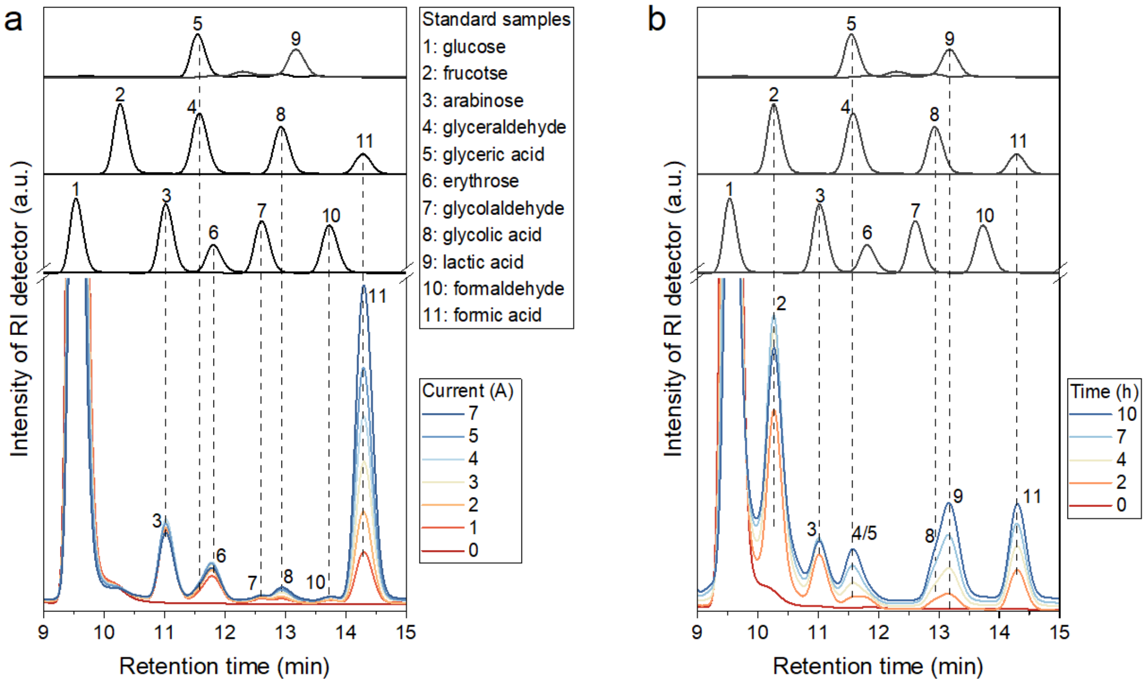

Supplementary Figure 34. HPLC chromatogram of electrolyte and standard samples. a Electrolyte of SPCFR system. **b** Electrolyte of batch reactor.

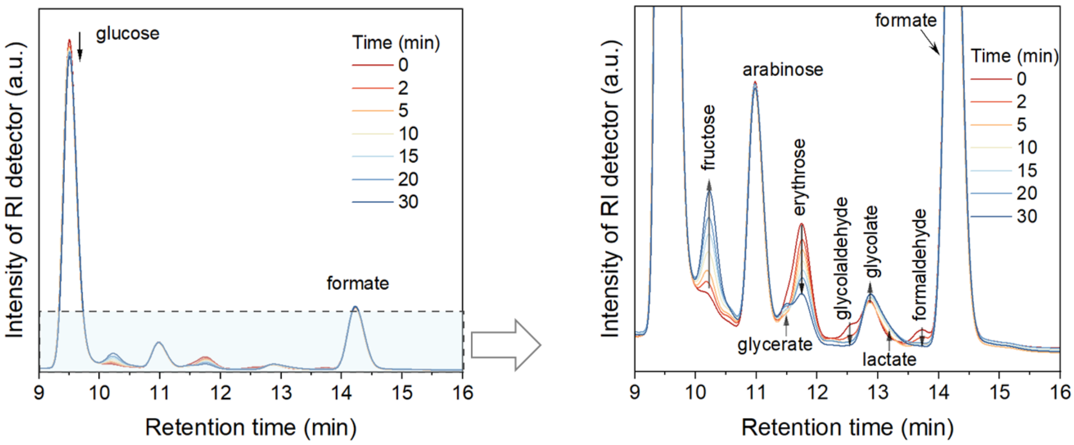

Supplementary Figure 35. Evolution of HPLC chromatograms of the electrolyte of SPCFR collected at 5 A as a function of interval time for acid neutralization.

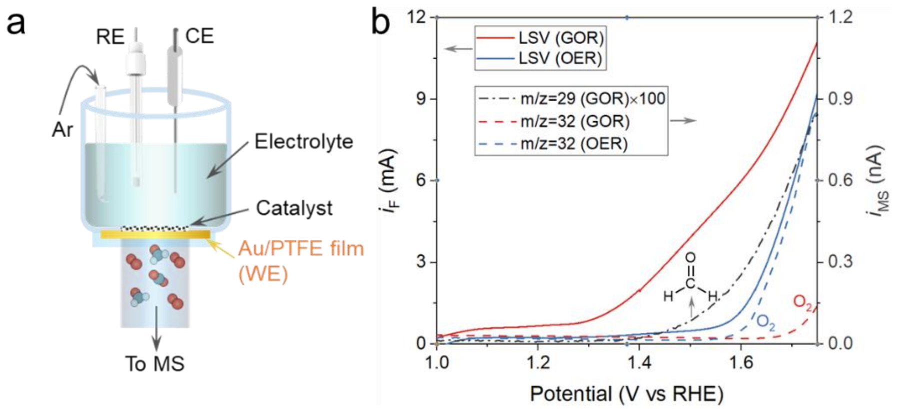

Supplementary Figure 36. On-line differential electrochemical mass spectrometry (DEMS) using a batch type reactor. a Scheme of experimental set-up. **b** LSV curves and corresponding MS signals of OER and GOR in DEMS experiments.

We employed on-line DEMS to detect possible formaldehyde intermediate during glucose oxidation reaction (GOR) in batch reactor. As shown in Supplementary Figure 36, the presence of glucose in the electrolyte induces obvious faradaic current (i_F) since 1.3 V, while the signal from O_2 was only observed upon 1.65 V vs RHE. Notably, the mass signal (i_{MS}) of formaldehyde (m/z = 29) was detected in the potential window of GOR, indicating this reaction may proceed via route I. Unfortunately, aldose intermediates cannot be detected by DEMS because of their high boiling points.

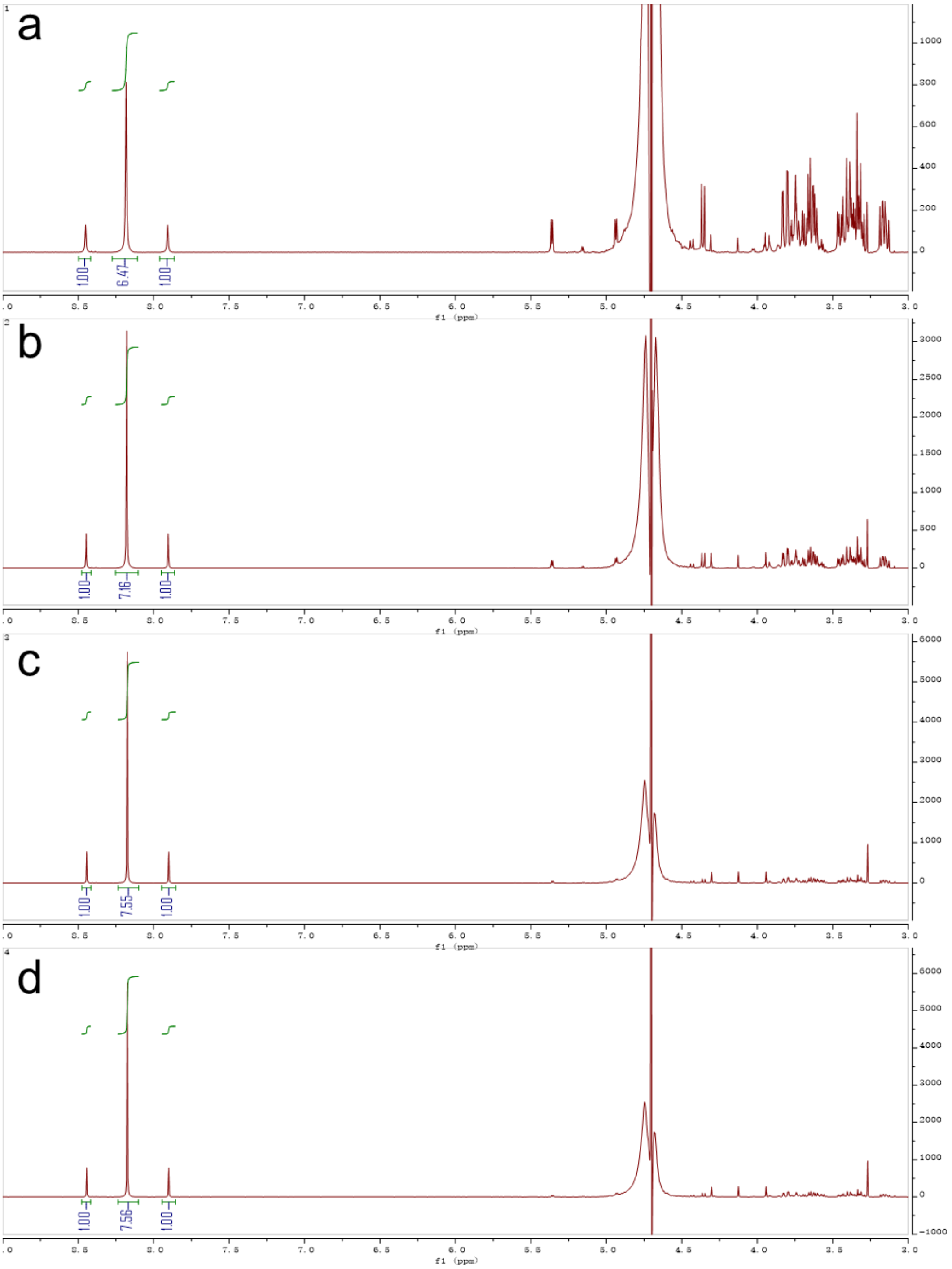

Supplementary Figure 37. ^{1}H NMR spectra results. ^{1}H NMR spectra of the electrolyte of $^{13}C1$-labeled glucose at different charge of theoretical value, including **a** 16.7%, **b** 33.3%, **c** 50.0%, **d** 83.3% of theoretical charge.

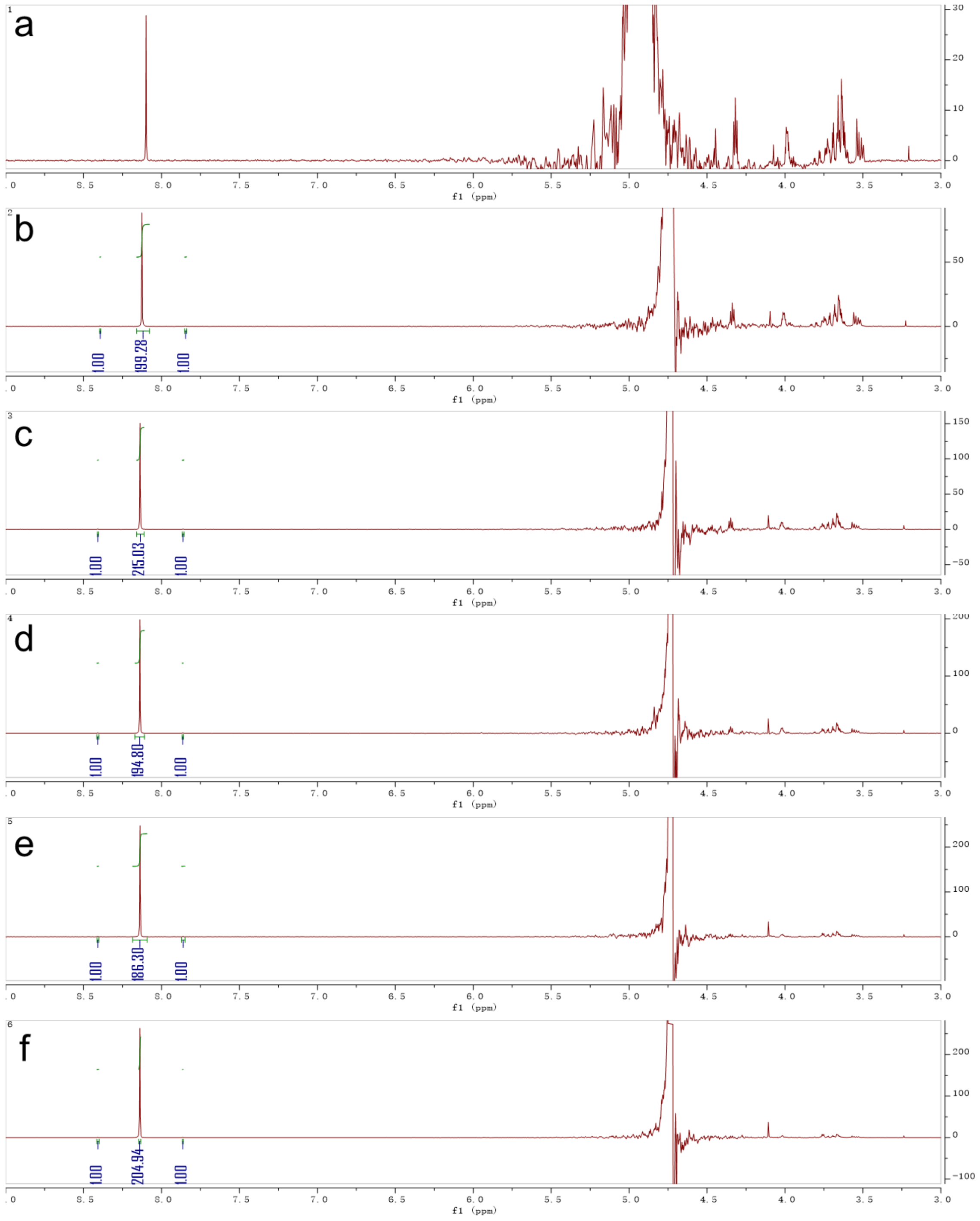

Supplementary Figure 38. ^{1}H NMR spectra results. ^{1}H NMR spectra of the electrolyte of $^{13}C1$-labeled gluconate at different charge of theoretical value, including **a** 16.7%, **b** 34.2%, **c** 49.2%, **d** 64.1%, **e** 82.0%, **f** 95.6% of theoretical charge.

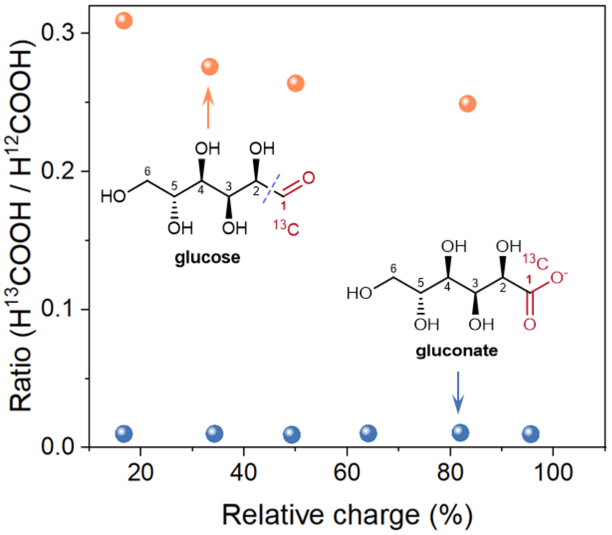

Supplementary Figure 39. ^{13}C1 isotope experiments results. Ratio of $H^{13}COOH/H^{12}COOH$ in the electrolyte of ^{13}C1 isotope experiments.

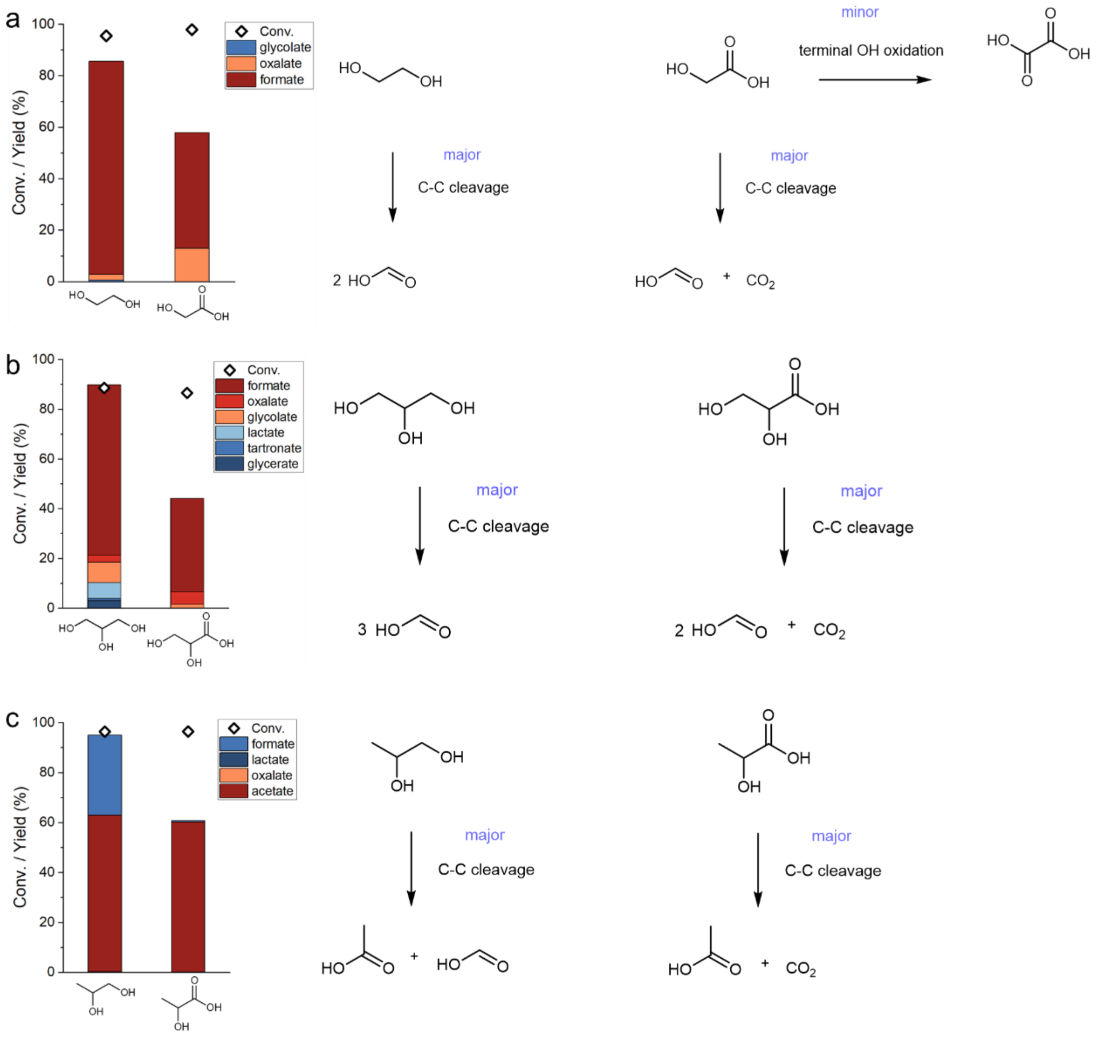

Supplementary Figure 40. Eliminating route II for C−C bond cleavage to formate.

a Conversion and product yield in electrooxidation of EG and glycolic acid. **b** Conversion and product yield in electrooxidation of glycerol and glyceric acid. **c** Conversion and product yield in electrooxidation of 1,2-propylene glycol and lactic acid. Reaction conditions: 100 mM substrate in 50 mL 1 M KOH electrolyte, CoOOH/NF and Pt foil as anode and cathode, respectively, at 1.5 V vs RHE.

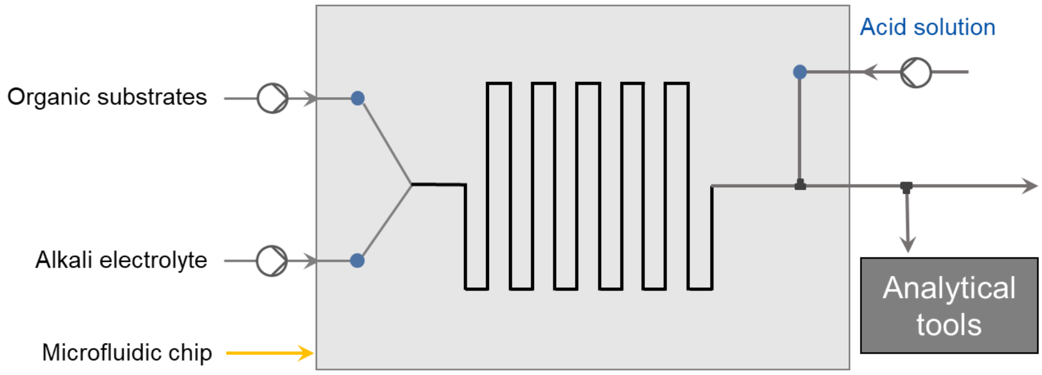

Supplementary Figure 41. Concept of microfluidic cell design. Concept of microfluidic cell design for determining unstable intermediates or isotope labelling experiments during electrolysis in the future.

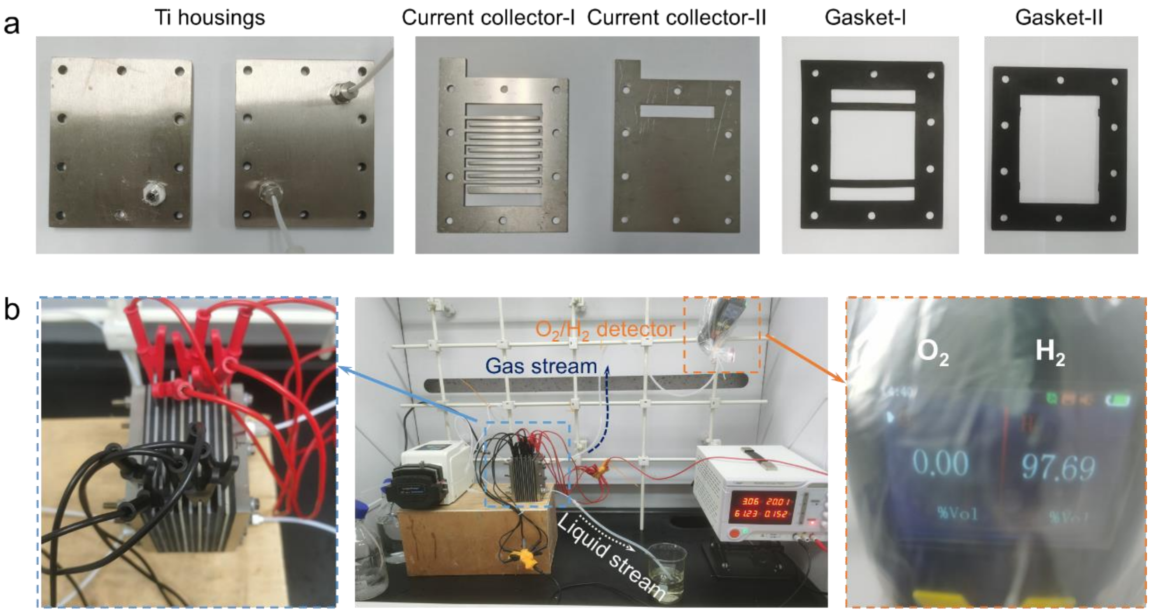

Supplementary Figure 42. Set-up of stacked SPCFR system. **a** Photographs of components in the SPCFR. **b** Photograph of stacked SPCFR system.

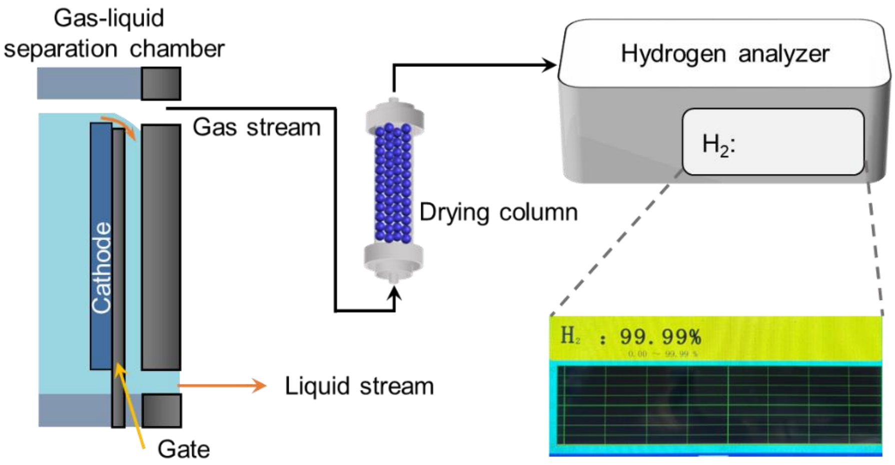

Supplementary Figure 43. Scheme of gas-liquid separation chamber in the stacked SPCFR electrolyzer. The generated gas from the electrolyzer passed through a drying column filling with desiccant silica gel before it entered to a H_2 analyzer.

There are two outlets in the gas-liquid separation chamber of the stacked SPCFR, with the upper one for gas stream and the lower one for liquid stream. In addition, there is a gate before the outlets. The gas (that is H_2) is flowing through the gate and exiting from the upper outlet because of its low density. The liquid (that is formate solution) is flowing through the gate, falling down and exiting from the lower outlet. This design for separating gas and liquid is inspired by a previous work by Kato and colleagues[35], in which a reactor was designed for CO_2 electroreduction. The generated gas from the electrolyzer passed through a drying column filling with desiccant silica gel before it entered to a H_2 analyzer. After drying treatment, H_2 purity was measured to be 99.99%, higher than that without drying (97.69%; Supplementary Fig. 42b).

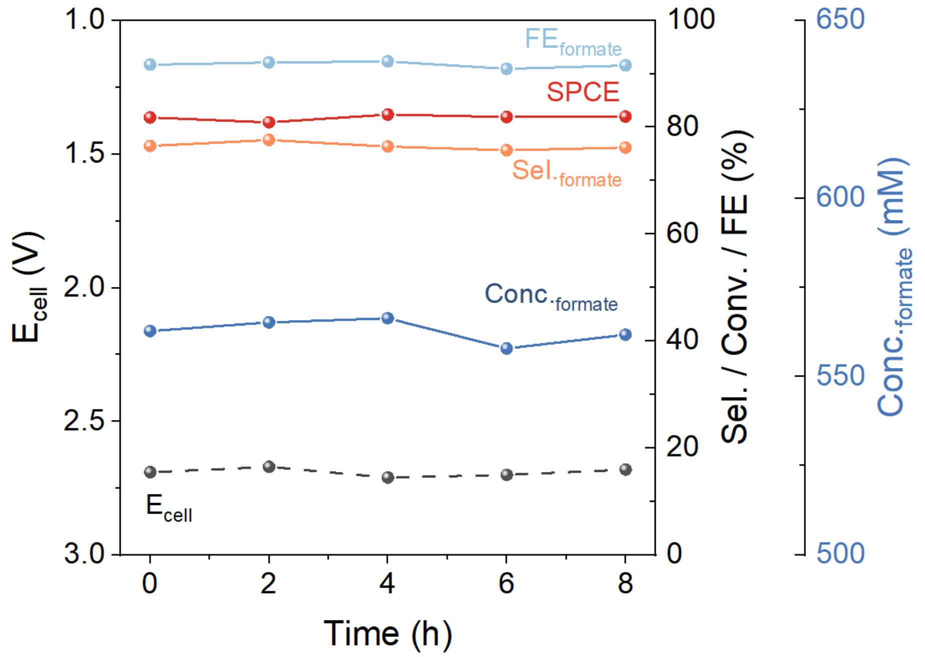

Supplementary Figure 44. Continuous operation of stacked SPCFR at 15 A for GOR. Conditions: 7.6 mL min^{-1} of feed stock solution composed of 1 M KOH and 150 mM glucose at 4 °C.

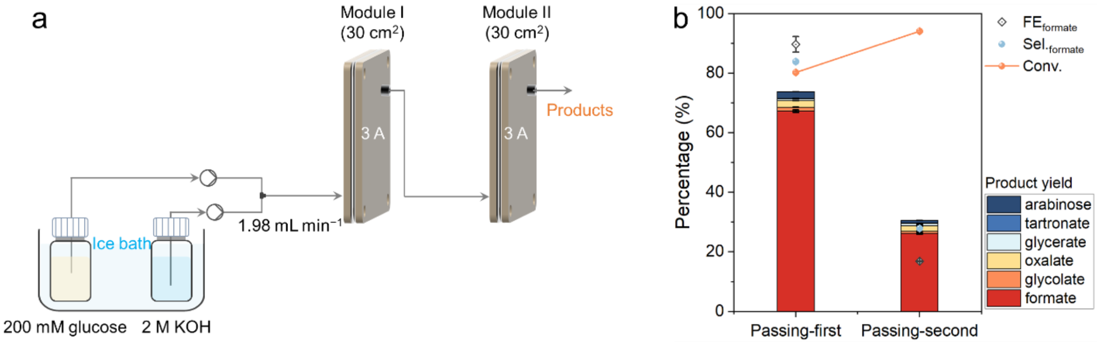

Supplementary Figure 45. Catalytic performances of glucose electrooxidation in a two-tandem-module reaction system. a Configurations of reaction system consisting of two tandem modules. **b** Corresponding catalytic results after the electrolyte passed through the first and second module. Error bars correspond to the standard deviation of three measurements.

Under an optimized conditions (i.e., current of 3 A, flow rate of 1.98 mL min^{-1}, obtained from Supplementary Fig. 20), good catalytic results (80.2% of conversion, 83.8% of formate selectivity, and 89.6% of formate FE) were obtained after the electrolyte passed through the first module (Supplementary Fig. 45b). After the liquid

stream passed through the second module, higher conversion efficiency (94%) was achieved, but formate selectivity (27.8%) and FE (16.8%) decreased. This can be explained by the overoxidation of formate to carbonate, as evidenced by the formation of large number of CO_2 when the electrolyte was acidified (pretreatment for HPLC analysis).

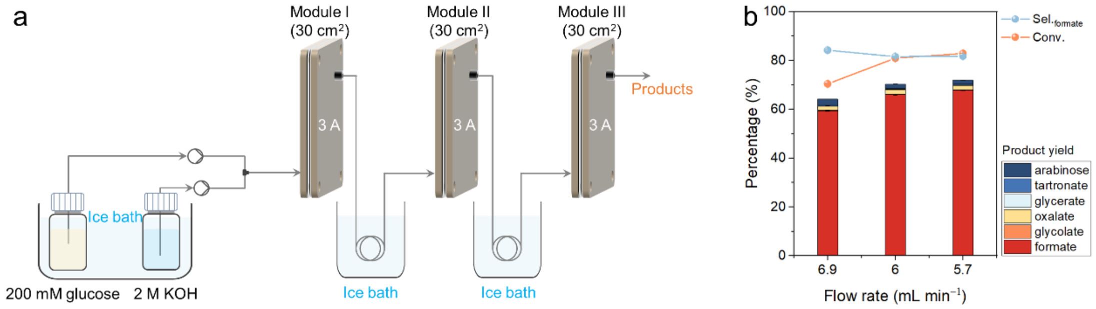

Supplementary Figure 46. Catalytic performances of glucose electrooxidation in a three-tandem-module reaction system. a Scheme and configurations of the three-tandem-module reaction system. **b** Catalytic performances of the reaction system as a function of flow rate. Error bars correspond to the standard deviation of three measurements.

The reaction was operated at a total current of 9 A (3A for each module, Supplementary Fig. 46a). We obtained glucose conversion of 80.9%, formate yield of 66.0% and selectivity of 81.6%, at a flow rate of 6 mL min^{-1} (Supplementary Fig. 46b). In contrast, in a single reactor, we obtained similar catalytic performance (glucose conversion of 80.2%, formate yield of 67.2% and selectivity of 83.8%; reactor system at condition of 3 A) but with much lower flow rate (1.98 mL min^{-1}). These results show high potential of linearly scaling up of tandem modules for biomass valorization.

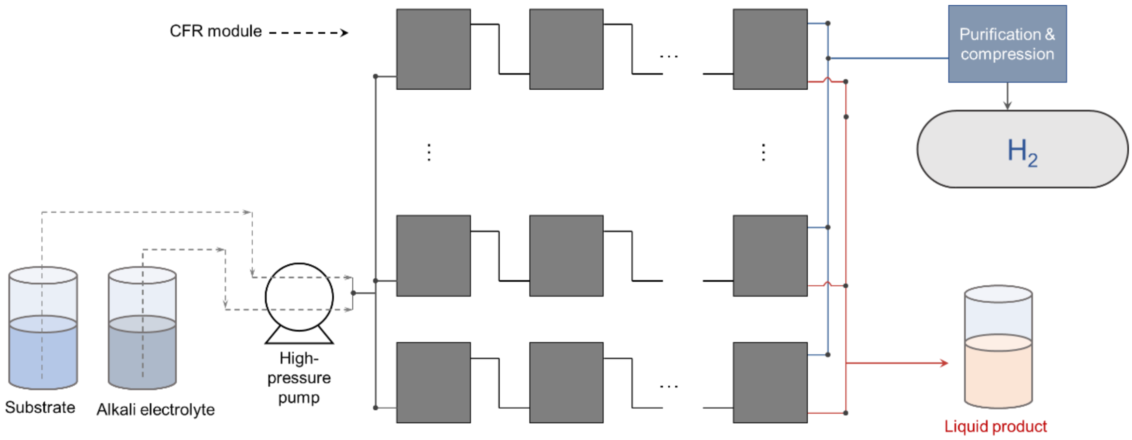

Supplementary Figure 47. Proposed modular scaling-up. Proposed modular scaling-up of process for upgrading biomass-derivatives to higher-value oxygenates and H_2.

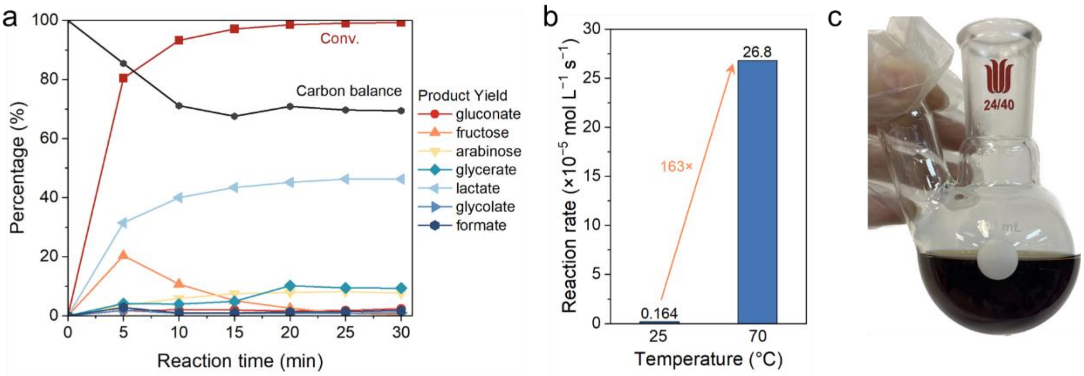

Supplementary Figure 48. Influence of high temperature on glucose degradation. **a** Glucose (100 mM) degradation and products formation as a function of reaction time at 70 °C in 1 M KOH solution. **b** Comparation of glucose degradation rate in 1 M KOH solution at room temperature (25 °C) and 70 °C. **c** Photograph of the reaction solution after 30 min.

To estimate the temperature effect on non-Faradaic degradation, we conducted glucose degradation reaction in 1 M KOH at 70 °C without applying electrolysis. As shown in Supplementary Fig. 48a, more than 80% of glucose was consumed within 5 min, corresponding to a reaction rate of 2.68×10^{-4} mol L^{-1} s^{-1}. Finally, glucose was completely degraded within 30 min, mainly delivering lactate (46.3% yield) and humins as dark-brown pigments (Supplementary Fig. 48b, c). In contrast, at room temperature (25 °C), only 41.4% of glucose was consumed after 7-h reaction

(Supplementary Fig. 12a), giving a much lower degradation rate (1.64×10^{-6} mol L^{-1} s^{-1}). These results suggest that high temperature may dramatically increase non-Faradaic degradation rate by two orders of magnitude (specifically, 163 times shown in the Supplementary Fig. 48b), and result in low formate selectivity (<65%) for reactions without temperature management (entries 4–6 of Supplementary Table 5). Therefore, we recommended to manage the temperature of electrolyte to suppress degradation of unstable biomass derivatives during electrolysis, thereby improving the selectivity of targeted product via electrolytic transformation.

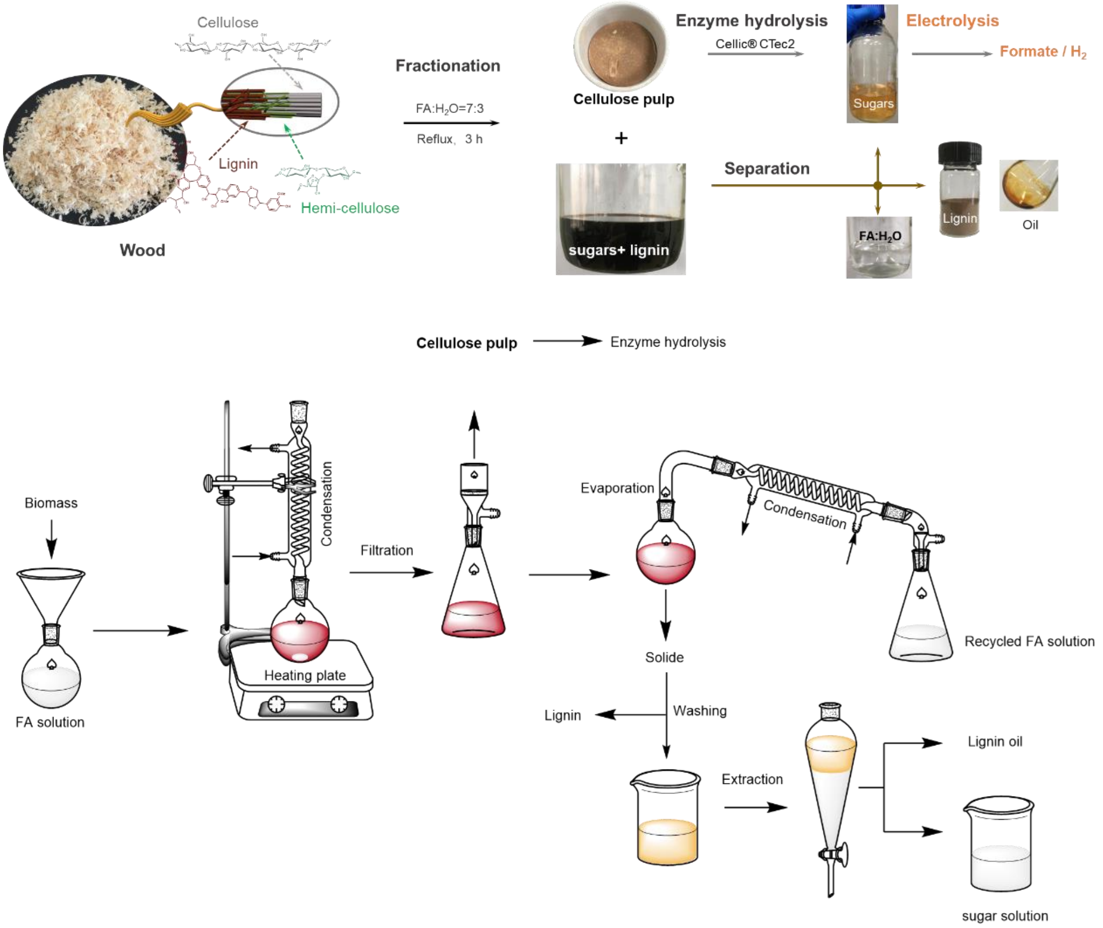

Supplementary Figure 49. Process for preparation of lignocellulosic sugars and lignin products. The details are shown in the "Fractionation of lignocellulosic biomass" section in Methods of the main text.

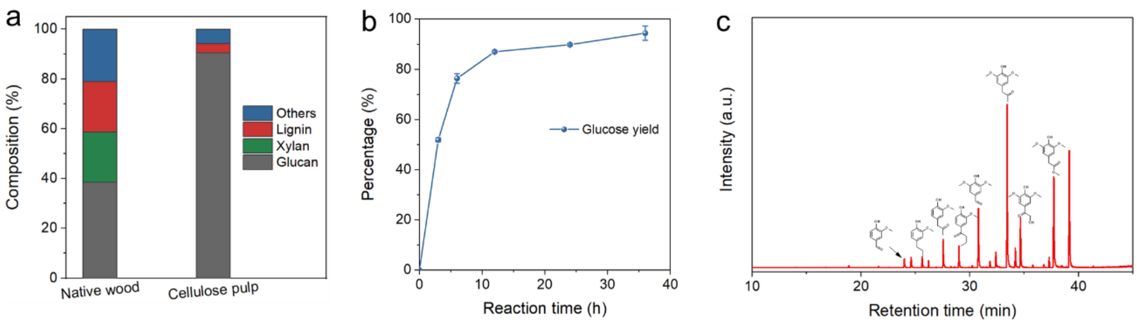

Supplementary Figure 50. Fractionation of birch word. a Composition of native birch wood and obtained cellulose pulp. **b** Yield of glucose via enzyme-catalyzed cellulose hydrolysis as a function of reaction time. Error bars correspond to the standard deviation of three measurements. **c** Total ion chromatogram of obtained lignin oil.

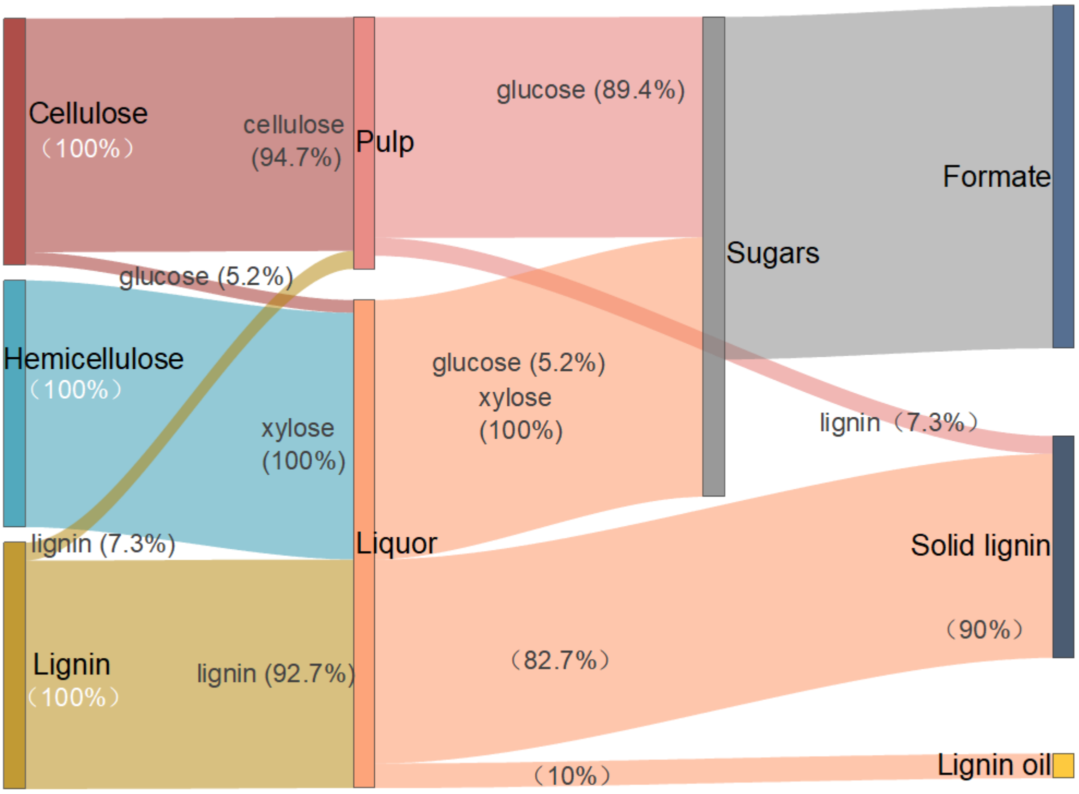

Supplementary Figure 51. Mass flow analysis of birch wood conversion. The birch wood was firstly fractionated into cellulose-rich pulp and liquor fraction using aqueous formic solution (see details in method section). The obtained pulp was transformed into glucose by enzymatic hydrolysis. The obtained liquor containing dissolved lignin and hydrolyzed hemicellulose (i.e., xylose) was distilled to recover formic acid and precipitate lignin. After the distillation, the residual solid was extracted by ethyl acetate and water to obtain lignin oil and sugar solution, respectively. Finally, the combined

sugars were converted to formate by electrooxidation.

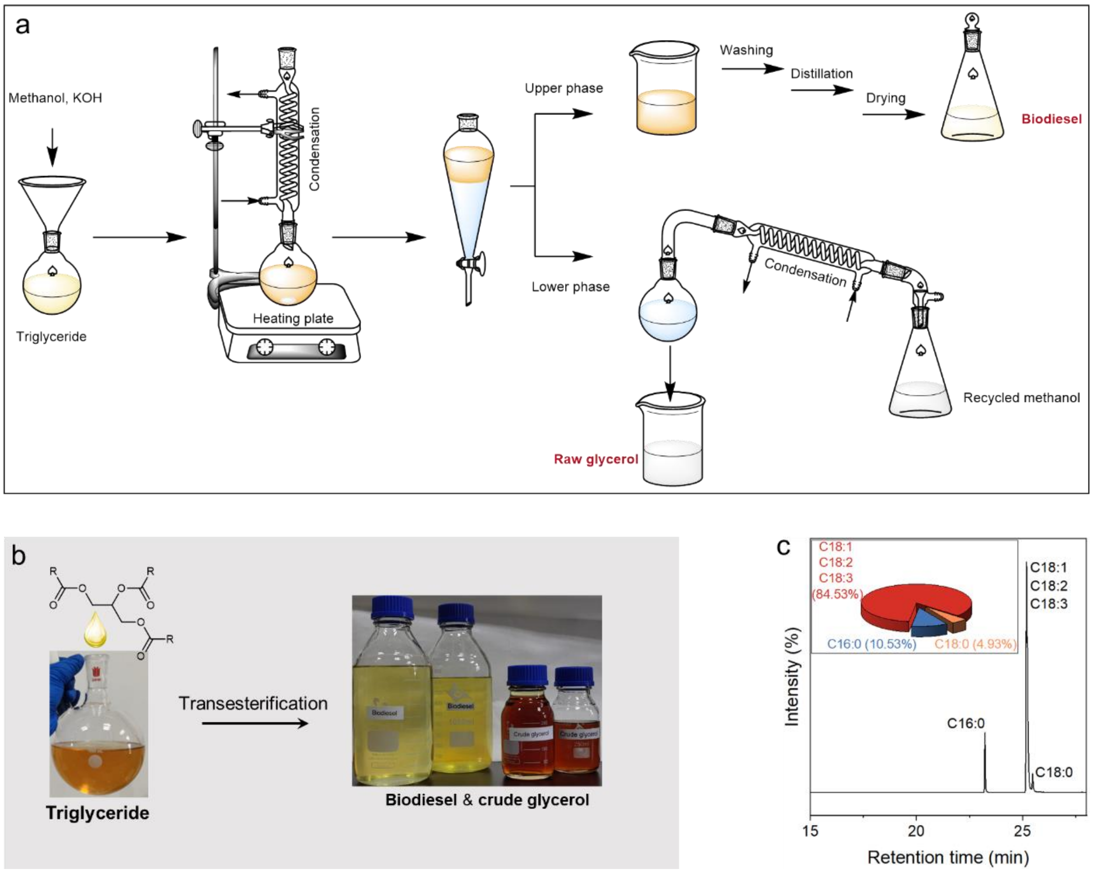

Supplementary Figure 52. Biodiesel and glycerol production. a Illustration of biodiesel and crude glycerol preparation. **b** Photograph of feedstock and products. **c** Total ion chromatogram of prepared biodiesel.

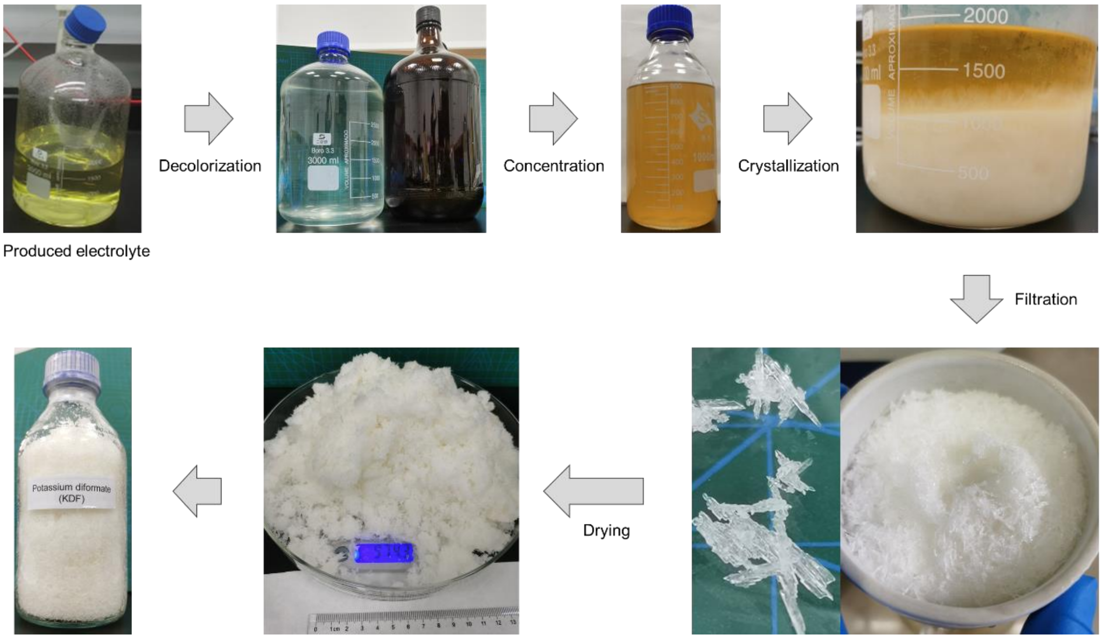

Supplementary Figure 53. Procedures for potassium diformate (KDF) synthesis. Procedures for potassium diformate (KDF) synthesis from the produced electrolyte via electrooxidation of pure glucose, lignocellulosic sugars, and crude glycerol. After electrooxidation, the generated formate (in potassium salt form) was acidified by formic acid to produce KDF via decolorization, concentration, crystallization, filtration and drying processes.

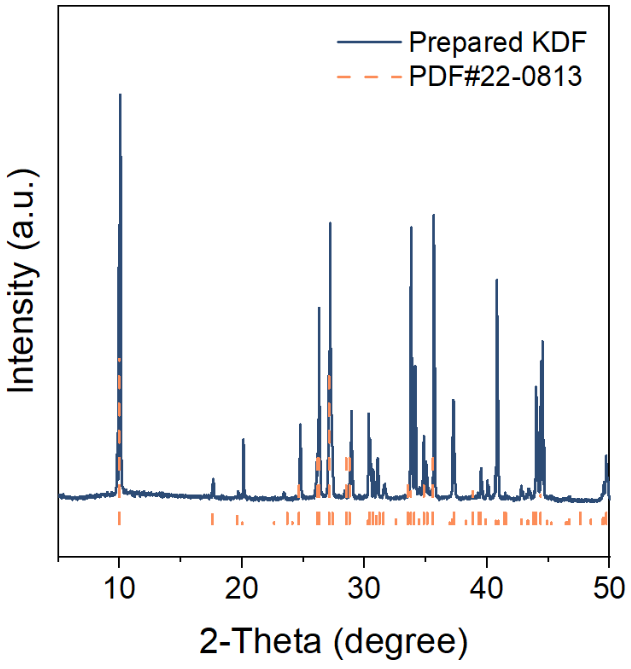

Supplementary Figure 54. XRD pattern. XRD pattern of as-prepared KDF.

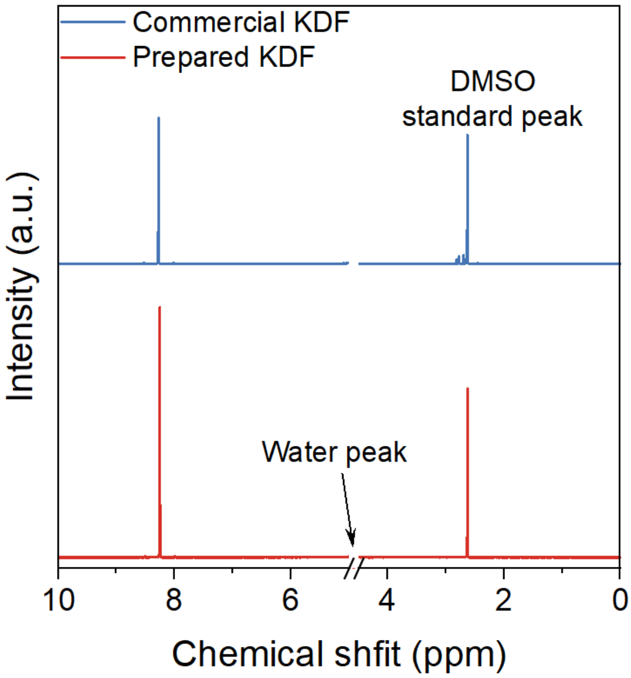

Supplementary Figure 55. ^1H NMR spectra of commercial and prepared-KDF. 10 mg mL^{-1} of KDF dissolved in water with DMSO as internal standard.

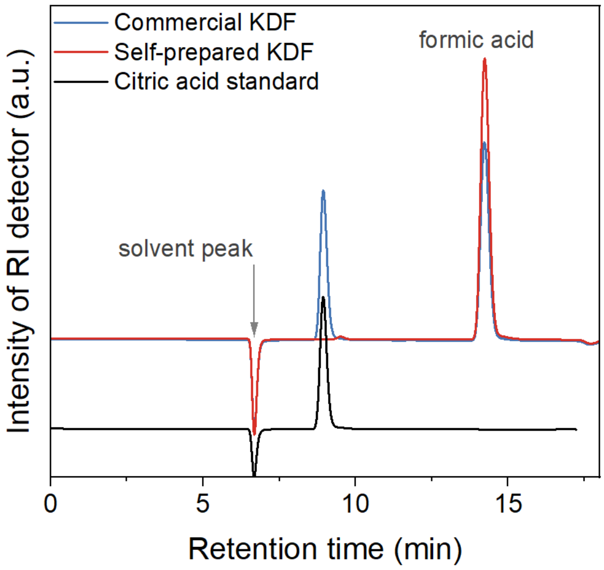

Supplementary Figure 56. HPLC chromatogram results. HPLC chromatogram of self-prepared KDF (>98% purity) and commercial KDF (Shandong Luxi Animal Medicine Share Co., Ltd., 85% purity). The commercial KDF contains silica and citric acid.

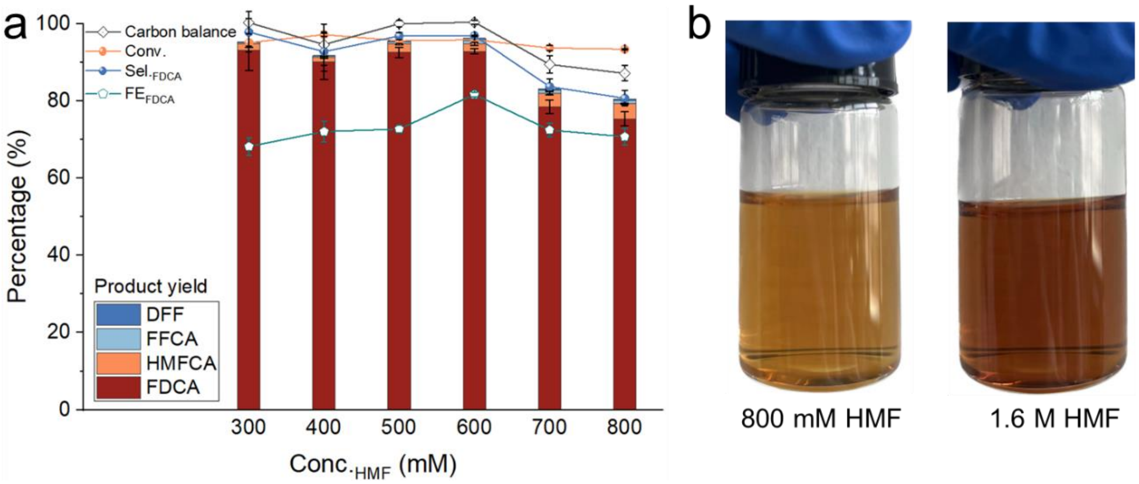

Supplementary Figure 57. Conversion of high-concentration HMF. a Optimization of HMF concentration for FDCA production in the stacked SPCFR at 15 A. Error bars correspond to the standard deviation of three measurements. **b** Photographs of HMF aqueous solution at high concentration of 800 mM (left) and 1.6 M (right).

Supplementary Tables 1−7

Supplementary Table 1. Summary of current publications on electrocatalytic HMF oxidation to FDCA

Year	Catalyst	Reactor	Electrolyte composition[a]	Electrolyte volume (mL)	Conv. (%)	Sel.$_{FDCA}$ (%)	Conc. $_{FDCA}$ (mM)	Ref.
1991	NiOOH	H-cell	1 M NaOH+5 mM HMF	NG	NG	71 (yield)	3.55	36
2014	Pd_1Au_2/C	Circulated MEA	0.1 M KOH+20 mM HMF	25	100	83	16.6	37
2015	Au electrode	Undivided cell	0.5 M borate buffer+7.5 mM TEMPO+5 mM HMF	≈15	100	99.5	4.98	38
	Carbon-felt			≈15	100	98.8	4.94	
2016	Ni_2P NPA/NF	H-cell	1 M KOH+10 mM HMF	10	≈100	≈99	≈9.9	39
2016	Co-P/CF	H-cell	1 M KOH+50 mM HMF	≈20	100	90	45	40
2016	Ni_3S_2/NF	H-cell	1 M KOH+10 mM HMF	10	100	98	9.8	41

2017	hp-Ni	NG	1 M KOH+10 mM HMF	10	100	≈95	≈9.5	[42]
2018	Ni_xB	Circulated MEA	1 M KOH+10 mM HMF	10	100	98.5	9.85	[43]
2018	Nanocrystalline Cu	H-cell	0.1 M KOH+5 mM HMF	14	99.9	96.5	4.82	[44]
2018	NiFe-LDH	H-cell	1 M KOH+10 mM HMF	30	99	99	9.8	[45]
2018	MnO_x	H-cell	pH 1 H_2SO_4+20 mM HMF 60 °C	15	99.9	53.8	10.76	[46]
2018	Ni/NiOOH	Flow cell	0.1 M Na_2SO_4+1189 mM HMF (Continuously adding of NaOH solution)	NG	100	82	350	[47]
2018	Ni foam	H-cell	1 M NaOH+10 mM HMF	15	100	83	8.3	[48]
2018	NiOOH	Undivided cell	0.1 M KOH+5 mM HMF	14	99.8	96.2	4.8	[49]
2019	VN	Circulated MEA	1 M KOH+10 mM HMF	100	92	≈97.8	≈9	[50]

2019	$BiVO_4$/CoPi	Undivided cell	0.5 M borate buffer+5 mM TEMPO+1 mM HMF	20	≈100	88	0.88	[51]
2019	Carbon-felt	H-cell	0.5 M sodium borate buffer (pH 9.2)+1 mM ACT+10 mM HMF	12.5	100	98	9.8	[52]
2019	Ni_3N@C	H-cell	1 M KOH+10 mM HMF	≈30	≈100	≈98	≈9.8	[53]
2019	NiB_x	H-cell	1 M KOH+10 mM HMF	40	100	99.8	9.98	[54]
2019	$NiCo_2O_4$	H-cell	1 M KOH+5 mM HMF	≈10	99.6	90.8	4.52	[55]
2019	$CuNi(OH)_2$/C	H-cell	1 M KOH+5 mM HMF	30	98.8	94.4	4.66	[56]
2019	BNC-2	H-cell	0.1 M NaOH+5 mM HMF	NG	71	80.3	2.85	[57]

2019	NiP-Al_2O_3/NF	H-cell	1 M KOH+10 mM HMF	30	98.2	99.6	9.78	[58]
2019	3D N-MoO_2/Ni_3S_2 NF	NG	1 M KOH+10 mM HMF	≈50	≈94.4	≈93.7	≈8.8	[59]
2020	β-$Co_{0.1}Ni_{0.9}(OH)_2$/NF	H-cell	1 M KOH+50 mM HMF	25	≈100	≈96	48	[60]
2020	AuNi	H-cell	1 M KOH+10 mM HMF	≈15	≈100	≈90	9	[61]
2020	NiO-Co_3O_4/NF	H-cell	1 M KOH+10 mM HMF	≈10	≈100	≈98	≈9.8	[62]
2020	MoO_2-FeP@C	H-cell	1 M KOH+10 mM HMF	20	99.4	98.6	9.8	[63]
2020	$CuCo_2O_4$	H-cell	1 M KOH+10 mM HMF	≈10	≈100	93.7	9.37	[64]
2020	CoO-$CoSe_2$	H-cell	1 M KOH+10 mM HMF	15	100	99	9.9	[65]
2020	NiSe@NiO_x	H-cell	1 M KOH+10 mM HMF	10	98	98	9.6	[66]

2020	NiCoFe-LDHs	H-cell	1 M NaOH+10 mM HMF	35	95.5	88.9	8.49	[67]
2020	$(AuPd)_7$	NG	1 M KOH+5 mM HMF	10	49.3	22.5	0.56	[68]
2020	NiCoBDC-NF	H-cell	0.1 M KOH+10 mM HMF	20	NG	NG	9.9	[69]
2020	TiO_x@MnO_x	Undivided cell	0.1 M $HClO_4$+20 mM HMF	2	100	58.15	11.63	[70]
2020	NiO-CMK-1	H-cell	0.2 M KOH+20 mM HMF	2.5	65	79	10.27	[71]
2020	Co–P_DES	Undivided cell	0.5 M $NaHCO_3$+5 mM HMF	≈20	99	86.16	4.265	[72]
2020	NiB_x–$P_{0.07}$	H-cell	0.1 M KOH+10 mM HMF	6	>99	91.52	9.06	[73]

2020	Ni NPs	NG	0.1 M KOH+10 mM HMF	NG	NG	NG	NG	[74]
2020	$FeSn_2$	H-cell	1 M KOH+20 mM HMF	NG	NG	NG	NG	[75]
2020	Cu_xS@NiCo LDH	NG	1 M KOH+10 mM HMF	10	≈100	≈99	≈9.9	[76]
2020	t-NiCo-MOF	NG	1 M KOH+10 mM HMF	10	≈100	≈100	10	[77]
2020	TpBpy-Ni@FTO	NG	0.1 M $LiClO_4$+0.5 mM HMF	10	96	60.4	0.29	[78]
2021	NiFe-LDH/NF	Circulated MEA	0.1 M KOH+10 mM HMF	100	99	98	9.7	[79]
2021	Ni(NS)/CP	H-cell	0.1 M KOH+5 mM HMF	15	99.7	99.4	4.96	[80]
2021	NiCoMn-LDHs	H-cell	1 M NaOH+1 mM HMF	35	100	91.7	0.92	[81]

2021	$(FeCrCoNiCu)_3O_4$	H-cell	1 M KOH+10 mM HMF	≈10	≈100	≈97	9.7	[82]
2021	CoOOH	H-cell	1 M KOH+10 mM HMF	10	≈100	≈100	10	[83]
2021	Co_3O_4/NF	H-cell	1 M KOH+10 mM HMF	10	100	99.8	9.98	[84]
2021	Ir-Co_3O_4	H-cell	1 M KOH+10 mM HMF	≈10	≈100	≈98	≈9.8	[85]
2021	CoNiFe-MOFs	H-cell	1 M KOH+10 mM HMF	30	100	99.76	9.98	[86]
2021	CoO_xH_y	H-cell	1 M KOH+10 mM HMF	10	100	85	8.5	[87]
2021	$Ni_{0.9}Cu_{0.1}(OH)_2$	H-cell	1 M KOH+5 mM HMF	≈40	≈100	91.2	4.56	[88]
2021	WO_3/Ni	H-cell	1 M KOH+5 mM HMF	25	99.4	88.8	4.4	[89]
2021	MnCoOOH/NF	Undivided cell	1 M KOH+10 mM HMF	50	100	99.3	9.93	[90]

2021	S−Ni@C	H-cell	1 M KOH+10 mM HMF	15	99.11	96.6	9.574	[91]
2021	d-NiFe LDH/CP	H-cell	1 M KOH+10 mM HMF	10	97.35	99.4	9.68	[92]
2021	FeSi/NF	H-cell	1 M KOH+43 mM HMF	4	≈100	≈100	≈43	[93]
2021	LiMnBPO/NF	H-cell	1 M KOH+40 mM HMF	4	100	≈92.5	≈37	[94]
2021	$NiFeO_x$	Undivided cell	1 M NaOH+100 mM HMF	10	100	NG	NG	[95]
2021	CoFe@NiFe	H-cell	1 M KOH+10 mM HMF	35	100	99.8	9.98	[96]
2021	$Pt/Ni(OH)_2$	NG	1 M KOH+10 mM HMF	10	100	≈96	9.6	[97]
2021	P-HEOs/Carbon paper	NG	1 M KOH+10 mM HMF	≈10	99	98.4	9.74	[98]
2021	CoAl-LDH-NSAs	NG	1 M KOH+10 mM HMF	10	100	≈95	≈9.5	[99]
2021	NiO–OH	Undivided cell	1 M LiOH+5 mM HMF	10	100	95	4.75	[100]

2021	Carbon cloth	H-cell	0.5 M H_3BO_3+5 mM HMF+7.5 mM TEMPO	20	100	98.3	4.92	[101]
2021	Ni_3N	H-cell	1 M KOH+10 mM HMF	≈10	100	≈94	≈9.4	[102]
2021	NiCoP	NG	1 M KOH+300 mM HMF	10	99.8	99.6	298.2	[103]
		Flow cell (0.05 mL/min)	1 M KOH+300 mM HMF	NG	99.8	99.7	298.5	
2021	RuO_2/MnO_2/CNT	H-cell	0.1 M K_2SO_4+20 mM HMF (333 K)	15	100	72.1	14.42	[104]
2021	N-$NiMoO_4$	H-cell	1 M KOH+10 mM HMF	15	≈100	97	9.74	[105]
2021	NF@Mo-$Ni_{0.85}Se$	H-cell	1 M KOH+10 mM HMF	≈10	≈100	≈91	9.1	[106]
2021	$Ni(OH)_2$/NF	H-cell	1 M KOH+10 mM HMF	25	100	100	10	[107]
		H-cell	1 M KOH+50 mM HMF	25	100	100	50	
2021	$NiCo_2O_4$-CFP	H-cell	1 M KOH+10 mM HMF	31	98.4	94.3	9.28	[108]

2021	$CuMn_2O_4$	H-cell	1 M KOH+10 mM HMF	30	100	≈95	≈9.5	[109]
2021	Ni_3N-V_2O_3	NG	1 M KOH+10 mM HMF	10	97.4	98.7	9.61	[110]
2022	FeP-$NiMoP_2$/FNF	H-cell	1 M KOH+10 mM HMF	≈20	100	99.2	9.92	[111]
2022	$NiCo_2O_4$	H-cell	1 M KOH+10 mM HMF	33	99.6	99.4	9.9	[112]
2022	$Ni_{0.5}Co_{2.5}O_4$	H-cell	1 M KOH+10 mM HMF	10	100	92.4	9.24	[113]
2022	δ-MnO_2	H-cell	1 M KOH+10 mM HMF	10	100	98	9.8	[114]
2022	$Co_{0.4}$NiS@NF	H-cell	1 M KOH+10 mM HMF	10	100	99	9.9	[115]
2022	Co_3O_4/CF	H-cell	1 M KOH+10 mM HMF	5	100	93.2	9.32	[116]
2022	CoNiP	H-cell	1 M KOH+10 mM HMF	10	≈100	≈82.5	≈8.25	[117]
2022	CF-$Cu(OH)_2$	H-cell	1 M KOH+10 mM HMF	40	100	98.7	9.87	[118]

2022	Ce-CoP	NG	1 M KOH+10 mM HMF	10	100	98	9.8	[119]
2022	$Ni_3(PO_4)_2$/CP	Undivided cell	0.1 M KOH+10 mM HMF	NG	99.9	94.3	9.42	[120]
2022	NiFeP	NG	1 M KOH+10 mM HMF	15	100	99.4	9.9	[121]
2022	Y-Co–CoS_x@CN	H-cell	0.1 M KOH+5 mM HMF	20	≈100	≈96	4.8	[122]
2022	NiS_x/Ni_2P	H-cell	1 M KOH+10 mM HMF	15	≈100	98.5	9.85	[123]
2022	NiCu NTs	H-cell	1 M KOH+20 mM HMF	40	100	99	19.8	[124]
2022	Ag_n@NiCo-LDH	H-cell	1 M KOH+10 mM HMF	≈14	≈100	97.5	9.75	[125]
2022	NiOOH/$Cu(OH)_2$	H-cell	0.1 M NaOH+5 mM HMF	≈15	100	98	4.9	[126]
	$Cu(OH)_2$/NiOOH			≈15	100	99.4	4.97	
2022	CoP-CoOOH	Undivided cell	1 M KOH+10 mM HMF	≈20	99.9	99.7	9.97	[127]

			1 M KOH+50 mM HMF	≈20	99.7	99.4	49.7	
		Flow cell	1 M KOH+150 mM HMF	100	99.9	99.4	149.1	
2022	NiOOH	Undivided cell	pH 13 KOH+5 mM HMF	14	≈100	≈88	≈4.4	[128]
2022	NiP@NiC	H-cell	1 M KOH+5 mM HMF	≈30	≈95	91	4.32	[129]
2022	Cu(NSD)/CP	H-cell	0.1 M KOH+5 mM HMF	15	96	100	4.8	[130]
2022	$Ni(OH)_2$	H-cell	0.1 M KOH+5 mM HMF	NG	99.9	94.3	4.71	[131]
2022	NiO-N/C	H-cell	1 M KOH+10 mM HMF	15	99	84.8	8.4	[132]
2022	$NiMoO_4$-CNTs-CF	H-cell	1 M KOH+10 mM HMF	35	96.3	97.4	9.4	[133]

2022	NF-4	H-cell	1 M KOH+10 mM HMF	25	99.5	99	9.85	[134]
2022	Co_9S_8–Ni_3S_2	H-cell	1 M KOH+10 mM HMF	20	≈100	98.8	9.88	[135]
2022	NiCo–S	H-cell	1 M KOH+10 mM HMF	20	99.1	98	9.71	[136]
2022	Cu NPs	H-cell	0.1 M KOH+4.2 mM HMF	23	98.5	95.4	3.95	[137]
2022	NiO	Undivided cell	1 M KOH+10 mM HMF	50	93	100	9.3	[138]
2022	CuO–PdO	H-cell	1 M KOH+10 mM HMF	10	99.5	96.7	9.62	[139]
2022	NiFe	H-cell	1 M KOH+50 mM HMF	≈10	100	≈94	≈47	[140]

		Flow cell	1 M KOH+100 mM HMF	NG	≈94	≈96	≈90	
2022	Ru_1-NiO	H-cell	1 M KOH+10 mM HMF	≈20	100	≈90	≈9	[141]
2022	NiVCo-LDHs	H-cell	1 M KOH+10 mM HMF	10	≈100	99.7	9.97	[142]
2022	Co_3O_4 NSA/Ni foam	Undivided cell	0.1 M KOH+10 mM HMF	≈15	≈100	95.7	9.57	[143]
2022	NiMoP/NF	H-cell	1 M KOH+10 mM HMF	25	99.8	99.9	9.97	[144]
2022	Rbf-Ni-MOF	H-cell	1 M KOH+10 mM HMF	50	≈96	≈100	≈9.6	
2022	NiS@NOSC	H-cell	1 M KOH+10 mM HMF	≈10	100	99.6	9.96	[145]

2022	Co-Ni_xP@C	H-cell	1 M KOH+10 mM HMF	10	100	≈100	10	[146]
2022	Py-TEMPO/CNTs	NG	0.2 M Na_2CO_3+5 mM HMF	≈20	100	≈95	4.75	[147]
2022	Co_3O_4-V_O	Undivided cell	1 M KOH+5 mM HMF	≈30	100	61	3.05	[148]
2022	Ni_xSe_y–NiFe@NF	H-cell	1 M KOH+10 mM HMF	31	99.6	99.7	9.93	[149]
2022	CoP/Ni_2P-NiCoP@NC	NG	0.1 M KOH+5 mM HMF	≈20	100	98.1	4.905	[150]
2022	CoCu	NG	1 M KOH+10 mM HMF	≈10	96.2	100	9.62	[151]
2022	$Pt_{26}Ni_{74}$ NWs	H-cell	1 M KOH+10 mM HMF	≈10	≈100	≈98	≈9.8	[152]

2022	$Cu_{0.5}Co_{2.5}O_4$	Undivided cell	1 M KOH+10 mM HMF	50	≈95.5	93	≈8.9	[153]
2022	MnO_2/CF	NG	0.1 M $NaHCO_3$+50 mM HMF	5	99.8	97.2	48.5	[154]
2022	Ni-Co_2P	H-cell	1 M KOH+10 mM HMF	NG	≈100	99	9.9	[155]
2022	Vo-Co_3O_4	H-cell	1 M KOH+10 mM HMF	10	100	91.9	9.19	[156]
2022	Co-NiO/CC	Undivided cell	1 M KOH+10 mM HMF	15	97.5	97.2	9.48	[157]
2022	N-Co_3O_4/NF	NG	1 M KOH+10 mM HMF	≈50	99.5	96.8	9.64	[158]
2023	Ni foam	H-cell	1 M KOH+10 mM HMF	20	≈100	90	9	[159]
2023	Co_3O_4/NF	H-cell	1 M KOH+10 mM HMF	10	100	96.7	9.67	[160]

2023	CF-Ni MOF/Ag	H-cell	1 M KOH+10 mM HMF	≈40	99.8	97.7	9.75	[161]
2023	$NiMo_3S_4$-R	NG	1 M KOH+10 mM HMF	20	99.3	98.7	9.8	[162]
2023	Co_4N/NC@CC	H-cell	1 M KOH+10 mM HMF	10	100	98.6	9.86	[163]
2023	$Co_4N@CeO_2$	NG	1 M KOH+10 mM HMF	8	91.1	93.6	8.53	[164]
			1 M KOH+300 mM HMF	8	≈100	84.7	254.1	
2023	F-doped $NiCo_2O_4$	H-cell	1 M KOH+10 mM HMF	6	≈100	97	9.7	[165]
2023	NiFe-layer double hydroxide/FeOOH	H-cell	1 M KOH+50 mM HMF	5	≈97.3	≈97.3	≈47.4	[166]

2023	NiCoFeS-MOF	H-cell	1 M KOH+50 mM HMF	10	100	99	49.5	[167]
			0.1 M K_2CO_3+0.2 mM TEMPO+19.8 mM HMF	45	100	98	19.4	
2023	NiO	H-cell	0.1 M K_2CO_3+0.2 mM ACT+19.8 mM HMF	45	100	99	19.6	[168]
			1 M K_2CO_3+4 mM TEMPO+39.6 mM HMF	45	100	98	38.8	
2023	Co_3O_4/NF	H-cell	1 M phosphate buffer solution (PBS, 10 vol% acetonitrile)+20 mM TEMPO+10 mM HMF	10	100	99	9.9	[169]
2023	$Ni(OH)_2$/NF	H-cell	1 M KOH+25 mM HMF	NG	100	≈100	≈25	[170]
2023	$Mn_{0.2}$NiS/GF	H-cell	1 M KOH+100 mM HMF	10	99.3	98.3	97.6	[171]

		Circulated MEA	1 M KOH+100 mM HMF	100	98.3	96.3	94.7	
2023	Cr-$Ni(OH)_2$/NF-	H-cell	1 M KOH+10 mM HMF	≈20	100	98	19.6	[172]
2023	CoO–Co@C/CF	H-cell	1 M KOH+10 mM HMF	10	100	99.4	9.94	[173]
2023	NF@Co_3O_4/CeO_2	NG	1 M KOH+10 mM HMF	8	98	96.4	9.45	[174]
2023	CuOCoOOH	Undivided cell	1 M KOH+10 mM HMF	30	100	98	9.8	[175]
2023	InOOH-O_V	H-cell	1 M KOH+10 mM HMF	30	99	88.4	8.75	[176]
2023	Cu_2P_7-CoP	NG	1 M KOH+10 mM HMF	10	100	98.8	9.88	[177]

2023	CoO_xH_y	H-cell	1 M KOH+5 mM HMF	≈40	≈100	≈98	≈4.9	[178]

[a]: Electrolyte used for complete conversion of HMF.

NG: Not given.

Partly data is estimated according to the data in the figure from literatures, which is represented by ≈.

Supplementary Table 2. Electrocatalytic oxidation of biomass-derived polyhydroxy compounds and methanol to formate

Entry	Substrate	Catalyst	Reactor	Electrolyte [a]			$FE_{formate}$ (%)	Conv. (%)	$Sel._{formate}$ (%)	$Conc._{formate}$ (mM)	Ref.
				Base Conc. (M)	Substrate Conc. (mM)	Volume (mL)					
1	glucose			1	150		91.7	81.8	76.5	562.8	
2	glucose			1	100		91.5	80.4	84.0	405.2	
3	sorbitol			1	100		98.8	80.0	86.3	414.2	
4	xylose			1	120		85.1	84.5	79.2	401.5	
5	xylitol	CoOOH	Single-pass flow cell	1	120		89.3	73.5	77.4	341.3	This work
6	erythritol			1	150		93.2	76.4	79.6	364.9	
7	glycerol			1	200		82.5	75.7	91.4	415.1	
8	ethylene glycol			1	300		85.6	78.3	87.0	408.3	
9	xylose	NiOOH	Circulated Flow cell	0.2	10	NG	NG	85	48	20.4	
10	glucose	RuO_2/Ti		0.5	10	NG	NG	65	38	≈14.82	[179]

11	glucose	Cu	H-cell	0.1	40	NG	NG	NG	54.2	NG	[26]
12	glucose	NiFe-1	H-cell	1	100	5	86	NG	87	522	[180]
13	glycerol	HEA-CoNiCuMnMo	Circulated Flow cell	1	100	NG	92	NG	NG	NG	[15]
14	glycerol	Ni-Mo-N/CFC	Undivided cell	1	100	5	95	100	93	279	[23]
15	glycerol	NiCo hydroxide	H-cell	1	100	50	≈100	≈90	94.3	254.6	[181]
16	glycerol	$CoMoO_4$	H-cell	1	100	10	92.7	47.3	67.6	95.9	[12]
17	glycerol	CuCo-oxide	Undivided cell	0.1	100	NG	NG	≈36	≈85	≈91.8	[11]
18	glycerol	NC/Ni-Mo-N	Undivided cell	1	100	≈15	NG	≈85	≈95	≈242.25	[182]
19	glycerol	$CuCo_2O_4$	Undivided cell	0.1	100	2	89.1	79.7	80.6	192.71	[10]
20	glycerol	NiVRu-LDHs NAs/NF	Circulated Flow cell	1	100	NG	80	NG	NG	NG	[183]
21	glycerol	$Ni(OH)_2$	H-cell	0.1	25	14	84	NG	NG	≈15.77	[184]
22	glycerol	NiCo	H-cell	1	100	50	NG	≈89	94.3	≈84.03	[185]
23	glycerol	CuCo-oxide	Undivided cell	0.1	100	NG	NG	≈35.6	≈85.5	≈30.43	[186]
24	glycerol	Ni_3N/Co_3N-NWs	Circulated Flow cell	1	100	NG	≈96.4	NG	NG	NG	[187]
25	glycerol	Bi-Co_3O_4	H-cell	1	100	40	97.05	NG	97.01	NG	[188]
26	glycerol	$ZnFe_2O_4$	H-cell	1	500	20	NG	8.5	74.69	31.59	[9]
27	ethylene glycol	$CoNi_{0.25}P$/NF	H-cell	1	300	40	82.5	100	90.2	270.6	[6]
28	methanol	Ni_3S_2/CNTs	Undivided cell	1	1000	500	>95	NG	NG	NG	[189]

29	methanol	NiMn-LDH	H-cell	1	3000	NG	96.8	NG	NG	NG	[190]

[a]: Electrolyte used for complete conversion of substrate.

NG: Not given.

Partly data is estimated according to the data in the figure from literatures, which is represented by ≈.

Supplementary Table 3. HPLC conditions for quantification of biomass derivatives [a,b,c]

**Compound**	**Detector**	**Retention time (min)**
glucose	RI	9.5
sorbitol	RI	10.3
fructose	RI	10.2
gluconic acid	UV (210 nm)	9.2
xylose	RI	10.0
xylitol	RI	11.2
arabinose	RI	11.0
erythritol	RI	11.3
erythrose	RI	11.8
glycerol	RI	13.2
glyceric acid	UV (210 nm)	11.4
lactic acid	UV (210 nm)	12.7
glyceraldehyde	RI	11.6
tartronic acid	UV (210 nm)	8.6
ethylene glycol	RI	15.7
glycolic acid	UV (210 nm)	12.7

oxalic acid	UV (210 nm)	7.3
glycolic aldehyde	RI	12.6
formic acid	UV (210 nm)	14.1
formaldehyde	RI	13.7
HMF	UV (265 nm)	30.3
FDCA	UV (265 nm)	15.8
HMFCA	UV (265 nm)	20.5
FFCA	UV (265 nm)	21.5
DFF	UV (265 nm)	37.8

[a] According to previous protocol developed by NREL[191], HPLC was used for analyzing a portfolio of biomass derivatives.

[b] The HPLC analysis was performed on Agilent 1260 II equipped with a H^+ column (300 mm×7.8 mm, ICSep ICE-Coregel 87H3) at 60 °C using 5 mM H_2SO_4 as the mobile phase at a flow rate of 0.6 mL min^{-1}.

[c] Suitable detector and retention time for each compound are summarized.

Supplementary Table 4. Comparison of electrooxidation of biomass-derived polyhydroxy compounds at different concentration

Feed stock	Conc. (mM)	Conv. (%)	Product yield (%)								Carbon balance (%)	$Sel._{formate}$ (%)	$FE_{formate}$ (%)
			formate	glycolate	oxalate	glycerate	lactate	tartronate	fructose	gluconate			
EG	10	82.6	78.9	-	0.9	-	-	-	-	-	97.1	95.5	94.1
	100	88.8	79.5	1.4	1.9	-	-	-	-	-	94.0	89.5	93.7
Glycerol	10	85.0	72.9	1.0	2.0	0.2	-	-	-	-	91.1	85.8	84.8
	100	88.6	68.6	8.4	2.8	3.0	6.4	0.8	-	-	101.3	77.4	88.8

Erythritol	10	88.8	70.3	0.4	2.2	0.3	0.1	-	-	-	84.5	79.2	76.3
	100	78.6	38.6	6.9	1.5	4.5	12.4	0.4	-	-	85.6	49.1	82.2
Xylitol	10	84.7	72.9	1.0	2.4	1.0	0.7	-	-	-	93.1	86.0	87.6
	100	85.8	32.7	6.2	1.7	5.4	23.5	0.5	-	-	84.2	38.2	77.5
Xylose	10	85.7	69.3	0.8	2.6	0.8	0.3	-	-	-	88.0	80.9	84.7
	100	83.6	22.0	3.5	1.7	3.4	16.1	1.0	-	-	64.0	26.3	73.8
Sorbitol	10	88.4	71.5	1.1	2.6	1.1	0.9	-	-	-	88.8	80.9	86.7
	100	84.0	30.8	5.8	2.0	6.9	23.4	0.7	-	-	86.2	36.6	74.3
Glucose	10	82.6	70.8	0.6	3.7	0.9	0.2	0.1	1.7	0.5	95.8	85.7	85.7
	100	79.5	21.3	3.6	2.7	4.4	10.9	1.9	6.8	19.5	92.9	26.8	59.8

Supplementary Table 5. Preliminary optimization of parameters for GOR in stacked SPCFR system

Entry	Conditions [a]			SPCE (%)	Formate		
	Current (A)	Flow rate (mL min^{-1})	Temp. [b] (°C)		Sel. (%)	Conc. (mM)	FE (%)
1	10	3.8	RT	87.8	44.7	353.8	42.4
2	10	7.6	RT	61.4	68.4	378.1	92.9
3	10	11.4	RT	48.5	68.7	300.1	96.4
4	15	7.6	RT	88.4	65.0	509.0	86.6
5	20	7.6	RT	87.2	24.9	194.9	24.4
6	20	11.4	RT	68.5	54.8	357.0	63.8
7	15	7.6	4 °C	81.8	76.5	562.8	91.7

[a] Conditions: the active area of both NF cathode and CoOOH/NF are 270 cm^2, the feed stock solution composed of 1 M KOH and 150 mM glucose.

[b] The temperature refer to the temperature of feedstock solution.

Supplementary Table 6. Survey of the market price of KDF

Brand name	Purity (%)	Market price ($ Kg^{-1})
HUARUI	98	2.75 [a]
CDchem	96	3 [b]
Evergreen	96	5 [c]
E.fine	96	2.5 [d]
Fengda	-	3 [e]
BIOF	98	1.93 [g]
KOLOD	98	2.5 [h]
HJC	98	5 [i]
Green Mountain	90	2 [j]
ML	99	8 [k]
Average price		3.56

[a]: https://www.alibaba.com/product-detail/96-Feed-Grade-Potassium-Diformate-EU_1600511288858.html?spm=a2700.galleryofferlist.normal_offer.d_title.92b24228KQBkyV

[b]: Potassium Diformate Feed Additive High Purity Supply - Buy Potassium Diformate Feed Additive,Potassium Diformate,Potassium Diformate High Purity Product on Alibaba.com

[c]: Feed Additive Potassium Diformate Cas 20642-05-1 96% Potassium Diformate - Buy Potassium Diformate,Potassium Diformate 96%,Potassium Diformate Feed Product on Alibaba.com

[d]: https://www.alibaba.com/product-detail/Sterilization-agent-Potassium-Diformate-98-CAS_1600516866283.html?spm=a2700.galleryofferlist.normal_offer.d_image.92b24228KQBkyV

[e]: https://www.alibaba.com/product-detail/Potassium-Diformate-Mudanjiang-Fengda-Factory-Supplier_1600531204739.html?spm=a2700.galleryofferlist.normal_offer.d_image.92b24228KQBkyV

[g]: https://www.alibaba.com/product-detail/Guaranteed-Quality-Proper-Price-CAS-20642_1600378476677.html?spm=a2700.galleryofferlist.normal_offer.d_image.92b24228Dd8jpQ

[h]: https://www.alibaba.com/product-detail/factory-price-top-quality-feed-grade_10000008868447.html?spm=a2700.galleryofferlist.normal_offer.d_image.92b24228Dd8jpQ

[i]: https://www.alibaba.com/product-detail/CAS-20642-05-1-Feed-Grade_1600273952125.html?spm=a2700.galleryofferlist.normal_offer.d_image.92b24228Dd8jpQ

[j]: https://www.alibaba.com/product-detail/Potassium-Diformate-Feed-additive_1600157383770.html?spm=a2700.galleryofferlist.normal_offer.d_image.92b24228Dd8jpQ

[k]: https://www.alibaba.com/product-detail/Supply-High-Purity-Potassium-Diformate-feed_1600566101379.html?spm=a2700.galleryofferlist.normal_offer.d_image.92b24228fIZMpJ

Supplementary Table 7. Oxidation number of carbon atoms and average electron transfer for the formation of one formate

Substrate	Oxidation numbers of carbon atoms	Average electron transfer for per formate (e^- /formate)
Methanol	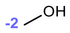	4
EG	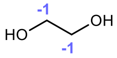	3
Glycerol	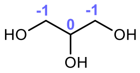	8/3
Erythritol	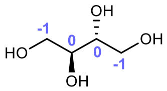	2.5
Xylitol	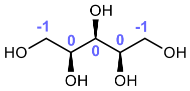	12/5
Xylose	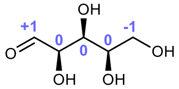	2
Sorbitol	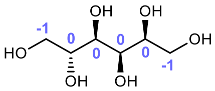	7/3
Glucose	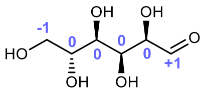	2
Glyceric acid	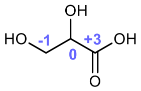	-
Glycolic acid	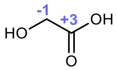	-
Formate	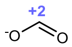	-

Supplementary References

1. Bamford CH, Bamford D, Collins JR, Wilson AH. Kinetic studies on carbohydrates in alkaline conditions - II. The kinetics of the rearrangements of glucose and fructose in alkaline solution. *Proc. R. Soc. Lond. A* **204**, 85−98 (1950).
2. Montgomery R, Ronca RA. Chemical Production of Lactic and Other Acids from Molasses. *Ind. Eng. Chem. Res.* **45**, 1136−1143 (1953).
3. Sowden JC, Kuenne DJ. Concerning the mechanism of formation of saccharinic acids. *J. Am. Chem. Soc.* **75**, 2788−2789 (1953).
4. Corbett WM, Liddle AM. The alkaline degradation of glucose and of some of its acetyl derivatives. *J. Chem. Soc.*, 531−538 (1961).
5. Zhu YQ, *et al.* Identification of Active Sites Formed on Cobalt Oxyhydroxide in Glucose Electrooxidation. *Angew. Chem. Int. Ed.* **62**, e202219048 (2023).
6. Zhou H, *et al.* Electrocatalytic upcycling of polyethylene terephthalate to commodity chemicals and H_2 fuel. *Nat. Commun.* **12**, 4679 (2021).
7. Wang J, *et al.* Electrocatalytic Valorization of Poly(ethylene terephthalate) Plastic and CO_2 for Simultaneous Production of Formic Acid. *ACS Catal.* **12**, 6722−6728 (2022).
8. Wang J, Li X, Zhang T, Chen Y, Wang T, Zhao Y. Electro-Reforming Polyethylene Terephthalate Plastic to Co-Produce Valued Chemicals and Green Hydrogen. *J. Phys. Chem. Lett.* **13**, 622−627 (2022).
9. Wan H, *et al.* Electro-Oxidation of Glycerol to High-Value-Added C1−C3 Products by Iron-Substituted Spinel Zinc Cobalt Oxides. *ACS Appl. Mater. Interfaces* **14**, 14293−14301 (2022).
10. Han XT, *et al.* Electrocatalytic Oxidation of Glycerol to Formic Acid by $CuCo_2O_4$ Spinel Oxide Nanostructure Catalysts. *ACS Catal.* **10**, 6741−6752 (2020).
11. Oh LS, *et al.* How to Change the Reaction Chemistry on Nonprecious Metal Oxide Nanostructure Materials for Electrocatalytic Oxidation of Biomass-Derived Glycerol to Renewable Chemicals. *Adv. Mater.* **35**, e2203285 (2022).
12. Yu X, *et al.* Hydrogen Evolution Linked to Selective Oxidation of Glycerol over $CoMoO_4$ —A Theoretically Predicted Catalyst. *Adv. Energy Mater.* **12**, 2103750 (2022).
13. Kim HJ, Lee J, Green SK, Huber GW, Kim WB. Selective glycerol oxidation by electrocatalytic dehydrogenation. *ChemSusChem* **7**, 1051−1056 (2014).
14. Zhou YF, Shen Y. Selective electro-oxidation of glycerol over Pd and Pt@Pd nanocubes. *Electrochem Commun* **90**, 106−110 (2018).
15. Fan L, *et al.* High Entropy Alloy Electrocatalytic Electrode toward Alkaline Glycerol Valorization Coupling with Acidic Hydrogen Production. *J. Am. Chem. Soc.* **144**, 7224−7235 (2022).
16. Santiago PVB, Lima CC, Bott-Neto JL, Fernandez PS, Angelucci CA, Souza-Garcia J. Perovskite oxides as electrocatalyst for glycerol oxidation. *J. Electroanal. Chem.* **896**, 115198 (2021).
17. Zhu Y, *et al.* Biphasic Transition Metal Nitride Electrode Promotes Nucleophile Oxidation Reaction for Practicable Hybrid Water Electrocatalysis. *Adv. Funct. Mater.* **33**, 2300547 (2023).
18. Haider MH, *et al.* Efficient green methanol synthesis from glycerol. *Nat. Chem.* **7**, 1028−1032 (2015).
19. Verma S, Lu S, Kenis PJA. Co-electrolysis of CO_2 and glycerol as a pathway to carbon chemicals with improved technoeconomics due to low electricity consumption. *Nat. Energy* **4**, 466−474 (2019).
20. Holm MS, Saravanamurugan S, Taarning E. Conversion of sugars to lactic acid derivatives using heterogeneous zeotype catalysts. *Science* **328**, 602−605 (2010).
21. Hua Zhou, Zhenhua Li, Lina Ma, Duan H. Electrocatalytic Oxidative Upgrading of Biomass Platform Chemicals: From the Aspect of Reaction Mechanism. *Chem. Commun.* **58**, 897−907 (2022).
22. Kwon Y, Lai SC, Rodriguez P, Koper MT. Electrocatalytic oxidation of alcohols on gold in alkaline media: Base or gold catalysis? *J. Am. Chem. Soc.* **133**, 6914−6917 (2011).
23. Li Y, Wei X, Chen L, Shi J, He M. Nickel-molybdenum nitride nanoplate electrocatalysts for concurrent electrolytic hydrogen and formate productions. *Nat. Commun.* **10**, 5335 (2019).
24. Filiciotto L, Balu AM, Romero AA, Angelici C, van der Waal JC, Luque R. Reconstruction of humins formation mechanism from decomposition products: A GC-MS study based on catalytic continuous flow depolymerizations. *Mol. Catal.* **479**, 110564 (2019).
25. Galkin KI, *et al.* Critical influence of 5-hydroxymethylfurfural aging and decomposition on the utility of biomass conversion in organic synthesis. *Angew. Chem. Int. Ed.* **55**, 8338−8342 (2016).
26. Moggia G, Kenis T, Daems N, Breugelmans T. Electrochemical Oxidation of d-Glucose in Alkaline Medium: Impact of Oxidation Potential and Chemical Side Reactions on the Selectivity to d-Gluconic and

d-Glucaric Acid. *Chemelectrochem* **7**, 86−95 (2020).
27. Huang J, *et al.* CoOOH Nanosheets with High Mass Activity for Water Oxidation. *Angew. Chem. Int. Ed.* **54**, 8722−8727 (2015).
28. Wang M, Liu M, Lu J, Wang F. Photo splitting of bio-polyols and sugars to methanol and syngas. *Nat. Commun.* **11**, 1083 (2020).
29. Ji N, *et al.* Direct catalytic conversion of cellulose into ethylene glycol using nickel-promoted tungsten carbide catalysts. *Angew. Chem. Int. Ed.* **47**, 8510−8513 (2008).
30. Liu W, *et al.* A durable nickel single-atom catalyst for hydrogenation reactions and cellulose valorization under harsh conditions. *Angew. Chem. Int. Ed.* **57**, 7071−7075 (2018).
31. Liao Y, *et al.* A sustainable wood biorefinery for low–carbon footprint chemicals production. *Science* **367**, 1385−1390 (2020).
32. Luterbacher JS, *et al.* Nonenzymatic sugar production from biomass using biomass-derived γ-valerolactone. *Science* **343**, 277−280 (2014).
33. You B, Liu X, Jiang N, Sun Y. A general strategy for decoupled hydrogen production from water splitting by integrating oxidative biomass valorization. *J. Am. Chem. Soc.* **138**, 13639−13646 (2016).
34. Liu WJ, *et al.* Efficient electrochemical production of glucaric acid and H_2 via glucose electrolysis. *Nat. Commun.* **11**, 265 (2020).
35. Kato N, *et al.* A large-sized cell for solar-driven CO_2 conversion with a solar-to-formate conversion efficiency of 7.2%. *Joule* **5**, 687−705 (2021).
36. Grabowski G, Lewkowski J, Skowroński R. The electrochemical oxidation of 5-hydroxymethylfurfural with the nickel oxide/hydroxide electrode. *Electrochim Acta* **36**, 1995 (1991).
37. Chadderdon DJ, *et al.* Electrocatalytic oxidation of 5-hydroxymethylfurfural to 2,5-furandicarboxylic acid on supported Au and Pd bimetallic nanoparticles. *Green Chem.* **16**, 3778−3786 (2014).
38. Cha HG, Choi KS. Combined biomass valorization and hydrogen production in a photoelectrochemical cell. *Nat. Chem.* **7**, 328−333 (2015).
39. You B, Jiang N, Liu X, Sun Y. Simultaneous H_2 Generation and Biomass Upgrading in Water by an Efficient Noble-Metal-Free Bifunctional Electrocatalyst. *Angew. Chem. Int. Ed.* **55**, 9913−9917 (2016).
40. Jiang N, You B, Boonstra R, Terrero Rodriguez IM, Sun Y. Integrating Electrocatalytic 5-Hydroxymethylfurfural Oxidation and Hydrogen Production via Co–P-Derived Electrocatalysts. *ACS Energy Lett.* **1**, 386−390 (2016).
41. You B, Liu X, Jiang N, Sun Y. A General Strategy for Decoupled Hydrogen Production from Water Splitting by Integrating Oxidative Biomass Valorization. *J. Am. Chem. Soc.* **138**, 13639−13646 (2016).
42. You B, Liu X, Liu X, Sun Y. Efficient H_2 Evolution Coupled with Oxidative Refining of Alcohols via A Hierarchically Porous Nickel Bifunctional Electrocatalyst. *ACS Catal.* **7**, 4564−4570 (2017).
43. Barwe S, *et al.* Electrocatalytic Oxidation of 5-(Hydroxymethyl)furfural Using High-Surface-Area Nickel Boride. *Angew. Chem. Int. Ed.* **57**, 11460−11464 (2018).
44. Nam D-H, Taitt BJ, Choi K-S. Copper-Based Catalytic Anodes To Produce 2,5-Furandicarboxylic Acid, a Biomass-Derived Alternative to Terephthalic Acid. *ACS Catal.* **8**, 1197−1206 (2018).
45. Liu W-J, Dang L, Xu Z, Yu H-Q, Jin S, Huber GW. Electrochemical Oxidation of 5-Hydroxymethylfurfural with NiFe Layered Double Hydroxide (LDH) Nanosheet Catalysts. *ACS Catal.* **8**, 5533−5541 (2018).
46. Kubota SR, Choi KS. Electrochemical Oxidation of 5-Hydroxymethylfurfural to 2,5-Furandicarboxylic Acid (FDCA) in Acidic Media Enabling Spontaneous FDCA Separation. *ChemSusChem* **11**, 2138−2145 (2018).
47. Latsuzbaia R, *et al.* Continuous electrochemical oxidation of biomass derived 5-(hydroxymethyl)furfural into 2,5-furandicarboxylic acid. *J Appl Electrochem* **48**, 611−626 (2018).
48. Li W, *et al.* Electrolyzer Design for Flexible Decoupled Water Splitting and Organic Upgrading with Electron Reservoirs. *Chem* **4**, 637−649 (2018).
49. Taitt BJ, Nam D-H, Choi K-S. A Comparative Study of Nickel, Cobalt, and Iron Oxyhydroxide Anodes for the Electrochemical Oxidation of 5-Hydroxymethylfurfural to 2,5-Furandicarboxylic Acid. *ACS Catal.* **9**, 660−670 (2018).
50. Li S, *et al.* Biomass Valorization via Paired Electrosynthesis Over Vanadium Nitride-Based Electrocatalysts. *Adv. Funct. Mater.* **29**, 1904780 (2019).
51. Chadderdon DJ, Wu LP, McGraw ZA, Panthani M, Li W. Heterostructured Bismuth Vanadate/Cobalt Phosphate Photoelectrodes Promote TEMPO-Mediated Oxidation of 5-Hydroxymethylfurfural. *ChemElectroChem* **6**, 3387−3392 (2019).

52. Chadderdon XH, Chadderdon DJ, Pfennig T, Shanks BH, Li W. Paired electrocatalytic hydrogenation and oxidation of 5-(hydroxymethyl)furfural for efficient production of biomass-derived monomers. *Green Chem.* **21**, 6210−6219 (2019).
53. Zhang N, *et al.* Electrochemical Oxidation of 5-Hydroxymethylfurfural on Nickel Nitride/Carbon Nanosheets: Reaction Pathway Determined by In Situ Sum Frequency Generation Vibrational Spectroscopy. *Angew. Chem. Int. Ed.* **58**, 15895−15903 (2019).
54. Zhang P, *et al.* Paired Electrocatalytic Oxygenation and Hydrogenation of Organic Substrates with Water as the Oxygen and Hydrogen Source. *Angew. Chem. Int. Ed.* **58**, 9155−9159 (2019).
55. Kang MJ, Park H, Jegal J, Hwang SY, Kang YS, Cha HG. Electrocatalysis of 5-hydroxymethylfurfural at cobalt based spinel catalysts with filamentous nanoarchitecture in alkaline media. *Appl. Catal. B-Environ.* **242**, 85−91 (2019).
56. Chen H, *et al.* Cu−Ni Bimetallic Hydroxide Catalyst for Efficient Electrochemical Conversion of 5-Hydroxymethylfurfural to 2,5-Furandicarboxylic Acid. *ChemElectroChem* **6**, 5797−5801 (2019).
57. Qin Q, *et al.* Electrochemical Fixation of Nitrogen and Its Coupling with Biomass Valorization with a Strongly Adsorbing and Defect Optimized Boron–Carbon–Nitrogen Catalyst. *ACS Appl. Energy Mater.* **2**, 8359−8365 (2019).
58. Li M, *et al.* Dispersive non-noble metal phosphide embedded in alumina arrays derived from layered double hydroxide precursor toward efficient oxygen evolution reaction and biomass upgrading. *J. Mater. Chem. A* **7**, 13695−13704 (2019).
59. Wang L, *et al.* Strongly Coupled 3D N-Doped MoO_2/Ni_3S_2 Hybrid for High Current Density Hydrogen Evolution Electrocatalysis and Biomass Upgrading. *ACS Appl. Mater. Interfaces* **11**, 27743−27750 (2019).
60. Chen W, *et al.* Activity origins and design principles of nickel-based catalysts for nucleophile electrooxidation. *Chem* **6**, 2974−2993 (2020).
61. Heidary N, Kornienko N. Electrochemical biomass valorization on gold-metal oxide nanoscale heterojunctions enables investigation of both catalyst and reaction dynamics with operando surface-enhanced Raman spectroscopy. *Chem. Sci.* **11**, 1798−1806 (2020).
62. Lu Y, *et al.* Hierarchically nanostructured NiO-Co_3O_4 with rich interface defects for the electro-oxidation of 5-hydroxymethylfurfural. *Sci China Chem* **63**, 980−986 (2020).
63. Yang G, *et al.* Interfacial Engineering of MoO_2-FeP Heterojunction for Highly Efficient Hydrogen Evolution Coupled with Biomass Electrooxidation. *Adv. Mater.* **32**, e2000455 (2020).
64. Lu Y, *et al.* Identifying the Geometric Site Dependence of Spinel Oxides for the Electrooxidation of 5-Hydroxymethylfurfural. *Angew. Chem. Int. Ed.* **59**, 19215−19221 (2020).
65. Huang X, *et al.* Enhancing the electrocatalytic activity of CoO for the oxidation of 5-hydroxymethylfurfural by introducing oxygen vacancies. *Green Chem.* **22**, 843−849 (2020).
66. Gao L, *et al.* NiSe@NiO_x core-shell nanowires as a non-precious electrocatalyst for upgrading 5-hydroxymethylfurfural into 2,5-furandicarboxylic acid. *Appl. Catal. B-Environ.* **261**, 118235 (2020).
67. Zhang M, Liu Y, Liu B, Chen Z, Xu H, Yan K. Trimetallic NiCoFe-Layered Double Hydroxides Nanosheets Efficient for Oxygen Evolution and Highly Selective Oxidation of Biomass-Derived 5-Hydroxymethylfurfural. *ACS Catal.* **10**, 5179−5189 (2020).
68. Park M, Gu M, Kim B-S. Tailorable Electrocatalytic 5-Hydroxymethylfurfural Oxidation and H_2 Production: Architecture–Performance Relationship in Bifunctional Multilayer Electrodes. *ACS nano* **14**, 6812−6822 (2020).
69. Cai M, Zhang Y, Zhao Y, Liu Q, Li Y, Li G. Two-dimensional metal–organic framework nanosheets for highly efficient electrocatalytic biomass 5-(hydroxymethyl)furfural (HMF) valorization. *J. Mater. Chem. A* **8**, 20386−20392 (2020).
70. Gao L, *et al.* Titanium Oxide-Confined Manganese Oxide for One-Step Electrocatalytic Preparation of 2,5-Furandicarboxylic Acid in Acidic Media. *ChemElectroChem* **7**, 4251−4258 (2020).
71. Holzhäuser FJ, Janke T, Öztas F, Broicher C, Palkovits R. Electrocatalytic Oxidation of 5-Hydroxymethylfurfural into the Monomer 2,5-Furandicarboxylic Acid using Mesostructured Nickel Oxide. *Adv. Sustain. Syst.* **4**, 1900151 (2020).
72. Kang MJ, Yu HJ, Kim HS, Cha HG. Deep eutectic solvent stabilised Co–P films for electrocatalytic oxidation of 5-hydroxymethylfurfural into 2,5-furandicarboxylic acid. *New J. Chem.* **44**, 14239−14245 (2020).
73. Song X, Liu X, Wang H, Guo Y, Wang Y. Improved Performance of Nickel Boride by Phosphorus Doping as an Efficient Electrocatalyst for the Oxidation of 5-Hydroxymethylfurfural to 2,5-Furandicarboxylic Acid. *Ind. Eng. Chem. Res.* **59**, 17348−17356 (2020).

74. Poerwoprajitno AR*, et al.* Faceted Branched Nickel Nanoparticles with Tunable Branch Length for High-Activity Electrocatalytic Oxidation of Biomass. *Angew. Chem. Int. Ed.* **59**, 15487−15491 (2020).
75. Chakraborty B, Beltrán-Suito R, Hausmann JN, Garai S, Driess M, Menezes PW. Enabling Iron-Based Highly Effective Electrochemical Water-Splitting and Selective Oxygenation of Organic Substrates through In Situ Surface Modification of Intermetallic Iron Stannide Precatalyst. *Adv. Energy Mater.* **10**, 2001377 (2020).
76. Deng X*, et al.* Coupling efficient biomass upgrading with H_2 production via bifunctional Cu_xS@NiCo-LDH core–shell nanoarray electrocatalysts. *J Mater. Chem. A* **8**, 1138−1146 (2020).
77. Deng X, Li M, Fan Y, Wang L, Fu X-Z, Luo J-L. Constructing multifunctional 'Nanoplatelet-on-Nanoarray' electrocatalyst with unprecedented activity towards novel selective organic oxidation reactions to boost hydrogen production. *Appl. Catal. B-Environ.* **278**, 119339 (2020).
78. Cai M, Ding S, Gibbons B, Yang X, Kessinger MC, Morris AJ. Nickel(ii)-modified covalent-organic framework film for electrocatalytic oxidation of 5-hydroxymethylfurfural (HMF). *Chem Commun* **56**, 14361−14364 (2020).
79. Hauke P, Klingenhof M, Wang X, de Araújo JF, Strasser P. Efficient electrolysis of 5-hydroxymethylfurfural to the biopolymer-precursor furandicarboxylic acid in a zero-gap MEA-type electrolyzer. *Cell Rep Phys Sci* **2**, 100650 (2021).
80. Lu X*, et al.* Highly Efficient Electro-reforming of 5-Hydroxymethylfurfural on Vertically Oriented Nickel Nanosheet/Carbon Hybrid Catalysts: Structure-Function Relationships. *Angew. Chem. Int. Ed.* **60**, 14528−14535 (2021).
81. Liu B*, et al.* Electrochemical upgrading of biomass-derived 5-hydroxymethylfurfural and furfural over oxygen vacancy-rich NiCoMn-layered double hydroxides nanosheets. *Green Chem.* **23**, 4034−4043 (2021).
82. Gu K*, et al.* Defect-Rich High-Entropy Oxide Nanosheets for Efficient 5-Hydroxymethylfurfural Electrooxidation. *Angew. Chem. Int. Ed.* **60**, 20253−20258 (2021).
83. Zhang R, Jiang S, Rao Y, Chen S, Yue Q, Kang Y. Electrochemical biomass upgrading on CoOOH nanosheets in a hybrid water electrolyzer. *Green Chem.* **23**, 2525−2530 (2021).
84. Wang C, Bongard HJ, Yu M, Schuth F. Highly Ordered Mesoporous Co_3O_4 Electrocatalyst for Efficient, Selective, and Stable Oxidation of 5-Hydroxymethylfurfural to 2,5-Furandicarboxylic Acid. *ChemSusChem* **14**, 5199−5206 (2021).
85. Lu Y*, et al.* Tuning the selective adsorption site of biomass on Co_3O_4 by Ir single atoms for electrosynthesis. *Adv. Mater.* **33**, 2007056 (2021).
86. Bai X-J, He W-X, Lu X-Y, Fu Y, Qi W. Electrochemical oxidation of 5-hydroxymethylfurfural on ternary metal–organic framework nanoarrays: enhancement from electronic structure modulation. *J. Mater. Chem. A* **9**, 14270−14275 (2021).
87. Deng X*, et al.* Understanding the Roles of Electrogenerated Co^{3+} and Co^{4+} in Selectivity-Tuned 5-Hydroxymethylfurfural Oxidation. *Angew. Chem. Int. Ed.* **60**, 20535−20542 (2021).
88. Zhang J, Yu P, Zeng G, Bao F, Yuan Y, Huang H. Boosting HMF oxidation performance via decorating ultrathin nickel hydroxide nanosheets with amorphous copper hydroxide islands. *J. Mater. Chem. A* **9**, 9685−9691 (2021).
89. Hu K, Zhang M, Liu B, Yang Z, Li R, Yan K. Efficient electrochemical oxidation of 5-hydroxymethylfurfural to 2,5-furandicarboxylic acid using the facilely synthesized 3D porous WO_3/Ni electrode. *Mol. Catal.* **504**, 111459 (2021).
90. Zhou H*, et al.* Selectively Upgrading Lignin Derivatives to Carboxylates through Electrochemical Oxidative C(OH)−C Bond Cleavage by a Mn-Doped Cobalt Oxyhydroxide Catalyst. *Angew. Chem. Int. Ed.* **60**, 8976−8982 (2021).
91. Kong F, Wang M. Preparation of Sulfur-Modulated Nickel/Carbon Composites from Lignosulfonate for the Electrocatalytic Oxidation of 5-Hydroxymethylfurfural to 2,5-Furandicarboxylic Acid. *ACS Appl. Energy Mater.* **4**, 1182−1188 (2021).
92. Qi Y-F, Wang K-Y, Sun Y, Wang J, Wang C. Engineering the Electronic Structure of NiFe Layered Double Hydroxide Nanosheet Array by Implanting Cationic Vacancies for Efficient Electrochemical Conversion of 5-Hydroxymethylfurfural to 2,5-Furandicarboxylic Acid. *ACS Sustain. Chem. Eng.* **10**, 645−654 (2021).
93. Hausmann JN*, et al.* Evolving Highly Active Oxidic Iron(III) Phase from Corrosion of Intermetallic Iron Silicide to Master Efficient Electrocatalytic Water Oxidation and Selective Oxygenation of 5-Hydroxymethylfurfural. *Adv. Mater.* **33**, e2008823 (2021).
94. Menezes PW*, et al.* Combination of Highly Efficient Electrocatalytic Water Oxidation with Selective

Oxygenation of Organic Substrates using Manganese Borophosphates. *Adv. Mater.* **33**, e2004098 (2021).
95. Mondal B*, et al.* Unraveling the Mechanisms of Electrocatalytic Oxygenation and Dehydrogenation of Organic Molecules to Value-Added Chemicals Over a Ni–Fe Oxide Catalyst. *Adv. Energy Mater.* **11**, 2101858 (2021).
96. Xie Y, Zhou Z, Yang N, Zhao G. An Overall Reaction Integrated with Highly Selective Oxidation of 5-Hydroxymethylfurfural and Efficient Hydrogen Evolution. *Adv. Funct. Mater.* **31**, 2102886 (2021).
97. Zhou B*, et al.* Platinum modulates redox properties and 5-hydroxymethylfurfural adsorption kinetics of $Ni(OH)_2$ for biomass upgrading. *Angew. Chem. Int. Ed.* **60**, 22908−22914 (2021).
98. Gu K*, et al.* Defect-Rich High-Entropy Oxide Nanosheets for Efficient 5-Hydroxymethylfurfural Electrooxidation. *Angew. Chem. Int. Ed.* **60**, 20253−20258 (2021).
99. Song Y*, et al.* Ultrathin layered double hydroxides nanosheets array towards efficient electrooxidation of 5-hydroxymethylfurfural coupled with hydrogen generation. *Appl. Cataly. B-Environ.* **299**, 120669 (2021).
100. Gouda L*, et al.* Tuning the selectivity of biomass oxidation over oxygen evolution on NiO−OH electrodes. *Green Chem.* **23**, 8061−8068 (2021).
101. Liu H, Lee T-H, Chen Y, Cochran EW, Li W. Paired electrolysis of 5-(hydroxymethyl)furfural in flow cells with a high-performance oxide-derived silver cathode. *Green Chem.* **23**, 5056−5063 (2021).
102. Zhou B*, et al.* Activity origin and alkalinity effect of electrocatalytic biomass oxidation on nickel nitride. *J. Energy Chem.* **61**, 179−185 (2021).
103. Wang H, Li C, An J, Zhuang Y, Tao S. Surface reconstruction of NiCoP for enhanced biomass upgrading. *J. Mater. Chem. A* **9**, 18421−18430 (2021).
104. Wang T*, et al.* Electro-catalytic oxidation of HMF to FDCA over RuO_2/MnO_2/CNT catalysts in base-free solution. *New J. Chem.* **45**, 21285−21292 (2021).
105. Wang W, Wang M. Nitrogen modulated $NiMoO_4$ with enhanced activity for the electrochemical oxidation of 5-hydroxymethylfurfural to 2,5-furandicarboxylic acid. *Catal. Sci. Technol.* **11**, 7326−7330 (2021).
106. Yang C*, et al.* Refining d-band center in $Ni_{0.85}Se$ by Mo doping: A strategy for boosting hydrogen generation via coupling electrocatalytic oxidation 5-hydroxymethylfurfural. *Chem. Eng. J.* **422**, 130125 (2021).
107. Zhang J*, et al.* In Situ Growth of Ultrathin $Ni(OH)_2$ Nanosheets as Catalyst for Electrocatalytic Oxidation Reactions. *ChemSusChem* **14**, 2935−2942 (2021).
108. Zhong Y*, et al.* Electrodeposition of hybrid nanosheet-structured $NiCo_2O_4$ on carbon fiber paper as a non-noble electrocatalyst for efficient electrooxidation of 5-hydroxymethylfurfural to 2,5-furandicarboxylic acid. *New J. Chem.* **45**, 11213−11221 (2021).
109. Zhu B, Qin Y, Du J, Zhang F, Lei X. Ammonia Etching to Generate Oxygen Vacancies on $CuMn_2O_4$ for Highly Efficient Electrocatalytic Oxidation of 5-Hydroxymethylfurfural. *ACS Sustain. Chem. Eng.* **9**, 11790−11797 (2021).
110. Liang S*, et al.* Ni_3N-V_2O_3 enables highly efficient 5-(Hydroxymethyl) furfural oxidation enabling membrane free hydrogen production. *Chem. Eng. J.* **415**, 128864 (2021).
111. Yang G*, et al.* Unraveling the mechanism for paired electrocatalysis of organics with water as a feedstock. *Nat. Commun.* **13**, 3125 (2022).
112. Zhou Z*, et al.* Strain-induced in situ formation of NiOOH species on Co−Co bond for selective electrooxidation of 5-hydroxymethylfurfural and efficient hydrogen production. *Appl. Catal. B-Environ.* **305**, 121072 (2022).
113. Lu Y*, et al.* Integrated Catalytic Sites for Highly Efficient Electrochemical Oxidation of the Aldehyde and Hydroxyl Groups in 5-Hydroxymethylfurfural. *ACS Catal.* **12**, 4242−4251 (2022).
114. Wang C, Bongard H-J, Weidenthaler C, Wu Y, Schüth F. Design and Application of a High-Surface-Area Mesoporous δ-MnO_2 Electrocatalyst for Biomass Oxidative Valorization. *Chem. Mater.* **34**, 3123−3132 (2022).
115. Sun Y, Wang J, Qi Y, Li W, Wang C. Efficient Electrooxidation of 5-Hydroxymethylfurfural Using Co-Doped Ni_3S_2 Catalyst: Promising for H_2 Production under Industrial-Level Current Density. *Adv. Sci.* **9**, e2200957 (2022).
116. Chen C*, et al.* Sustainable biomass upgrading coupled with H_2 generation over in-situ oxidized Co_3O_4 electrocatalysts. *Appl. Catal. B-Environ.* **307**, 121209 (2022).
117. Song Y*, et al.* Bifunctional integrated electrode for high-efficient hydrogen production coupled with 5-hydroxymethylfurfural oxidation. *Appl. Catal. B-Environ.* **312**, 121400 (2022).
118. Pang X, Bai H, Zhao H, Fan W, Shi W. Efficient Electrocatalytic Oxidation of 5-Hydroxymethylfurfural

Coupled with 4-Nitrophenol Hydrogenation in a Water System. *ACS Catal.* **12**, 1545−1557 (2022).
119. Bi J*, et al.* Phosphorus vacancy-engineered Ce-doped CoP nanosheets for the electrocatalytic oxidation of 5-hydroxymethylfurfural. *Chem Commun.* **58**, 7817−7820 (2022).
120. Xu X*, et al.* A Highly Efficient Nickel Phosphate Electrocatalyst for the Oxidation of 5-Hydroxymethylfurfural to 2,5-Furandicarboxylic Acid. *ACS Sustain. Chem. Eng.* **10**, 5538−5547 (2022).
121. Luo R*, et al.* A dynamic $Ni(OH)_2$-NiOOH/NiFeP heterojunction enabling high-performance E-upgrading of hydroxymethylfurfural. *Appl. Catal. B-Environ.* **311**, 121357 (2022).
122. Chen J, Wang Y, Zhou M, Li Y. Boosting the electro-oxidation of 5-hydroxymethyl-furfural on a Co-CoS_x heterojunction by intensified spin polarization. *Chem. Sci.* **13**, 4647−4653 (2022).
123. Zhang B, Fu H, Mu T. Hierarchical NiS_x/Ni_2P nanotube arrays with abundant interfaces for efficient electrocatalytic oxidation of 5-hydroxymethylfurfural. *Green Chem.* **24**, 877−884 (2022).
124. Lingxia Zheng YZ, Penghui Xu, Zhuoqing Lv, Xiaowei Shi, Huajun Zheng. Biomass upgrading coupled with H2 production via a nonprecious and versatile Cu-doped nickel nanotube electrocatalyst. *J. Mater. Chem. A* **10**, 10181−10191 (2022).
125. Jin J, Fang Y, Zhang T, Han A, Wang B, Liu J. Ultrasmall Ag nanoclusters anchored on NiCo-layered double hydroxide nanoarray for efficient electrooxidation of 5-hydroxymethylfurfural. *Sci. China Mater.* **65**, 2704−2710 (2022).
126. Woo J*, et al.* Collaborative Electrochemical Oxidation of the Alcohol and Aldehyde Groups of 5-Hydroxymethylfurfural by NiOOH and $Cu(OH)_2$ for Superior 2,5-Furandicarboxylic Acid Production. *ACS Catal.* **12**, 4078−4091 (2022).
127. Wang H, Zhou Y, Tao S. CoP-CoOOH heterojunction with modulating interfacial electronic structure: A robust biomass-upgrading electrocatalyst. *Appl. Catal. B-Environ.* **315**, 121588 (2022).
128. Bender MT, Choi KS. Electrochemical Oxidation of HMF via Hydrogen Atom Transfer and Hydride Transfer on NiOOH and the Impact of NiOOH Composition. *ChemSusChem* **15**, e202200675 (2022).
129. Lin R, Salehi M, Guo J, Seifitokaldani A. High oxidation state enabled by plated Ni-P achieves superior electrocatalytic performance for 5-hydroxymethylfurfural oxidation reaction. *iScience* **25**, 104744 (2022).
130. Lu X, Qi K, Wang D, Dai X, Qi W. The highly efficient electrocatalytic oxidation of 5-hydroxymethylfurfural on copper nanocrystalline/carbon hybrid catalysts: structure–function relations. *Catal. Sci. Technol.* **12**, 6437−6443 (2022).
131. Seo B, Woo J, Kim E, Cheong S-H, Lee DK, Lee H. Insight toward the role of Fe in layered $Ni(OH)_2$ for electrochemical oxidations of water and 5-hydroxymethylfurfural. *Catal. Commun.* **170**, 106501 (2022).
132. Wang W, Zhang Z, Wang M. Preparation of NiO-N/C composites for electrochemical oxidation of 5-hydroxymethylfurfural to 2,5-furandicarboxylic acid. *Biomass Convers. Biorefin.*, (2022).
133. Wu Y, Guo Z, Sun C, Yang Y, Li Q. Integrated experimental and DFT analysis on the efficient and green upgrading of agroforestry biomass derived furan chemicals over $NiMoO_4$-CNTs-CF electrocatalyst with bi-catalytic sites. *Ind Crops Prod* **187**, 115492 (2022).
134. Yang G*, et al.* In-situ chemical corrosive nickel foam as high-efficient electrocatalyst for 5-hydroxymethylfurfural oxidation. *Appl. Surf. Sci.* **594**, 153432 (2022).
135. Zhang Y, Xue Z, Zhao X, Zhang B, Mu T. Controllable and facile preparation of Co_9S_8–Ni_3S_2 heterostructures embedded with N,S,O-tri-doped carbon for electrocatalytic oxidation of 5-hydroxymethylfurfural. *Green Chem.* **24**, 1721−1731 (2022).
136. Zhao Z*, et al.* Bimetallic sites and coordination effects: electronic structure engineering of NiCo-based sulfide for 5-hydroxymethylfurfural electrooxidation. *Catal. Sci. Technol.* **12**, 3817−3825 (2022).
137. Zhou Y, Shen Y, Li H. Mechanistic study on electro-oxidation of 5-hydroxymethylfurfural and water molecules via operando surface-enhanced Raman spectroscopy coupled with an Fe^{3+} probe. *Appl. Catal. B-Environ.* **317**, 121776 (2022).
138. Xu L*, et al.* Salting-Out Aldehyde from the Electrooxidation of Alcohols with 100 % Selectivity. *Angew. Chem. Int. Ed.* **61**, e202210123 (2022).
139. Zhou P*, et al.* Heterogeneous-Interface-Enhanced Adsorption of Organic and Hydroxyl for Biomass Electrooxidation. *Adv. Mater.* **34**, e2204089 (2022).
140. Wang C, Wu Y, Bodach A, Krebs ML, Schuhmann W, Schüth F. A novel electrode for value-generating anode reactions in water electrolyzers at industrial current densities. *Angew. Chem. Int. Ed.* **62**, e202215804 (2022).
141. Ge R*, et al.* Selective Electrooxidation of Biomass-Derived Alcohols to Aldehydes in a Neutral Medium: Promoted Water Dissociation over a Nickel-Oxide-Supported Ruthenium Single-Atom Catalyst. *Angew. Chem. Int. Ed.* **61**, e202200211 (2022).

142. Gao L, *et al.* Nickel-vanadium-cobalt ternary layered double hydroxide for efficient electrocatalytic upgrading of 5-hydroxymethylfurfural to 2,5-furancarboxylic acid at low potential. *J. Mater. Chem. A* **10**, 21135−21141 (2022).
143. Pan X, Mei S, Liu W-J. Self-supported ultrathin Co_3O_4 nanoarray enabling efficient paired electrolysis of 5-hydroxymethylfurfural for simultaneous dihydroxymethylfuran (DHMF) and furandicarboxylic acid (FDCA) production. *Chin Chem Lett* **34**, 108034 (2022).
144. Qi J, *et al.* Paired Electrolysis of Acrylonitrile and 5-Hydroxymethylfurfural for Simultaneous Generation of Adiponitrile and 2,5-Furandicarboxylic Acid. *Catalysts* **12**, 694 (2022).
145. Sun C, Zhang D, Zhao Y, Song C, Wang D. In-suit growth of NiS quantum dots embedded in ultra-thin N,O,S-tri-doped carbon porous nanosheets on carbon cloth for high-efficient HMF oxidation coupling hydrogen evolution. *Colloids Sur., A* **650**, 129597 (2022).
146. Xing M, Zhang D, Liu D, Song C, Wang D. Surface engineering of carbon-coated cobalt-doped nickel phosphides bifunctional electrocatalyst for boosting 5-hydroxymethylfurfural oxidation coupled with hydrogen evolution. *J. Colloid Interface Sci.* **629**, 451−460 (2022).
147. Yang Z-W, Chen J-M, Qiu L-Q, Xie W-J, He L-N. Solar energy-driven electrolysis with molecular catalysts for the reduction of carbon dioxide coupled with the oxidation of 5-hydroxymethylfurfural. *Catal. Sci. Technol.* **12**, 5495−5500 (2022).
148. Zhong R, *et al.* Ultrathin polycrystalline Co_3O_4 nanosheets with enriched oxygen vacancies for efficient electrochemical oxygen evolution and 5-hydroxymethylfurfural oxidation. *Appl. Surf. Sci.* **584**, 152553 (2022).
149. Zhong Y, Ren R-Q, Wang J-B, Peng Y-Y, Li Q, Fan Y-M. Grass-like Ni_xSe_y nanowire arrays shelled with NiFe LDH nanosheets as a 3D hierarchical core–shell electrocatalyst for efficient upgrading of biomass-derived 5-hydroxymethylfurfural and furfural. *Catal. Sci. Technol.* **12**, 201−211 (2022).
150. Zhou M, Chen J, Li Y. CoP nanorods anchored on Ni_2P-NiCoP nanosheets with abundant heterogeneous interfaces boosting the electrocatalytic oxidation of 5-hydroxymethyl-furfural. *Catal. Sci. Technol.* **12**, 4288−4297 (2022).
151. Zhu Y, *et al.* Understanding the surface segregation behavior of bimetallic CoCu toward HMF oxidation reaction. *J. Energy Chem.* **74**, 85−90 (2022).
152. Wu J, *et al.* Unveiling the Adsorption Behavior and Redox Properties of PtNi Nanowire for Biomass-Derived Molecules Electrooxidation. *ACS Nano* **16**, 21518−21526 (2022).
153. Tao Y, *et al.* $Cu_xCo_{3-x}O_4$ Spinel Nanofibers for Selective Oxidation of 5-Hydroxymethylfurfural into Fuel Additives. *ACS Appl. Nano Mater.* **5**, 16564−16572 (2022).
154. Jiang M, *et al.* In-situ growth of MnO_2 on hierarchical porous carbon foam with enhanced oxygen vacancy concentration and charge transfer for efficient catalytic oxidation of 5-hydroxymethylfurfural. *Appl. Surf. Sci.* **598**, 153849 (2022).
155. Li J, *et al.* Engineering active Ni-doped Co_2P catalyst for efficient electrooxidation coupled with hydrogen evolution. *Nano Res.* **16**, 6728−6735 (2022).
156. Lu Y, *et al.* Tailoring Competitive Adsorption Sites by Oxygen-Vacancy on Cobalt Oxides to Enhance the Electrooxidation of Biomass. *Adv. Mater.* **34**, e2107185 (2022).
157. Yang Y, Xu D, Zhang B, Xue Z, Mu T. Substrate molecule adsorption energy: An activity descriptor for electrochemical oxidation of 5-Hydroxymethylfurfural (HMF). *Chem. Eng. J.* **433**, 133842 (2022).
158. Sun M, *et al.* Nitrogen-doped Co_3O_4 nanowires enable high-efficiency electrochemical oxidation of 5-hydroxymethylfurfural. *Chin Chem Lett* **33**, 385−389 (2022).
159. Sur S, Mondal R, Thimmappa R, Mukhopadhyay S, Thotiyl MO. Aqueous OH^-/H^+ dual-ion gradient assisted electricity effective electro-organic synthesis of 2,5-furandicarboxylic acid paired with hydrogen fuel generation. *J. Colloid Interface Sci.* **630**, 477−483 (2023).
160. Feng Y, Guo H, Smith RL, Jr., Qi X. Electrocatalytic oxidation of 5-hydroxymethylfurfural to 2,5-furandicarboxylic acid via metal-organic framework-structured hierarchical Co_3O_4 nanoplate arrays. *J. Colloid Interface Sci.* **632**, 87−94 (2023).
161. Pang X, Zhao H, Huang Y, Luo B, Bai H, Fan W. Electrochemically induced $NiOOH/Ag^+$ active species for efficient oxidation of 5-hydroxymethylfurfural. *Appl. Surf. Sci.* **608**, 155152 (2023).
162. Wu T, *et al.* Surface-confined self-reconstruction to sulfate-terminated ultrathin layers on $NiMo_3S_4$ toward biomass molecule electro-oxidation. *Appl. Catal. B-Environ.* **323**, 122126 (2023).
163. Zhang D, Xing M, Mou X, Song C, Wang D. Deep eutectic solvent induced ultrathin Co_4N/N-doped carbon nanosheets self-supporting electrode for boosting hydrogen evolution integrated with biomass electrooxidation. *Appl. Surf. Sci.* **608**, 155283 (2023).

164. Zhou P, *et al.* CeO_2 as an "electron pump" to boost the performance of Co_4N in electrocatalytic hydrogen evolution, oxygen evolution and biomass oxidation valorization. *Appl. Catal. B-Environ.* **325**, 122364 (2023).
165. Yang S, *et al.* Anionic defects engineering of $NiCo_2O_4$ for 5-hydroxymethylfurfural electrooxidation. *Chem. Eng. J.* **457**, 141344 (2023).
166. Krebs ML, Bodach A, Wang C, Schüth F. Stabilization of alkaline 5-HMF electrolytes via Cannizzaro reaction for the electrochemical oxidation to FDCA. *Green Chem.* **25**, 1797−1802 (2023).
167. Feng Y, Yang K, Smith RL, Qi X. Metal sulfide enhanced metal–organic framework nanoarrays for electrocatalytic oxidation of 5-hydroxymethylfurfural to 2,5-furandicarboxylic acid. *J. Mater. Chem. A* **11**, 6375−6383 (2023).
168. Zhang K, *et al.* An efficient electrocatalytic system composed of nickel oxide and nitroxyl radical for the oxidation of bio-platform molecules to dicarboxylic acids. *J. Energy Chem.* **80**, 58−67 (2023).
169. Wang H, *et al.* Boosting 5-hydroxymethylfurfural electrooxidation in neutral electrolytes via TEMPO-enhanced dehydrogenation and OH adsorption. *Chinese J. Catal.* **46**, 148−156 (2023).
170. Gong W, *et al.* Ni-Co Alloy Nanoparticles Catalyze Selective Electrochemical Coupling of Nitroarenes into Azoxybenzene Compounds in Aqueous Electrolyte. *ACS nano* **17**, 3984−3995 (2023).
171. Li S, *et al.* Doped Mn Enhanced NiS Electrooxidation Performance of HMF into FDCA at Industrial-Level Current Density. *Adv. Funct. Mater.* **33**, 2214488 (2023).
172. Yang Z, Zhang B, Yan C, Xue Z, Mu T. The pivot to achieve high current density for biomass electrooxidation: Accelerating the reduction of Ni^{3+} to Ni^{2+}. *Appl. Catal. B-Environ.* **330**, 122950 (2023).
173. Zhao L, Du S, Gong R, Jia W, Chen Z, Ren Z. CoO−Co Heterojunction Covered with Carbon Enables Highly Efficient Integration of Hydrogen Evolution and 5-Hydroxymethylfurfural Oxidation. *Molecules* **28**, 3040 (2023).
174. Zhao G, *et al.* Electrochemical Oxidation of 5-Hydroxymethylfurfural on CeO_2-Modified Co_3O_4 with Regulated Intermediate Adsorption and Promoted Charge Transfer. *Adv. Funct. Mater.* **33**, 2213170 (2023).
175. Liu S, Dou S, Meng J, Liu Y, Liu Y, Yu H. Efficient biobased carboxylic acids synthesis by synergistic electrocatalysis of multi-active sites on bimetallic Cu-Co oxide/oxyhydroxide. *Appl. Catal. B-Environ.* **331**, 122709 (2023).
176. Ye F, *et al.* The role of oxygen-vacancy in bifunctional indium oxyhydroxide catalysts for electrochemical coupling of biomass valorization with CO_2 conversion. *Nat. Commun.* **14**, 2040 (2023).
177. Bi J, *et al.* Cu_2P_7-CoP Heterostructure Nanosheets Enable High-Performance of 5-Hydroxymethylfurfural Electrooxidation. *Chemistry* **29**, e202300973 (2023).
178. Zhong R, *et al.* Room-temperature fabrication of defective CoOxHy nanosheets with abundant oxygen vacancies and high porosity as efficient 5-hydroxymethylfurfural oxidation electrocatalysts. *Green Chem.* **25**, 4674−4684 (2023).
179. Muiuane VP, Ferreira M, Bignet P, Bettencourt AP, Parpot P. Production of formic acid from biomass-based compounds using a filter press type electrolyzer. *J. Energy Environ. Chem. Eng.* **1**, 1237−1244 (2013).
180. Wang C, Wu Y, Bodach A, Krebs ML, Schuhmann W, Schuth F. A Novel Electrode for Value-Generating Anode Reactions in Water Electrolyzers at Industrial Current Densities. *Angew. Chem. Int. Ed.* **62**, e202215804 (2022).
181. He Z, *et al.* Promoting biomass electrooxidation via modulating proton and oxygen anion deintercalation in hydroxide. *Nat. Commun.* **13**, 3777 (2022).
182. Xu Y, *et al.* Integrating electrocatalytic hydrogen generation with selective oxidation of glycerol to formate over bifunctional nitrogen-doped carbon coated nickel-molybdenum-nitrogen nanowire arrays. *Appl. Catal. B-Environ.* **298**, 120493 (2021).
183. Qian Q, *et al.* Electrochemical Biomass Upgrading Coupled with Hydrogen Production under Industrial-level Current Density. *Adv. Mater.* **35**, e2300935 (2023).
184. Goetz MK, Bender MT, Choi KS. Predictive control of selective secondary alcohol oxidation of glycerol on NiOOH. *Nat. Commun.* **13**, 5848 (2022).
185. He Z, *et al.* Promoting biomass electrooxidation via modulating proton and oxygen anion deintercalation in hydroxide. *Nat. Commun.* **13**, 3777 (2022).
186. Oh LS, *et al.* How to Change the Reaction Chemistry on Nonprecious Metal Oxide Nanostructure Materials for Electrocatalytic Oxidation of Biomass-Derived Glycerol to Renewable Chemicals. *Adv. Mater.* **35**, e2203285 (2023).
187. Zhu Y, *et al.* Biphasic Transition Metal Nitride Electrode Promotes Nucleophile Oxidation Reaction for Practicable Hybrid Water Electrocatalysis. *Adv. Funct. Mater.* **33**, 2300547 (2023).
188. Wang Y, *et al.* Efficient Electrocatalytic Oxidation of Glycerol via Promoted OH* Generation over

Single-Atom-Bismuth-Doped Spinel Co_3O_4. *ACS Catal.* **12**, 12432−12443 (2022).
189. Zhao B*, et al.* Anode-cathode interchangeable strategy for in situ reviving electrocatalysts' critical active sites for highly stable methanol upgrading and hydrogen evolution reactions. *Appl. Catal. B-Environ.* **305**, 121082 (2022).
190. Zhu B*, et al.* Unraveling a bifunctional mechanism for methanol-to-formate electro-oxidation on nickel-based hydroxides. *Nat. Commun.* **14**, 1686 (2023).
191. Sluiter A, Hames B, Ruiz R, C. Scarlata, Sluiter J, Templeton D. Determination of Sugars, Byproducts, and Degradation Products in Liquid Fraction Process Samples. *National Renewable Energy Laboratory* **NREL/TP-510-42623**, http://www.nrel.gov/docs/gen/fy08/42623.pdf (2006).
